# MetaPGN: a pipeline for construction and graphical visualization of annotated pangenome networks

**DOI:** 10.1093/gigascience/giy121

**Published:** 2018-10-02

**Authors:** Ye Peng, Shanmei Tang, Dan Wang, Huanzi Zhong, Huijue Jia, Xianghang Cai, Zhaoxi Zhang, Minfeng Xiao, Huanming Yang, Jian Wang, Karsten Kristiansen, Xun Xu, Junhua Li

**Affiliations:** 1 School of Biology and Biological Engineering, South China University of Technology, Building B6, 382 Zhonghuan Road East, Guangzhou Higher Education Mega Center, Guangzhou 510006, China; 2 BGI-Shenzhen, Building 11, Beishan Industrial Zone, Yantian, Shenzhen 518083, China; 3 China National GeneBank, BGI-Shenzhen, Dapeng New District, Shenzhen 518120, China; 4 Shenzhen Key Laboratory of Human commensal microorganisms and Health Research, BGI-Shenzhen, Building 11, Beishan Industrial Zone, Yantian, Shenzhen 518083, China; 5 Laboratory of Genomics and Molecular Biomedicine, Department of Biology, University of Copenhagen, Copenhagen Biocenter, Ole MaalØes Vej 5, DK-2200 Copenhagen N, Denmark; 6 James D. Watson Institute of Genome Sciences, No. 51, Zhijiang Road, Xihu District, Hangzhou 310058, China

**Keywords:** pangenome, visualization, metagenomics

## Abstract

Pangenome analyses facilitate the interpretation of genetic diversity and evolutionary history of a taxon. However, there is an urgent and unmet need to develop new tools for advanced pangenome construction and visualization, especially for metagenomic data. Here, we present an integrated pipeline, named MetaPGN, for construction and graphical visualization of pangenome networks from either microbial genomes or metagenomes. Given either isolated genomes or metagenomic assemblies coupled with a reference genome of the targeted taxon, MetaPGN generates a pangenome in a topological network, consisting of genes (nodes) and gene-gene genomic adjacencies (edges) of which biological information can be easily updated and retrieved. MetaPGN also includes a self-developed Cytoscape plugin for layout of and interaction with the resulting pangenome network, providing an intuitive and interactive interface for full exploration of genetic diversity. We demonstrate the utility of MetaPGN by constructing *Escherichia coli* pangenome networks from five *E. coli* pathogenic strains and 760 human gut microbiomes,revealing extensive genetic diversity of *E. coli* within both isolates and gut microbial populations. With the ability to extract and visualize gene contents and gene-gene physical adjacencies of a specific taxon from large-scale metagenomic data, MetaPGN provides advantages in expanding pangenome analysis to uncultured microbial taxa.

## Introduction

The concept of the pangenome, defined as the full complement of genes in a clade, was first introduced by Tettelin et al. in 2005 [1]. Pangenome analyses of a species now provide insights into core- and accessory-genome profiles, within-species genetic diversity, evolutionary dynamics, and niche-specific adaptions. A number of methods and tools have, to date, been proposed for pangenome analysis on genomic or metagenomic data.

**Table 1:** Comparison of several pangenome analysis methods

	Input		Output			Functionality	
Method	Isolate genomes	Metagenomes	Gene content	Gene-gene adjacency	Network	Biological annotation	Interactive visualization
GET_HOMOLOGUES [2] and PGAP [3]	Yes	No	Yes	No	No	Yes	No
GenoSets [4], PGAT [5], PEGR [6], EDGAR [7], GenomeRing [8]	Yes	No	Yes	Yes	No	Yes	No
PanViz [9]	Yes	No	Yes	Yes	No	Yes	Yes
SplitMEM [10] and a tool introduced by Baier et al. [11]	Yes	No	Yes	Yes	Yes	No	Yes
PanPhlAn [12], MIDAS [13], and a method introduced by Farag et al. [16]	No	Yes	Yes	No	No	Yes	No
MetaPGN	Yes	Yes	Yes	Yes	Yes	Yes	Yes

Typical pangenome tools such as GET_HOMOLOGUES [2] and PGAP [3] mainly focus on analyzing homologous gene families and calculating the core/accessory genes of a given taxon. However, these tools cannot provide the variations of gene-gene physical relationships. Tools like GenoSets [4], PGAT [5], PEGR [6], EDGAR [7], GenomeRing [8], and PanViz [9] are developed to generate a linear or circular presentation of compared genomes, which can indicate the physical relationships between genomic sequences or genes. However, in the linear or circular representations generated by these tools, the same homologous region is visualized multiple times and shown on separate input genomes. Hence, it will be difficult for users to track a homologous region among the input genomes, especially when there is a large number of homologous regions and input genomes.

Pangenomes built using *de Bruijn* graph, such as SplitMEM [10] and a tool introduced by Baier et al. [11], partly solve the problems listed above. In the resulting graph generated with these tools, the complete pangenome is represented in a compact graphical representation such that the core/accessory status of any genomic sequences is immediately identifiable, along with the context of the flanking sequences. This strategy enables powerful topological analysis of the pangenome that is not possible from a linear/circular representation. Nevertheless, tools based on the *de Bruijn* graph algorithm can only construct a compact network comprised of core/accessory genomic sequences instead of genes, which means retrieving or updating functional information in downstream analysis will be difficult. Furthermore, these tools do not visualize the constructed *de Bruijn* graph and provide an interactive interface for users to explore the graph.

Moreover, all the above-mentioned tools analyze pangenomes via genomic data, which require organisms isolated from the environment and cultured *in vitro*. Recent advances in metagenomics have led to a paradigm shift in pangenome studies from a limited quantity of cultured microbial genomes to large-scale metagenomic datasets containing huge potential for functional and phylogenetic resolution from the still uncultured taxa. Several existing tools dealing with metagenomic data are based on constructed pangenomes and cannot utilize the abundant gene resources contained in metagenomes to extend the pangenomes in question. For example, PanPhlAn [12], MIDAS [13], and a pipeline introduced by Delmont and Eren [14] map reads onto a reference pangenome to describe the pattern of the presence/absence of genes in metagenomes. As another example, Kim et al. [15] clustered genes predicted from metagenomic contigs with *Bacillus* core genes for profiling the *Bacillus* species in the microbiomes. Recently, Farag et al. [16] aligned metagenome contigs with reference genomes for identification of *“Latescibacteria”* genomic fragments. Even though this strategy can theoretically recruit sequences not present in the reference genomes, it is likely to filter out *“Latescibacteria”* genomic fragments with structural variations compared to the reference ones. Furthermore, all of these aforementioned methods that use metagenomic data do not organize the pangenome using a network, which is essential for efficient storage and visualization of pangenomes constructed from metagenomic data.

Here, we introduce an integrated pipeline (MetaPGN) for network-based construction and visualization of prokaryotic pangenomes for both isolated genomes and metagenomes. Given genomic or metagenomic assemblies and a reference genome of a taxon of interest, MetaPGN derives a pangenome network for integrating genes (nodes) and gene-gene adjacencies (edges) belonging to a given taxon. MetaPGN also includes a specific Cytoscape plugin for layout of and interaction with the resulting pangenome network, providing an intuitive and interactive interface for the exploration of gene diversity. For example, in the visualized network in Cytoscape, users can specify gene annotations, customize the appearance of nodes and edges, and search and concentrate on genes of certain functions. We applied MetaPGN on assemblies from five pathogenic *Escherichia coli* strains and 760 human gut microbiomes, with *E. coli* K-12 substr. MG1655 (*E. coli* K-12) being the reference genome. Our results showed that by taking gene adjacency into account and visualizing the pangenome network in a well-organized manner, MetaPGN can assist in illustrating genetic diversity in genomic or metagenomic assemblies graphically and conveniently.

## Results

### General workflow

**Figure 1:**
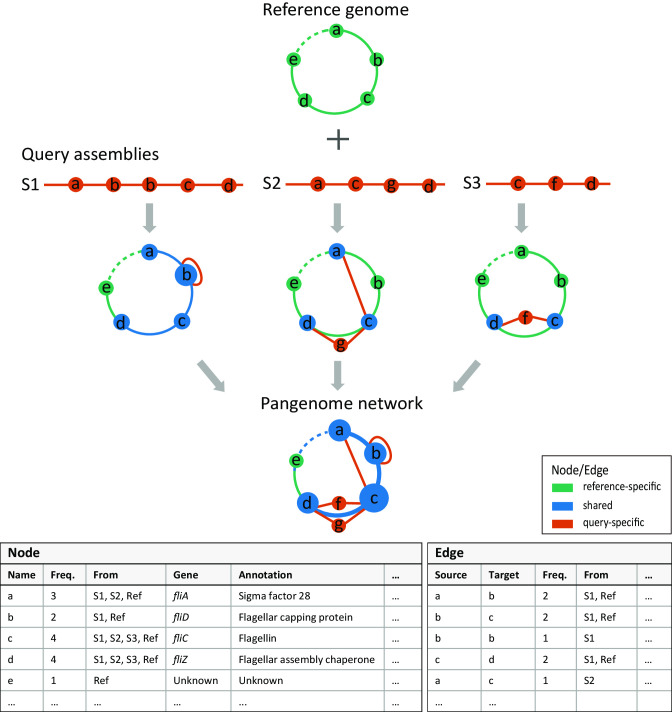
An overview of the MetaPGN pipeline: from assemblies to a pangenome network. Gene prediction is performed on query assemblies. The resulting genes are clustered, after which genes in the same cluster are represented by the longest sequence of this cluster, called the representative gene (node a-g). All of these representative genes are then aligned against genes on the given reference genome. From the alignment result, genes shared between the representative gene set and the reference gene set are defined as “shared” genes (blue). The remaining representative and reference genes, other than those shared genes, are defined as “query-specific” genes (red) and “reference-specific” genes (green), respectively. Pairwise gene physical adjacency of representative genes on the query assemblies and of reference genes are then extracted, and status for each adjacency of being “shared” (blue), “query-specific” (red), or “reference-specific” (green) is determined. Finally, based on the recruited assemblies and the reference genome, a pangenome network is generated. Each node stands for a reference gene or a representative gene on the recruited assemblies; two nodes are connected by an edge if they are physically adjacent on the recruited assemblies or the reference genome. The weight of a node or an edge is its occurrence frequency on all of the recruited assemblies and the reference genome (Methods section). The pangenome network is then visualized in Cytoscape with a self-developed plugin (Methods section) for a better arrangement. Biological information of nodes and edges, such as gene name and annotation, can be easily retrieved in the interactive user interface in Cytoscape.

MetaPGN accepts genome or metagenome assemblies as input (query assemblies) and requires a reference genome for recruitment of the query assemblies and as the skeleton of the pangenome network. The MetaPGN pipeline can be divided into two main parts: construction of a pangenome network comprised of representative genes, including gene prediction, gene redundancy elimination, gene type determination, assembly recruitment (for metagenomic assemblies), pairwise gene adjacency extraction, and pangenome network generation; and visualization of the pangenome network in an organized way, where nodes represent genes and edges indicate gene adjacencies in Cytoscape [17] with a self-developed plugin (Fig. 1, Supplementary Fig. S1, Methods Section). From the resultant pangenome network, the degree of similarity among homologous genes, as well as their genomic context, is easily visible. Of note, users can further add and update annotation for nodes and edges in the networks, based on which elements of interest can be accessed conveniently.

### Pangenome network of 5 pathogenic *E. coli* genomes

In order to demonstrate its potential in studying microbial genetic diversity and phenotype-genotype relationships, we first applied MetaPGN on genomes of five pathogenic *E. coli* isolates: *E. coli* O26: H11 str. 11 368, *E. coli* O127: H6 E2348/69, *E. coli* O157: H7 str. EDL933, *E. coli* O104: H4 str. 2011C-3493, and *E. coli* 55 989. A commensal *E. coli* strain, K-12 substr. MG1655 (Supplementary Table S1), was chosen as the reference genome in this instance and in all examples shown below.

**Figure 2:**
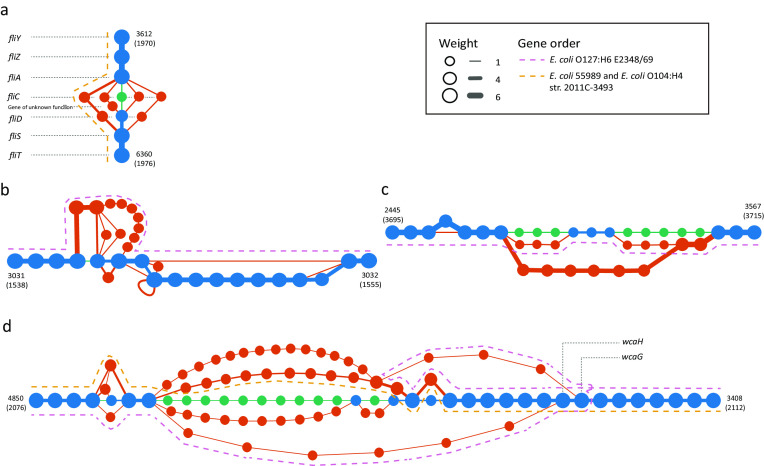
Subgraphs of highly variable genes in the pangenome network of five pathogenic *E. coli* strains (manually arranged). **(a)** A cluster of flagellar genes. **(b)** A cluster containing outer membrane protein-coding genes. **(c)** A cluster of genes responsible for biosynthesis of the O antigen. **(d)** Another cluster of O antigen-related genes. Green, blue, and red nodes and edges denote reference-specific, shared, and query-specific genes and gene adjacencies, respectively. Size of nodes and thickness of edges indicate their weight (occurrence frequency). Numbers alongside shared genes are their indexes in the representative gene set.

A pangenome network consisting of 9,161 nodes and 11,788 edges (Supplementary Table S3, Supplementary File 2) was constructed and visualized (Methods section). Based on the well-visualized pangenome network along with functional annotation, we can now graphically observe the extent of variations of certain genes, as well as their genomic context. For example, when focusing on a cluster of flagellar genes (Fig. 2a), we found that *fliC* sequences encoding the filament structural protein (H-antigen) and *fliD* sequences encoding the filament capping protein are highly divergent, with nucleotide sequence identity <95% and/or overlap <90% among these *E. coli* strains (Methods section). In contrast, four genes encoding chaperones (*fliS, fliT, fliY, fliZ*) and a gene related to regulation of expression of flagellar components (*fliA*) are conserved (nucleotide sequence identity ≥95% and overlap ≥90%) over all the *E. coli* strains investigated. A gene (270 bp) encoding a hypothetical protein is uniquely presented between *fliC* and *fliD* in *E. coli* O157: H7 str. EDL933.

In a fimbria protein-related gene cluster, compared to the reference *E. coli* strain, all five pathogenic strains possess several genes located between two conserved genes encoding an outer membrane protein and a regulatory protein. *Escherichia coli* O127: H6 E2348/69 uniquely exhibits more genes encoding proteins of unknown functions (Fig. 2b).

For a gene cluster responsible for the biosynthesis of lipopolysaccharides (LPS), *E. coli* O127: H6 E2348/69 shares three genes with the reference strain that differentiate from the other four pathogenic strains (Fig. 2c). For another gene cluster of related function, the *E. coli* O127: H6 E2348/69 also shows a strain-specific duplication event of two genes involved in colanic acid (CA) synthesis (wcaH and wcaG, denoted by a purple dashed line in Fig. 2d). It has been demonstrated that CA can modify LPS, generating a novel form (M_LPS_) that may enhance survival of *E. coli* in different ways [18]. The two wcaH genes in *E. coli* O127: H6 E2348/69 may result in different functions for CA formation and novel survival mechanisms, despite sharing a high degree of similarity (99.1% identity). In addition, the German outbreak *E. coli* O104: H4 str. 2011C-3493 shares identical nodes and edges in the flagellar-related gene cluster (Fig. 2a) and the O antigen-related gene cluster with a historical *E. coli* 55 989 (Fig. 2d), suggesting a close evolutionary relationship between these strains as previously reported [19, 20].

These results demonstrate the feasibility of MetaPGN for construction and visualization of microbial pangenomes in an organized way. Moreover, by involving genomic adjacency and offering easy-to-achieve biological information, MetaPGN provides a convenient way to assist biologists in exposing genetic diversity for genes of interest among the organisms under study.

### Pangenome network of *E. coli* in 760 metagenomes

Moving beyond surveying the pangenome network of isolate genomes, we applied MetaPGN in metagenomic datasets to interrogate the *E. coli* pangenome network on a grander scale. Assemblies of 760 metagenomes sequenced in the Metagenomics of the Human Intestinal Tract project [21–24], which contained 8,096,991 nonredundant genes with annotations [24], were collected. As metagenome assemblies are from varied taxa, it is necessary to recruit assemblies of the targeted taxon before construction of the pangenome network. In this study, metagenome assemblies were recruited using a gene alignment-based strategy, which was assessed with mock datasets (Methods section). With the recruited assemblies, a pangenome network consisting of 9,406 nodes and 14,676 edges (Supplementary Table S3, Supplementary File S3) was generated and visualized after refinement (Methods section).

**Figure 3:**
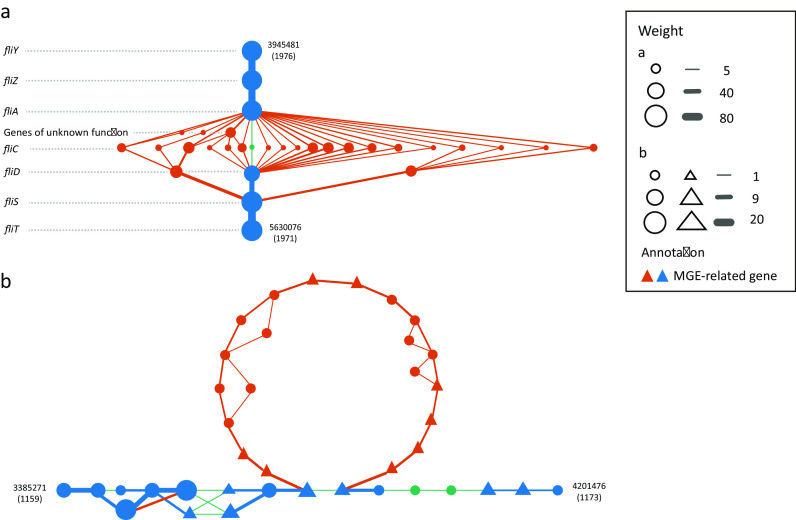
Two subgraphs of the pangenome network of *E. coli* constructed from 760 metagenomes (manually arranged). **(a)** A cluster of flagellar genes. **(b)** A cluster of genes containing mobile genetic element (MGEs). Green, blue, and red nodes and edges denote reference-specific, shared, and query-specific genes and gene adjacencies. Triangles represent MGEs. Size of nodes and thickness of edges indicate their weight (occurrence frequency). Numbers alongside shared genes are their indexes in the representative gene set.

Based on annotation, we first searched flagellin-related genes in this network. We found that the pattern of adjacencies among these genes was similar to that in the pangenome network of the five pathogenic *E. coli* genomes: *fliC* and *fliD* are hypervariable while *fliT, fliY, fliZ*, and *fliA* are very conserved among these 760 samples. However, some genes of unknown function locate between *fliC* and *fliA* (Fig. 3a) instead of between *fliC* and *fliD* in the pangenome network of the five pathogenic *E. coli* strains (Fig. 2a).

We then investigated mobile genetic elements (MGEs) in this pangenome network, as they can induce various types of genomic rearrangements [25]. Of the 362 nodes (∼4%) annotated as MGE-related (according to Cluster of Orthologous Groups annotation done in reference [24]), many were flanked by shared genes on different *E. coli* genomes. In a region of the network, a gene cluster containing MGEs is query specific, indicating there might be genomic rearrangements caused by strain-specific MGEs within the *E. coli* species (Fig. 3b). In another part of the network harboring MGEs, we observed that several branches of non-MGE genes are inserted between two MGEs, which may imply a mutation hot spot within the region or the existence of MGEs as yet undescribed (Supplementary Fig. S1).

Application of MetaPGN in large-scale metagenomic data generated an *E. coli* pangenome network that could hardly be constructed from isolated genomes. As demonstrated here, the assembly recruitment-based, well-organized, and visualized pangenome network can greatly expand our understanding of the genetic diversity of a taxon, although future efforts in bioinformatic and experimental analyses are needed to verify and extend these findings.

### Assessment of pangenome networks derived from metagenomes

**Figure 4:**
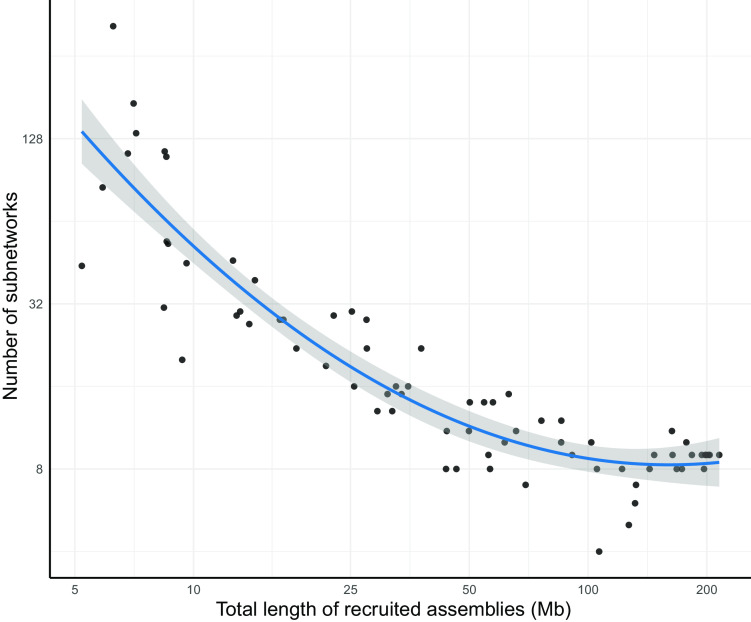
Number of subnetworks in pangenome networks derived from varying sizes of recruited assemblies. The *x*-axis indicates total length of recruited assemblies for each sub-dataset and the *y*-axis represents the number of subnetworks in the pangenome network derived from each sub-dataset. The curve was fitted for the scatters using the “loess” smoothing method in R [26]. The shaded area displays the 95% confidential intervals of the curve. Axes are log2-transformed.

Affected by the complexity of microbial communities, limitations in sequencing platforms, and imperfections of bioinformatic algorithms, a genomic sequence of an organism is frequently split into dozens of assemblies when assembled from metagenomic reads. Because of this, a pangenome network recovered from a limited number of assemblies is likely to be segmented compared to a complete genome. To propose a minimum size of assemblies for getting an approximately complete connected pangenome network, we assessed the completeness of *E. coli* pangenome networks derived from varying sizes of recruited assemblies (Methods section). As shown in Fig. 4, the count of connected subnetworks drops dramatically with the total length of recruited assemblies, increasing from 5 Mb to 50 Mb (roughly from $1{\rm{\ }} \times $ to $10{\rm{\ }} \times $ of a *E. coli* genome), then barely changes even when all recruited assemblies of the dataset (215 Mb, from 760 samples) are used. Based on this analysis, a minimum size of recruited assemblies 10-fold that of the studied genome is required to generate a relatively intact pangenome network when constructed from metagenomes.

## Discussion

Since first coined more than a decade ago, pangenome analysis has provided a framework for studying the genomic diversity within a species. Current methods for pangenome analyses mainly focus on gene contents but ignore their genomic context, as well as having shortages in pangenome visualization. In addition, available methods are usually designed for genomic data and not capable of constructing pangenomes from metagenomics data. To fill these gaps, our MetaPGN pipeline takes genome or metagenome assemblies as input, uses gene contents as well as pairwise gene adjacency to generate a compact graphical representation for the gene network based on a reference genome, and visualizes the network in Cytoscape with a self-developed plugin (Fig. 1, Supplementary Fig. S2).

From the two MetaPGN-derived *E. coli* pangenome networks, we can directly observe the diversity of genes among the five pathogenic *E. coli* strains and 760 human gut microbiomes with respect to the reference genome. For instance, we found that nucleotide sequences of the *fliC* gene, which carries H-antigen specificity, were highly divergent among the five pathogenic *E. coli* assemblies (Fig. 2a). These *fliC* sequences were more varied in the 760 human gut microbiomes (Fig. 3a). In addition, genes required for the synthesis of O-antigen and outer membrane proteins showed greater diversity in the pangenome network of the five *E. coli* strains (Fig. 2c, 2d). These results are in agreement with previous findings on H- and O-antigen specificity-related genes [27–31].

We also showed that the locations of genes of unknown function are identified when gene adjacency is incorporated into the construction and visualization of pangenomes; this may be helpful for the inference of their biological functions. For example, in both pangenome networks, we found genes of unknown function locating between the *fliC* gene and other flagellin-related genes (Fig. 2a, located between *fliC* and *fliD*; Fig. 3a, located between *fliC* and *fliA*), indicating that these functionally unknown genes may play a role in flagellin biosynthesis [32], although further experimental trials are needed to prove this point. Additionally, from the pangenome network of the five *E. coli* strains, we observed a variation in *E. coli* O127: H6 E2348/69, which was shown to stem from a duplication event of two genes involved in CA synthesis (*wcaH* and *wcaG*, Fig. 2d). This finding indicates that knowledge of genomic adjacency may also shed light on structural variations among the input assemblies. Furthermore, genomic adjacency may further help in finding possible functional sequences that are associated with structural variations, as Delihas [33] and Wang et al. [34] reported on repeat sequences concentrated at the breakpoints of structural variations. Studying genomic adjacency can also improve the discovery of potential functional modules, as Doron et al. [35] systematically discovered bacterial defensive systems by examining gene families enriched next to known defense genes in prokaryotic genomes. These examples illustrate the value of including gene adjacencies in visualizing a pangenome to retrieve biological information. Although the examples shown in this study use the genome of a commensal *E. coli* strain for assembly recruitment and network arrangement, users can specify the reference genome when applying MetaPGN. Epidemiologists can use MetaPGN to compare assemblies of outbreak strains or viruses, such as *Vibrio cholerae* or Ebola virus, with those of some well-studied pathogenic strains to find novel variations involved in pathogenesis, which may further provide candidate targets for drug and vaccine design [36, 37].

Genomic variants of intestinal bacteria were previously found to be correlated with different diseases. For example, the inclusion of a pathogenicity island (BfPAI) in *Bacteroides fragilis* distinguished enterotoxigenic strains (ETBF) from nontoxigenic strains by the ability of ETBF to secrete a zinc-dependent metalloprotease toxin that can induce inflammatory diarrhea and even colon carcinogenesis [38, 39]. Furthermore, Scher et al. performed shotgun sequencing on fecal samples from newly onset untreated rheumatoid arthritis (NORA) patients and healthy individuals and identified several NORA-specific *Prevotella copri* genes [40]. Hence, pangenome networks built from metagenomes of patients and healthy subjects may aid in detecting associated genomic variants of a certain species.

It should be noted that in this pipeline, we compare genes depending on nucleotide-level sequence identity and overlap; genes with ≥95% identity and ≥90% overlap are considered to be the same gene. However, genes sharing the same function may not satisfy this criterion (≥95% identity and ≥90% overlap), and protein encoded by these genes may exhibit more similarity due to different codon usage. Therefore, we intend to cluster genes by comparing their nucleotide sequences as well as the amino acid sequences in future developments of MetaPGN. Furthermore, the current MetaPGN pipeline does not consider other genomic features or physical distances between genes in constructing the pangenome network. Thus, differences in other genomic features such as ribosomal binding site (RBS) sequences [41, 42] and distances between the RBS and start codons [43] may result in distinct phenotypes. Accordingly, users may include such information when analyzing pangenome networks. To conclude, MetaPGN enables direct illustration of genetic diversity of a species in pangenome networks and improves our understanding of genotype-phenotype relationships and the evolutionary history of microorganisms.

## Methods

### Pangenome network construction in MetaPGN

Gene prediction of query assemblies is performed using MetaGeneMark (version 2.8) [44]. In order to eliminate redundancy, the resultant genes are clustered by CD-HIT (version 4.5.7) [45] with identity ≥95% and overlap ≥90, and genes in the same cluster are represented by the longest sequence of the cluster,the Basic Local Alignment Search Tool (BLAST)-like alignment tool (BLAT), which is termed the representative gene. Representative genes of all clusters are subsequently aligned against genes on the given reference genome using BLAT (version 34) [46]. From the alignment result, genes shared between the representative gene set and the reference gene set with identity ≥95% and overlap ≥90% are defined as “shared genes.” The remaining representative and reference genes other than those shared genes are defined as “query-specific genes” and “reference-specific genes,” respectively. For metagenomic datasets, a gene alignment- based strategy is used for assembly recruitment. Pairwise gene physical adjacency of representative genes on the query assemblies and of reference genes are then extracted, and the status for each adjacency of being “shared,” “query specific,” or “reference specific” is determined. Finally, based on the recruited assemblies and the reference genome, an initial pangenome network is generate. Each node stands for a reference gene or a representative gene on the recruited assemblies; two nodes are connected by an edge if they are physically adjacent on the recruited assemblies or on the reference genome; and the weight of a node or an edge denotes its occurrence frequency on all of the recruited assemblies and the reference genome.

### Pangenome network visualization in MetaPGN

The following preprocessing work on the initial pangenome network was implemented before visualization: (1) the initial pangenome network was refined by removing isolated networks (networks not connected with the backbone) and tips (nodes only connected with another node) and (2) nodes and edges were added with some extra attributes, such as the status of the nodes and edges (query specific, reference specific, or shared), whether the genes for the nodes were phage-, plasmid-, CRISPR-related genes and so on (Supplementary Table S3). Users can specify the attributes of nodes and edges according to their own datasets.

We then used a self-developed Cytoscape plugin to visualize the pangenome network in an organized way. (Supplementary Text 2 in Supplementary File S1 illustrates how to install and use the plugin in Cytoscape.) Our algorithm for organizing nodes in the network is as follows:

- Construct a circular skeleton for the pangenome network with shared nodes and reference-specific nodes, according to positions of their related reference genes on the reference genome. If there are two or more representative genes similar to the same reference gene (≥95% identity and ≥90% overlap), use one of these representative genes to construct the skeleton and place the others on both sides of the skeleton in turn (Supplementary Fig. S2a).
- Arrange query-specific nodes region by region, including,
  - Select query-specific nodes in a region spanning less than 30 nodes in the skeleton (see Supplementary Text 3 in Supplementary File S2 for more details).
  - Arrange these query-specific nodes as follows,
    1. For those that directly link with two nodes on the skeleton, place them on the bisector of the two skeleton nodes. If there are two or more query-specific nodes directly linking with the same pair of nodes on the skeleton, place them on both sides of the bisector of these pair of skeleton nodes in turn (Supplementary Fig. S2b).
    2. Among the remaining nodes, for those that directly link with two placed nodes, place them on the bisectors of the placed ones. Iterate this step five times (Supplementary Fig. S2c).
    3. For the remaining nodes, place them into an arc without moving the placed nodes (Supplementary Fig. S2d), alternatively place them one by one starting near a placed node (Supplementary Fig. S2e).

### Construction and visualization of the 5-*E. coli*-genome pangenome network

Genes were extracted from the complete genome for each strain (Supplementary Table S1). With *E. coli* K-12 as the reference, a pangenome network was generated for these five *E. coli* strains using our MetaPGN tool (RRID:SCR_016472). In the visualization of this pangenome network, we used green, blue, and red to denote a reference-specific, shared, and query-specific node or edge, respectively, and specified sizes of nodes and widths of edges with their occurrence frequency in the input genomes.

### Assessment of the gene alignment-based assembly recruitment strategy

Traditionally, an assembled sequence is considered to be derived from a genome if the sequence aligns with the genome over certain cutoffs (genome alignment-based strategy). Given that basic elements in a pangenome network are genes (nodes), to exploit information generated in gene redundancy elimination and to reduce computation time, we introduce a gene alignment-based strategy for recruitment of metagenome assemblies in this study, which considers the count of genes on an assembly ($c$) and the ratio of the number of shared genes (designated as aforementioned) on an assembly to the total number of genes on that assembly ($r$). The following parameters were chosen for recruitment of metagenome assemblies in this study: $c\ = \ 3$ paired with $r\ = {\rm{\ }}0.5$. These parameters recruit assemblies containing at least three genes, including two shared genes.

Five mock metagenomic datasets were used to assess the performance of this strategy. Briefly, simulated reads of 60 bacterial genomes from 14 common genera (*Bifidobacterium, Clostridium, Enterobacter, Escherichia, Haemophilus, Klebsiella, Lactobacillus, Neisseria, Pseudomonas, Salmonella, Shigella, Staphylococcus, Streptococcus, Yersinia*) present in the human gut (Supplementary Table S1), including the 5 pathogenic *E. coli* strains mentioned above and 10 strains from 9 closely related *Enterobacteriaceae* species (*Enterobacter aerogenes, Enterobacter cloacae, Escherichia albertii, Escherichia fergusonii, Klebsiella oxytoca, Klebsiella pneumoniae, Shigella boydii, Shigella sonnei*, and *Salmonella enterica*), were generated by iMESSi [47]. Each dataset was simulated at the same complexity level with 100 million (M) 80-bp paired-end reads of 12 strains from 11–12 different genera, including 2 strains of closely related species to *E. coli*, and the relative abundances of strains were assigned by the broken-stick model (Supplementary Table S2). Simulated reads were first independently assembled into assemblies by SOAPdenovo2 in each dataset [44], with an empirical *k*-mer size of 41. Genes were then predicted on assemblies longer than 500 bp using MetaGeneMark [43] (default parameters were used except the minimum length of genes was set as 100 bp).

Assemblies of each mock dataset were first aligned against the five pathogenic *E. coli* reference genomes by BLAT [46]. Those assemblies that have an overall ≥90% overlap and ≥95% identity with the reference genomes were considered as *E. coli* genome derived (traditional genome alignment-based strategy). Those *E. coli* genome-derived assemblies containing at least three genes (i.e., containing at least two edges) were recruited for construction of a reference pangenome network (RPGN). A query pangenome network (QPGN) was then generated from assemblies selected using the gene alignment-based strategy with $c\ = \ 3$ and $r\ = {\rm{\ }}0.5$ as described above.

Accuracy of query assembly recruitment was assessed with respect to conformity and divergence between the RPGN with the QGPN (Supplementary Text 4 and Text 5 in Supplementary File S2). The result showed that the QPGN recovered 84.3% of node and 84.7% of edge in the RPGN, while falsely included 1.1% of node and 2.2% of edge, which demonstrated the high accuracy of the gene alignment-based strategy for recruitment of metagenome assemblies.

### Construction and visualization of the 760-metagenome pangenome network

Assemblies and representative genes of the 760 metagenomes generated in [24] were used here, since they were produced using the same methods and parameter settings used in this study. A pangenome network was generated following steps described above, again using *E. coli* K-12 as the reference and $c\ = {\rm{\ }}3$$r\ = {\rm{\ }}0.5$ for assembly recruitment. The resulting pangenome network was visualized in the same way that the 5-*E. coli*-genome pangenome network was visualized.

### Analysis of subnetworks comprising a pangenome network

A total of 10–700 metagenomes were randomly sampled from the above-mentioned 760 metagenomes. For each sub-dataset, a pangenome network was constructed after assembly recruitment using *E. coli* K-12 as the reference genome. For each pangenome network, reference-specific edges were removed before counting the number of subnetworks. Only sub-datasets with a size of recruited assemblies greater than 5 Mb were used to generate the scatterplot, in which a curve with 95% confidence intervals was fitted by the “loess” smoothing method in R [26].

### Computational resources and runtime

Timings for major steps of the MetaPGN pipeline are shown below. Tests were run on a single central processing unit (CPU) of an Intel Core Processor (Broadwell) with 64 GB of random access memory (RAM), not otherwise specified. The timings were CPU time including parsing input and writing outputs (h for hours, m for minutes, and s for seconds).

The average time for gene prediction for a mock metagenome was 7 s, and it varies depending on the size of the metagenome. The time for redundancy elimination of genes using CD-HIT [45] was 1 m 44 s for the five *E. coli* stains and 50 m 19 s for the five mock datasets. For the 760 metagenomes, to perform redundancy elimination in parallel, we divided all genes into 200 sections, which resulted in 20,101 [${\rm{N\ }} = ( {n + 1} )\ \times ( {n \div 2} ) + 1,\ n\ = \ 200$] clustering tasks, and then submitted each task to available machines in a high-performance computing cluster. The dividing step took 20 m 4 s, with a peak memory usage of 1 GB in the local machine; the average time for a clustering task was 44 m, taking less than 3 GB of RAM and consuming a total time of 14,814 h. The time for recognizing the status (reference specific, query specific, or shared) for nodes and edges was 10 s for the five *E. coli* strains, 1 m for the five mock datasets, and 24 m for the 760 metagenomes. Finally, the generation of the pangenome network took less than 1 s for the five *E. coli* strains, less than 1 s for the five mock datasets, and 3 m 35 s for the 760 metagenomes.

## Availability of source code and requirements

Project name: MetaPGN

Project home page: https://github.com/peng-ye/MetaPGN

Operating system(s): Platform independent

Programming language: Perl (version 5.0 or above)

Other requirements: MetaGeneMark (version 2.8 or above), Java (latest version), Cytoscape (version 3.0 or above)

License: GPLv3.0

RRID:SCR_016472

## Availability of supporting data

Genome sequence of 60 strains (including 5 *E. coli* strains) and the *E. coli* K-12 reference genome were downloaded from the National Center for Biotechnology Information (ftp://ftp.ncbi.nlm.nih.gov/genomes/refseq/bacteria/, please refer to Supplementary Table S1 for detailed information). Sequencing data for the 760 metagenomes were previously generated in the Metagenomics of the Human Intestinal Tract project [21–24], and assemblies of these 760 metagenomes are deposited at the European Nucleotide Archive (ENA) under PRJEB28245. The MetaPGN pipeline, related manuals, and Cytoscape session files for *E. coli* pangenome networks derived from five pathogenic *E. coli* strains and from 760 metagenomes are available in the MetaPGN project page in GitHub [48]. Additional data supporting this work are also available in the *GigaScience* database, GigaDB [49].

## Additional files

Supplementary Figure S1. Another cluster of genes containing MGEs, flanked by different shared genes on different *E. coli* genomes (manually arranged). Green, blue, red nodes and edges denote reference-specific, shared, and query- specific genes and gene adjacencies, respectively. Triangles represent MGEs. Size of nodes and thickness of edges indicates their weight (occurrence frequency). Numbers alongside shared genes are their indices in the representative gene set, and numbers in parentheses indicate loci of these genes in the reference genome.

Supplementary Figure S2. Examples of arrangement determined by the algorithm. (a) arrangements for shared nodes (blue) and reference-specific nodes (green). (b-e) arrangements for query-specific nodes (red).

Supplementary Table S1. Metadata of isolate genomes used in this study.

Supplementary Table S2. Statistics for the 5 mock metagenomic datasets.

Supplementary Table S3. Tables of nodes and edges in the 5-*E. coli*-genome pangenome network and the 760-metagenome pangenome network.

Supplementary File S1: Texts for, 1) steps for constructing pangenome networks, 2) steps for installing the plug-in and visualizing pangenome networks in Cytoscape.

Supplementary File S2: Texts for, 1) steps for selecting query-specific nodes for arrangement, 2) Comparison of the reference pangenome network (RPGN) and the query pangenome network (QPGN), and 3) detailed definitions of conformity and divergence for nodes and edges.

Supplementary File S3: “5-*E. coli*-genome pangenome network.pdf”, PDF file for *E. coli* pangenome network derived from five pathogenic *E. coli* strains.

Supplementary File S4: “760-metagenome pangenome network.pdf”, PDF file for *E. coli* pangenome network derived from 760 genuine metagenomes.

## Abbreviations

BLAST: Basic Local Alignment Search Tool; BLAT: Basic Local Alignment Search Tool (BLAST)-like alignment tool; CA: colanic acid; CPU: central processing unit; LPS: lipopolysaccharide; MGE: mobile genetic element; NORA: newly onset untreated rheumatoid arthritis; QPGN: query pangenome network; RAM: random access memory; RBS: ribosomal binding site; RPGN: reference pangenome network.

## Ethics approval

This study has been approved by the Institutional Review Board on Bioethics and Biosafety (reference BGI-IRB 16 017).

## Competing interests

The authors declare that they have no competing interests.

## Funding

This study was supported by the National Natural Science Foundation of China (31601073).

## Authors’ contributions

J.L. conceived and directed the project. S.T. developed the plug-in. S.T., X.C., Z.Z., and Y.P. developed other codes. Y.P., H.Z., J.L., D.W., S.T., and H.J. performed research. S.T. and Y.P. prepared display items. J.L., H.Z., Y.P., D.W., K.K., and S.T. participated in discussion of the project. Y.P., D.W., H.Z., and S.T. wrote the manuscript. All authors contributed to the revision of the manuscript.

## Supplementary Material

GIGA-D-18-00147_Original_Submission.pdf

GIGA-D-18-00147_Revision_1.pdf

GIGA-D-18-00147_Revision_2.pdf

Response_to_Reviewer_Comments_Original_Submission.pdf

Response_to_Reviewer_Comments_Revision_1.pdf

Reviewer_1_Report_(Original_Submission) -- Alexander Herbig 05/27/2018 Reviewed

Reviewer_1_Report_Revision_1 -- Alexander Herbig 08/27/2018 Reviewed

Reviewer_2_Report_(Original_Submission) -- Andre Mu 07/05/2018 Reviewed

Reviewer_2_Report_Revision_1 -- Andre Mu 08/22/2018 Reviewed

Supplemental Files
