# Supplementary material for: MetaPGN: a pipeline for construction and graphical visualization of annotated pangenome networks
Source: Gigascience. 2018 Oct 2;7(11):giy121. doi: 10.1093/gigascience/giy121 (PMC6251982; doi:10.1093/gigascience/giy121)
Supplement: GIGA-D-18-00147_Original_Submission.pdf [file giy121_giga-d-18-00147_original_submission.pdf]

GigaScience

Construction and visualization of annotated pangenome networks enable graphical analyses for prokaryotic genetic diversity

--Manuscript Draft--

Manuscript Number:	GIGA-D-18-00147
Full Title:	Construction and visualization of annotated pangenome networks enable graphical analyses for prokaryotic genetic diversity
Article Type:	Research
Funding Information:	National Natural Science Foundation of China (31601073) — Dr Junhua Li
Abstract:	Pangenome analyses facilitate the interpretation of genetic diversity and evolutionary history of a taxon. However, there is an urgent and unmet need to develop new tools for advanced pangenome construction and visualization, especially for metagenomic data. Here we present an integrated pipeline, named MetaPGN, for graphically construction and visualization of pangenome networks from microbial genomes or metagenomes. Given isolate genomes or metagenomic assemblies coupled with a reference genome of the targeted taxon, MetaPGN generates a pangenome in a topological network, consisting of genes (nodes) and gene-gene genomic adjacencies (edges) of which biological information can be easily updated and retrieved. MetaPGN also includes a self-developed Cytoscape plugin for layout of and interaction with the resulting pangenome network, providing an intuitive and interactive interface for fully exploration of genetic diversity. We demonstrate the utility of MetaPGN by constructing Escherichia coli (E. coli) pangenome networks from five E. coli pathogenic strains and 760 human gut microbiomes respectively, revealing extensive genetic diversity of E. coli within both isolates and gut microbial populations. With the ability to extract and visualize gene contents and gene-gene physical adjacencies of a specific taxon from large-scale metagenomic data, MetaPGN provides advantages in expanding pangenome analysis to uncultured microbial taxa. MetaPGN is available at https://github.com/peng-ye/MetaPGN.
Corresponding Author:	Junhua Li, Ph.D.<br>BGI<br>shenzhen, guangdong CHINA
Corresponding Author Secondary Information:	
Corresponding Author's Institution:	BGI
Corresponding Author's Secondary Institution:	
First Author:	Ye Peng
First Author Secondary Information:	
Order of Authors:	Ye Peng
	Shanmei Tang
	Dan Wang
	Huanzi Zhong
	Huijue Jia
	Xianghang Cai
	Zhaoxi Zhang
	Minfeng Xiao
	Huanming Yang

Powered by Editorial Manager® and ProduXion Manager® from Aries Systems Corporation

	Jian Wang
	Xun Xu
	Junhua Li, Ph.D.
**Order of Authors Secondary Information:**	
**Additional Information:**	
**Question**	**Response**
Are you submitting this manuscript to a special series or article collection?	No
**Experimental design and statistics**<br><br>Full details of the experimental design and statistical methods used should be given in the Methods section, as detailed in our Minimum Standards Reporting Checklist. Information essential to interpreting the data presented should be made available in the figure legends.<br><br>Have you included all the information requested in your manuscript?	Yes
**Resources**<br><br>A description of all resources used, including antibodies, cell lines, animals and software tools, with enough information to allow them to be uniquely identified, should be included in the Methods section. Authors are strongly encouraged to cite Research Resource Identifiers (RRIDs) for antibodies, model organisms and tools, where possible.<br><br>Have you included the information requested as detailed in our Minimum Standards Reporting Checklist?	Yes
**Availability of data and materials**<br><br>All datasets and code on which the conclusions of the paper rely must be either included in your submission or deposited in publicly available repositories (where available and ethically appropriate), referencing such data using a unique identifier in the references and in the "Availability of Data and Materials" section of your manuscript.<br><br>Have you have met the above requirement as detailed in our Minimum	No

Standards Reporting Checklist?	
If not, please give reasons for any omissions below.<br><br>as follow-up to "**Availability of data and materials**<br><br>All datasets and code on which the conclusions of the paper rely must be either included in your submission or deposited in publicly available repositories (where available and ethically appropriate), referencing such data using a unique identifier in the references and in the “Availability of Data and Materials” section of your manuscript.<br><br>Have you have met the above requirement as detailed in our Minimum Standards Reporting Checklist?<br><br>"	We are uploading assembled contigs of 760 metagenomes used in this study to EBI and CNSA, and will provide accession numbers when it is done.

Powered by Editorial Manager® and ProduXion Manager® from Aries Systems Corporation

Manuscript
Click here to download Manuscript MetaPGN-Manuscript-V1.docx

Construction and visualization of annotated pangenome networks enable graphical analyses for prokaryotic genetic diversity

Abstract

Pangenome analyses facilitate the interpretation of genetic diversity and evolutionary history of a taxon. However, there is an urgent and unmet need to develop new tools for advanced pangenome construction and visualization, especially for metagenomic data. Here we present an integrated pipeline, named MetaPGN, for graphically construction and visualization of pangenome networks from microbial genomes or metagenomes. Given isolate genomes or metagenomic assemblies coupled with a reference genome of the targeted taxon, MetaPGN generates a pangenome in a topological network, consisting of genes (nodes) and gene-gene genomic adjacencies (edges) of which biological information can be easily updated and retrieved. MetaPGN also includes a self-developed Cytoscape plugin for layout of and interaction with the resulting pangenome network, providing an intuitive and interactive interface for fully exploration of genetic diversity. We demonstrate the utility of MetaPGN by constructing *Escherichia coli* (*E. coli*) pangenome networks from five *E. coli* pathogenic strains and 760 human gut microbiomes respectively, revealing extensive genetic diversity of *E. coli* within both isolates and gut microbial populations. With the ability to extract and visualize gene contents and gene-gene physical adjacencies of a specific taxon from large-scale metagenomic data, MetaPGN provides advantages in expanding pangenome analysis to uncultured microbial taxa. MetaPGN is available at https://github.com/peng-ye/MetaPGN.

Keywords: pangenome, visualization, metagenomics

Introduction

The concept of pangenome, defined as the full complement of genes in a clade, was first introduced by Tettelin *et al.* in 2005 [1]. Pangenome analyses of a species now provide insights into core- and accessory-genome profiles, within-species genetic diversity, evolutionary dynamics and niche-specific adaptions. Hitherto, plenty of tools have been proposed for pangenome analysis on genomic or metagenomic data (Table 1).

Typical pangenome tools such as GET_HOMOLOGUES [2] and PGAP [3], mainly focus on analyzing the homologous gene families and calculating the core/accessory genes of a given taxon. However, these tools cannot provide the variations of gene-gene physical relationships. Tools like GenoSets [4], PGAT [5], PEGR [6], EDGAR [7], GenomeRing [8] and PanViz [9] are developed to generate a linear or circular presentation of homologous sequences/genes, which can indicate the physical relationships between genomic sequences or genes. However, these tools visualize the core/accessory sequences or genes on independent lines and thus cannot intuitively present the complex variations among the input genomes.

Pangenomes built using *de Bruijn* graph, like SplitMEM [10] and a tool introduced by Baier *et al.* [11], partly solve the above problems. In the resulting graph yielded by these tools, complete pan-genome is represented in a compact graphical representation such that the core/accessory status of any genomic sequences is immediately identifiable, along with the context of the flanking sequences. This strategy enables powerful topological analysis of the pan-genome not possible from a linear/circular representation. Nevertheless, tools based on the *de Bruijn* graph algorithm can only construct a compact network comprised of core/accessory genomic sequences instead of genes, which means retrieving or updating functional information in downstream analysis will be difficult. Furthermore, these tools do not visualize the constructed *de Bruijn* graph and provide an interactive interface for users to explore the graph.

Moreover, all the above-mentioned tools analyzed pangenomes via genomic data which require organisms isolated from the environment and cultured *in vitro*. Recent advances in metagenomics have led to a paradigm shift in pangenome study from a limited quantity of cultured microbial genomes to large-scale metagenomic datasets containing huge potential of functional and phylogenetic resolution from the nonculturable taxa. Several existing tools dealing with

metagenomic data are based on constructed pangenomes, namely; cannot utilize the abundant gene resources contained in metagenomes to extend the pangenomes in question. For example, PanPhlAn [12], MIDAS [13], and a pipeline introduced by Delmont and Eren [14] map reads onto a reference pangenome, to describe the pattern of presence/absence of genes in metagenomes. For another instance, Kim *et al.* [15] clustered genes predicted from metagenomic contigs with Bacillus core genes for profiling the Bacillus species in the microbiomes. Recently, Farag *et al.* [16] aligned metagenome contigs with reference genomes for identification of *"Latescibacteria"* genomic fragments. Though this strategy can theoretically recruit sequences not present in the reference genomes, it is likely to filter out *"Latescibacteria"* genomic fragments with structural variations compared to the reference ones. Furthermore, all these aforementioned methods for metagenomic data did not organize the pangenome using a network, which is essential for efficiently storage and visualization of pangenome constructed from metagenomic data.

Here, we introduce an integrated pipeline (MetaPGN) for network-based construction and visualization of prokaryotic pangenomes for both bacterial genomes and metagenomes. Given genomic or metagenomic assemblies and a reference genome of a taxon of interest, MetaPGN derives a pangenome network for integrating genes (nodes) and gene-gene adjacencies (edges) belonging to this taxon. MetaPGN also includes a specific Cytoscape plugin for layout of and interaction with the resulting pangenome network, providing an intuitive and interactive interface for the exploration of gene diversity. For example, in the visualized network in Cytoscape, users can specify gene annotations, customize the appearance of nodes and edges, search and concentrate on genes of certain functions. We applied MetaPGN on assemblies from five pathogenic *E. coli* strains and 760 human gut microbiomes respectively, with *E. coli* K-12 substr. MG1655 (*E. coli* K-12) being the reference genome. It showed that by taking gene adjacency into account and visualizing the pangenome network in a well-organized manner, MetaPGN can assist in observing genetic diversity in genomic or metagenomic assemblies graphically and conveniently.

Results

General workflow. MetaPGN accepts genome or metagenome assemblies as input (query assemblies) and requires a reference genome for recruitment of the query assemblies and as the skeleton of the pangenome network. The MetaPGN pipeline can be divided into two main parts: (i) construction of a pangenome network comprised of representative genes, including gene prediction, gene redundancy elimination, gene type determination, pairwise gene adjacency extraction, assembly recruitment (for metagenomic assemblies) and pangenome network generation and, (ii) visualization of the pangenome network in an organized way, where nodes represent genes and edges indicate gene adjacencies, in Cytoscape [17] with a self-developed plugin (Fig. 1, Fig. S1, and Methods). From the resultant pangenome network, the degree of similarity among homologous genes, as well as their genomic context is easily visible. Also of note is that users can add and update annotation for nodes and edges in the networks, based on which elements of interest can be accessed conveniently.

Pangenome network of 5 pathogenic *Escherichia coli* genomes. In order to demonstrate its potential in studying microbial genetic diversity and phenotype-genotype relationship, we first applied MetaPGN on genomes of 5 pathogenic *E. coli* isolates, *E. coli* O26:H11 str. 11368, *E. coli* O127:H6 E2348/69, *E. coli* O157:H7 str. EDL933, *E. coli* O104:H4 str. 2011C-3493 and *E. coli* 55989. A commensal *E. coli* strain, K-12 substr. MG1655 (Supplementary Table S1) was chosen as the reference genome in this instance and in all examples shown below.

A pangenome network consisting of 9,161 nodes and 11,788 edges (Supplementary Table S3, Supplementary File 2) was constructed and visualized (Methods). Based on the well visualized pangenome network along with functional annotation, we can now graphically observe the extent of variations of certain genes, as well as their genomic context. For example, when focusing on a cluster of flagellar genes (Fig. 2a), we found that *fliC* genes coding the filament structural protein (H-antigen) and *fliD* gene coding the filament capping protein are highly variable among these *E. coli* strains. In contrast, four genes coding chaperones (*fliS*, *fliT*, *fliY*, *fliZ*) and a gene (*fliA*) relating to the regulation of the expression of flagellar components are conserved over all the *E. coli* strains investigated. A gene (270 bp) coding hypothetical protein is uniquely presented between *fliC* and *fliD* in *E. coli* O157:H7 str. EDL933.

In a fimbria protein-related gene cluster, compared to the reference *E. coli* strain, all the 5 pathogenic strains possess several genes located between two conserved genes coding an outer membrane protein and a regulatory protein, and *E. coli* O127:H6 E2348/69 uniquely exhibits more genes encoding proteins of unknown functions (Fig. 2b).

For a gene cluster responsible for the biosynthesis of lipopolysaccharides, *E. coli* O127:H6 E2348/69 shares three genes with reference strain, that differentiate from the other 4 pathogenic strains (Fig. 2c). For another gene cluster of related function, the *E. coli* O127:H6 E2348/69 also shows a strain-specific duplication event of two colonic acid (CA)-related genes (wcaH and wcaG, denoted by a purple dash line in Fig. 2d). It has been demonstrated that CA can modify lipopolysaccharide (LPS) into a novel form (M_{LPS}) which may enhance survival of *E. coli* in different putative ways [18]. The two wcaH genes in *E. coli* O127:H6 E2348/69, though share high similarity (99.1% identity), may confer the strain with diverse functional potentials for CA formation and thereby the novel survival mechanism yet unknown.

In addition, the German outbreak *E. coli* O104:H4 str. 2011C-3493 comprises identical nodes and edges in the flagellar-related gene cluster (Fig. 2a) and the O antigen-related gene cluster with a historical *E. coli* 55989 (Fig. 2d), suggesting the close evolutionary relationship between these strains as previously reported [19,20].

These results demonstrate the feasibility of MetaPGN for construction and visualization of microbial pangenomes in an organized way. Moreover, by involving genomic adjacency and offering easy-to-achieve biological information, MetaPGN provides a convenient way to assist biologists in exposing genetic diversity for genes of interest among the organisms under study.

Pangenome network of *E. coli* in 760 metagenomes. Moving beyond surveying the pangenome network of isolate genomes, we applied MetaPGN in metagenomic datasets to interrogate the *E. coli* pangenome network on a grander scale. Assemblies of 760 metagenomes sequenced in the Metagenomics of the Human Intestinal Tract (MetaHIT) project [21–24] were collected, which contained 8,096,991 non-redundant genes with annotations [24]. As metagenome assemblies are from varied taxa, it is necessary to recruit assemblies of the targeted taxon before construction of the pangenome network. In this study, metagenome assemblies were recruited using a gene alignment-based strategy, which was assessed with mock datasets (Methods). With the recruited

assemblies, a pangenome network consisting of 9,406 nodes and 14,676 edges (Supplementary Table S3, Supplementary File S3) was generated and visualized after refinement (Methods).

Based on annotation, we first searched flagellin-related genes in this network. We found that the pattern of adjacencies among these genes was similar to that in the pangenome network of the 5 pathogenic *E. coli* genomes: *fliC* and *fliD* are hypervariable while *fliT*, *fliY*, *fliZ* and *fliA* are very conserved among these 760 samples. However, some genes of unknown function locate between *fliC* and *fliA* (Fig. 3a), instead of between *fliC* and *fliD* in the pangenome network of the 5 pathogenic *E. coli* strains (Fig 2a).

We then investigated mobile genetic elements (MGEs) in this pangenome network, as they can induce various types of genomic rearrangements[25]. Of the 362 nodes (~4%) annotated as MGE-related (according to Cluster of Orthologous Groups annotation done in reference 28), many were flanked by different shared genes on different *E. coli* genomes. In a region of the network, a gene cluster containing MGEs is query-specific, indicating there might be genomic rearrangements caused by strain-specific MGEs within the *E. coli* species (Fig. 3b). In another part of the network harboring MGEs, we observed that several branches of non-MGE genes are inserted in between two MGEs, which may imply a mutation hotspot within the region, or the existence of MGEs as yet undescribed (Fig. S1).

Application of MetaPGN in large-scale metagenomic data generated an *E. coli* pangenome network that might hardly be constructed from isolated genomes. As demonstrated here, the assembly-recruitment based, well-organized and visualized pangenome network can greatly expand our understanding in the genetic diversity of a taxon, although further efforts in bioinformatic and experimental analyses are needed to verify and extend these findings.

Assessment of pangenome networks derived from metagenomes. Genomic sequences assembled from metagenomic data are usually fragmentary resulted from limitations in sequencing platforms and bioinformatic algorithms. Due to this nature, a pangenome network recovered from a limited number of assemblies is likely to be segmented compared to a complete genome. To propose a minimum size of assemblies for getting a relatively connected pangenome network, we assessed the completeness of *E. coli* pangenome networks derived from varying size of recruited assemblies (Methods). As shown in Fig. 4, the count of connected subnetworks drops dramatically

with the total length of recruited assemblies increasing from 5 Mb to 50 Mb (roughly from 1 × to 10 × of a *E. coli* genome), then barely changes even when using all recruited assemblies of the dataset (215 Mb, from 760 samples). Based on this analysis, a minimum size of recruited assemblies 10-fold of the studied genome is required to generate a relatively intact pangenome network when constructed from metagenomes.

Discussion

Since first coined more than a decade ago, pangenome analysis has provided a framework for studying the genomic diversity within a species. Current methods for pangenome analyses mainly focus on gene contents but ignore their genomic context, as well as have shortages in pangenome visualization. Besides, available methods are usually designed for genomic data and not capable of constructing pangenomes from metagenomics data. To fill these gaps, our MetaPGN pipeline takes genome or metagenome assemblies as input, uses gene contents as well as pairwise gene adjacency to generate a compact graphical representation for the gene network based on a reference genome, and visualizes the network in Cytoscape with a self-developed plugin (Fig. 1, Fig. S2).

From the two MetaPGN-derived *E. coli* pangenome networks, we can directly observe the diversity of genes among the five pathogenic *E. coli* strains and 760 human gut microbiomes with respect to the reference genome. For instance, in the pangenome network for the 5 pathogenic *E. coli* strains, we found that gene *fliC* which carries H-antigen specificity were highly variable among the *E. coli* assemblies (Fig. 2a). This *fliC* gene was more varied in the 760 human gut microbiomes (Fig. 3a). In addition, genes for synthesis of O-antigen and outer membrane protein showed a great diversity in the pangenome network of the 5 E. coli strains (Fig. 2c, Fig. 2d). These results are in agreement with previous findings on H-antigen specificity related genes [26–28] and O-antigen related genes [29,30]. We also showed that when gene adjacency is incorporated into the construction and visualization of pangenomes, locations of genes of unknown function are identified, which may be helpful for the inference of their biological functions. For example, in both the two pangenome networks, we found genes of unknown function locating between the fliC gene and other flagellin-related genes (Fig. 2a, located between *fliC* and *fliD*, Fig. 3a, located between *fliC* and *fliA*), indicating that these function-unknown genes may play a role in flagellin biosynthesis [31], although further experimental trials are needed to prove this point. Additionally,

from the pangenome network of the five *E. coli* strains, we observed a great variation event on *E. coli* O127:H6 E2348/69, which was further verified to stem from a duplication event of two colonic acid (CA)-related genes (wcaH and wcaG, Fig 2d). This finding indicates that knowledge of genomic adjacency may also shed light on further studying structural variations among the input assemblies. If extended, genomic adjacency may help in finding possible functional sequences which potentially drive variations such as genomic repeats discussed by Delihas [32] and Wang *et al.* [33], and lead to the discovery of new functional modules as illustrated by Doron *et al.* [34]. These examples show that visualizing a pangenome involving gene adjacencies, and providing easy-to-add and retrieve biological information, is of significance.

Although examples shown in this study use the genome of a commensal *E. coli* strain for assembly recruitment and network arrangement, users can specify the reference genome when applying MetaPGN. Epidemiologists can use MetaPGN to compare assemblies of outbreak strains or viruses, such as *Vibrio cholerae* or Ebola virus, with those of some well-studied pathogenic strains to find novel variations involved in pathogenesis, which may further provide candidate targets for drug and vaccine design [35,36]. Moreover, pangenome networks built from metagenomes of different cohorts may help us better observe and compare the diversity of a species among these cohorts, and may thus give a hint to explain the inconsistent findings of certain disease-related gut microbes. For instance, Fredrik *et al.* reported that *Prevotella copri* (*P. copri*) improved hosts' glucose metabolism [37] while Oluf *et al.* argued that *P. copri* induced insulin resistance [38]. MetaPGN can therefore be used to build a pangenome network of these disease-related microbes, and study the genetic variations which might explain these inconsistent findings.

It should be noticed that, in this pipeline, we compare genes depending on nucleotide-level sequence identity and overlap: genes with ≥95% identity and ≥90% overlap are regarded as the same gene. However, as we know, genes of the same function may not satisfy this criterion (≥95% identity and ≥90% overlap), whereas protein encoded by these genes can be less diverse. Hence, in our future work, we can cluster genes by comparing their nucleotide sequences as well as the amino acid sequences. Besides, the current MetaPGN pipeline does not consider other genomic features, nor physical distances between genes in constructing the pangenome network. However, differences in other genomic features such as ribosomal binding site (RBS) sequences [39,40] and distances between the RBS and start codons [41] may result in distinct phenotypes. Therefore, users can involve these information in analyzing pangenome networks as well.

To conclude, MetaPGN would serve to directly observe genetic diversity in pangenome networks, better understand genotype-phenotype relationships and the evolutionary history.

Methods

Pangenome network construction in MetaPGN. First, gene prediction of query assemblies is performed using MetaGeneMark (Version 2.8) [42].In order to eliminate redundancy, the resultant genes are clustered by CD-HIT (Version 4.5.7) [43] with identity ≥95% and overlap ≥90, and genes in a same cluster are represented by the longest sequence of the cluster which is called the representative gene. Representative genes of all clusters are subsequently aligned against genes on the given reference genome using BLAT (Version 34) [44]. From the alignment result, genes shared between the representative gene set and the reference gene set with identity ≥95% and overlap ≥90% are defined as ‘shared genes’. The remaining representative and reference genes other than those shared genes are defined as ‘query-specific genes’ and ‘reference-specific genes’, respectively. Pairwise gene physical adjacency of representative genes on the query assemblies and of reference genes are then extracted, and status for each adjacency of being ‘shared’, ‘query-specific’, or ‘reference-specific’ is determined. Finally, based on the recruited assemblies and the reference genome, an initial pangenome network is generated: each node stands for a reference gene or a representative gene on the recruited assemblies; two nodes are connected by an edge if they are physically adjacent on the recruited assemblies or on the reference genome. The weight of a node or an edge denotes its occurrence frequency on all of the recruited assemblies and the reference genome.

Pangenome network visualization in MetaPGN. The following preprocessing work on the initial pangenome network was implemented before visualization: 1. The initial pangenome network was refined by removing isolated networks (networks not connected with the backbone) and tips (nodes only connected with another node); 2. Nodes and edges were added with some extra attributes, such as the status of the nodes and edges (query-specific, reference-specific or shared), whether the genes for the nodes were phage-, plasmid-, CRISPR- related genes and so on (Supplementary Table S3). Users can specify the attributes of nodes and edges according to their own datasets.

We then used a self-developed Cytoscape plugin to visualize the pangenome network in an organized way (Supplementary Text 1 in Supplementary File S1 illustrates how to install the plugin in Cytoscape). Our algorithm for organizing nodes in the network is as follows:

1. Construct a circular skeleton for the pangenome network with shared nodes and reference-specific nodes, according to positions of their related reference genes on the reference genome. If there are two or more representative genes similar to the same reference gene (≥95% identity and ≥90% overlap), use one of these representative genes to construct the skeleton and place the others on both sides of the skeleton in turn (Fig. S2 a).
2. Arrange query-specific nodes region by region, including,
   2.1. Select query-specific nodes in a region spanning less than 30 nodes in the skeleton (see Supplementary Text 2 in Supplementary File S1 for more details).
   2.2. Arrange these query-specific nodes as follows,
      i. For those that directly link with two nodes on the skeleton, place them on the bisector of the two skeleton nodes. If there are two or more query-specific nodes directly linking with the same pair of nodes on the skeleton, place them on both sides of the bisector of these pair of skeleton nodes in turn (Fig. S2 b).
      ii. Among the remaining nodes, for those that directly link with two placed nodes, place them on the bisectors of the placed ones. Iterate this step for five times (Fig. S2 c).
      iii. For the remaining nodes, place them into an arc without moving the placed nodes (Fig. S2 d), or else place them one by one starting near a placed node (Fig. S2 e).

Construction and visualization of the 5-*E. coli*-genome pangenome network. Genes were extracted from the reference genome for each strain (Supplementary Table S1). Using *E. coli* K-12 as the reference, a pangenome network was generated following Step 2-6 described above. In the visualization of this pangenome network, we used green, blue and red color to denote a reference-specific, shared, and query-specific node or edge, respectively, and specified sizes of nodes and widths of edges with their occurrence frequency in the input genomes.

Assessment of the gene alignment-based assembly recruitment strategy. A gene alignment-based strategy was used for recruitment of metagenome assemblies in this study, which considers

1) the count of genes on an assembly (c), and 2) the ratio of the number of shared genes (designated as aforementioned) on an assembly to the total number of genes on that assembly (r). $c = 3$ paired with $r = 0.5$, requiring at least 3 genes including 2 shared genes containing in an assembly, was chosen for recruitment of metagenome assemblies in this study.

5 mock metagenomic datasets were used to assess the performance of this strategy. Briefly, simulated reads of 50 bacterial genomes from 10 common genera present in the human gut were generated by iMESSi [45], including the 5 pathogenic *E. coli* strains mentioned above (Supplementary Table S1). Each dataset was simulated at the same complexity level with 80 million (M) 80bp paired-end reads of 10 strains from 10 different genera and the relative abundances of strains were assigned by the broken-stick model (Supplementary Table S2). Simulated reads were first independently assembled into assemblies by SOAPdenovo2 in each dataset [43], with an empirical k-mer size of 41. Genes were then predicted on assemblies longer than 500 bp using MetaGeneMark [40] (default parameters were used except the minimum length of genes was set as 100 bp).

Assemblies of each mock dataset were first aligned against the 5 pathogenic *E. coli* reference genomes by BLAT[44]. Those assemblies that have an overall ≥90% overlap and ≥95% identity with the reference genomes were considered as *E. coli* genome-derived (traditional genome alignment-based strategy) and were recruited for construction of a reference pangenome network (RPGN). A query pangenome network (QPGN) was then generated from assemblies selected by the gene alignment-based strategy with $c = 3$ and $r = 0.5$ as described above.

Accuracy of query assembly recruitment was assessed, in respect of conformity and divergence between the RPGN with the QGPN (Supplementary Text 3 and 4 in Supplementary File S1). The result showed that the QPGN recovered 87.9% of node and 90.3% of edge in the RPGN, while falsely included 0% of node and less than 1% of edge, which demonstrated the high accuracy of the gene alignment-based strategy for recruitment of metagenome assemblies.

Construction and visualization of the 760-metagenome pangenome network. Assemblies and representative genes of the 760 metagenomes generated in Reference [24] were used here, since they were produced using identical methods and parameter settings in this study. A pangenome network was generated following steps described above, again using *E. coli* K-12 as the reference,

and $c = 3$, $r = 0.5$ for assembly recruitment. The resulting pangenome network was visualized in the same way as visualizing the 5-*E. coli*-genome pangenome network.

Analysis of subnetworks comprising a pangenome network. 10-700 metagenomes were randomly sampled from the above-mentioned 760 metagenomes. For each sub-dataset, a pangenome network was constructed after assembly recruitment using *E. coli* K-12 as the reference genome. For each pangenome network, reference-specific edges were removed before counting the number of subnetworks. Only sub-datasets with a size of recruited assemblies greater than 5 Mb were used to generate the scatterplot, in which a curve with 95% confidential intervals was fitted by the 'loess' smoothing method in R [46].

Data availability. Genome sequence of 50 strains (contained 5 *E. coli* genomes) and the reference genome were downloaded from the National Center for Biotechnology Information (ftp://ftp.ncbi.nlm.nih.gov/genomes/refseq/bacteria/). Please refer to Supplementary Table S1 for detailed information. Raw sequences of the 5 mock metagenomics datasets are available in the EBI repository /to/be/uploaded. The MetaPGN pipeline, and Cytoscape session files for *E. coli* pangenome networks derived from five pathogenic *E. coli* strains and from 760 metagenomes are available on Github (https://github.com/peng-ye/MetaPGN).

List of Figures

Figure 1. An Overview of the MetaPGN pipeline: from assemblies to a pangenome network. Gene prediction is performed on query assemblies. The resulting genes are clustered, after which genes in the same cluster are represented by the longest sequence of this cluster called the representative gene (node a-g). All these representative genes are then aligned against genes on the given reference genome. From the alignment result, genes shared between the representative gene set and the reference gene set are defined as 'shared' genes (blue). The remaining representative and reference genes other than those shared genes are defined as 'query-specific' genes (red) and 'reference-specific' genes (green), respectively. Pairwise gene physical adjacency of

representative genes on the query assemblies and of reference genes are then extracted, and status for each adjacency of being 'shared' (blue), 'query-specific' (red), or 'reference-specific' (green) is determined. Finally, based on the recruited assemblies and the reference genome, a pangenome network is generated: each node stands for a reference gene or a representative gene on the recruited assemblies; two nodes are connected by an edge if they are physically adjacent on the recruited assemblies or the reference genome. The weight of a node or an edge is its occurrence frequency on all of the recruited assemblies and the reference genome (Methods). The pangenome network is then visualized in Cytoscape with a self-developed plugin (Methods) for a better arrangement. Biological information of nodes and edges, such as gene name and annotation, can be easily retrieved in the interactive user interface in Cytoscape.

Figure 2. Subgraphs of highly variable genes in the pangenome network of 5 pathogenic *E. coli* strains (manually arranged). (a) a cluster of flagellar genes. (b) a cluster containing outer membrane protein-coding genes. (c) a cluster of genes responsible for biosynthesis of the O antigen. (d) another cluster of O antigen-related genes. Green, blue, red nodes and edges denote reference-specific, shared, and query- specific genes and gene adjacencies, respectively. Size of nodes and thickness of edges indicates their weight (occurrence frequency). Numbers alongside shared genes are their indexes in the representative gene set.

Figure 3. Two subgraphs of the pangenome network of *E. coli* constructed from 760 metagenomes (manually arranged). (a) a cluster of flagellar genes. (b) a cluster of genes containing MGEs. Green, blue, red nodes and edges denote reference-specific, shared, and query- specific genes and gene adjacencies. Triangles represent MGEs. Size of nodes and thickness of edges indicates their weight (occurrence frequency). Numbers alongside shared genes are their indexes in the representative gene set.

Figure 4. Number of subnetworks in pangenome networks derived from varying sizes of recruited assemblies. The x-axis indicates total length of recruited assemblies for each sub-dataset and the y-axis represents the number of subnetworks in the pangenome network derived from each sub-dataset. The curve was fitted for the scatters using the 'loess' smoothing method in R[46]. The shaded area displays the 95% confidential intervals of the curve. Axises are log2-transformed.

Additional information

Supplementary Figure S1. Another cluster of genes containing MGEs, flanked by different shared genes on different *E.coli* genomes (manually arranged). Green, blue, red nodes and edges denote reference-specific, shared, and query- specific genes and gene adjacencies. Triangles represent MGEs. Size of nodes and thickness of edges indicates their weight (occurrence frequency). Numbers alongside shared genes are their indexes in the representative gene set, and numbers in parentheses indicate loci of these genes in the reference genome.

Supplementary Figure S2. Examples of arrangement determined by the algorithm. (a) arrangements for shared nodes (blue) and reference-specific nodes (green). (b-e) arrangements for query-specific nodes (red).

Supplementary Table S1. Metadata of isolate genomes used in this study.

Supplementary Table S2. Statistics for the 5 mock metagenomic datasets.

Supplementary Table S3. Tables of nodes and edges in the 5-*E. coli*-genome pangenome network and the 760-metagenome pangenome network.

Supplementary File S1: Texts for, 1) steps for installing the plug-in and visualizing pangenome networks in Cytoscape, 2) steps for selecting query-specific nodes for arrangement, 3) Comparison of the reference pangenome network (RPGN) and the query pangenome network (RPGN), 4) detailed definitions of conformity and divergence for nodes and edges, and 5) supplementary references.

Supplementary File S2: "5-*E. coli*-genome pangenome network.pdf", PDF file for *E. coli* pangenome network derived from five pathogenic *E.coli* strains.

Supplementary File S3: "760-metagenome pangenome network.pdf", PDF file for *E. coli* pangenome network derived from 760 genuine metagenomes.

Abbreviations

CA: colonic acid; *E. coli*: *Escherichia coli*; LPS: lipopolysaccharide; MGEs: mobile genetic elements; *P. copri*: *Prevotella copri*.

Ethics approval

This study has been approved by the Institutional Review Board on Bioethics and Biosafety (reference number: BGI-IRB 16017).

Consent for publication

Not applicable.

Competing interests

The authors declare no competing interests.

Acknowledgements

This study was supported by the National Natural Science Foundation of China (No.31601073).

References

1. Tettelin H, Masignani V, Cieslewicz MJ, Donati C, Medini D, Ward NL, et al. Genome analysis of multiple pathogenic isolates of Streptococcus agalactiae: Implications for the microbial “pan-genome.” Proc Natl Acad Sci [Internet]. 2005;102:13950–5. Available from: http://www.pnas.org/cgi/doi/10.1073/pnas.0506758102

2. Contreras-Moreira B, Vinuesa P. GET_HOMOLOGUES, a versatile software package for scalable and robust microbial pangenome analysis. Appl Environ Microbiol. 2013;79:7696–701.

3. Zhao Y, Wu J, Yang J, Sun S, Xiao J, Yu J. PGAP: Pan-genomes analysis pipeline. Bioinformatics. 2012;28:416–8.

4. Cain AA, Kosara R, Gibas CJ. GenoSets: Visual Analytic Methods for Comparative Genomics. PLoS One. 2012;7.

5. Brittnacher MJ, Fong C, Hayden HS, Jacobs MA, Radey M, Rohmer L. PGAT: A multistrain analysis resource for microbial genomes. Bioinformatics. 2011;27:2429–30.

6. Fremez R, Faraut T, Fichant G, Gouzy J, Quentin Y. Phylogenetic exploration of bacterial genomic rearrangements. Bioinformatics. 2007;23:1172–4.

7. Blom J, Kreis J, Spänig S, Juhre T, Bertelli C, Ernst C, et al. EDGAR 2.0: an enhanced software platform for comparative gene content analyses. Nucleic Acids Res. 2016;44:W22–8.

8. Herbig A, Jäger G, Battke F, Nieselt K. GenomeRing: Alignment visualization based on SuperGenome

coordinates. Bioinformatics. 2012;28:7–15.

9. Pedersen TL, Nookaew I, Wayne Ussery D, Månsson M. PanViz: interactive visualization of the structure of functionally annotated pangenomes. Bioinformatics [Internet]. 2017;33:btw761. Available from: https://academic.oup.com/bioinformatics/article-lookup/doi/10.1093/bioinformatics/btw761

10. Marcus S, Lee H, Schatz M, Schatz MC. SplitMEM : Graphical pan-genome analysis with suffix skips BIOINFORMATICS SplitMEM : Graphical pan-genome analysis with suffix skips. bioArxive. 2014;0–7.

11. Baier U, Beller T, Ohlebusch E. Graphical pan-genome analysis with compressed suffix trees and the Burrows-Wheeler transform. Bioinformatics. 2015;32:497–504.

12. Scholz M, Ward D V, Pasolli E, Tolio T, Zolfo M, Asnicar F, et al. Strain-level microbial epidemiology and population genomics from shotgun metagenomics. Nat Methods [Internet]. Nature Publishing Group; 2016; Available from: http://www.nature.com/doifinder/10.1038/nmeth.3802

13. Nayfach S, Rodriguez-Mueller B, Garud N, Pollard KS. An integrated metagenomics pipeline for strain profiling reveals novel patterns of bacterial transmission and biogeography. Genome Res. 2016;26:1612–25.

14. Delmont TO, Eren AM. Linking pangenomes and metagenomes: the *Prochlorococcus* metapangenome. PeerJ [Internet]. 2018;6:e4320. Available from: https://peerj.com/articles/4320

15. Kim Y, Koh I, Young Lim M, Chung WH, Rho M. Pan-genome analysis of Bacillus for microbiome profiling. Sci Rep. 2017;7:1–9.

16. Farag IF, Youssef NH, Elshahed MS. Global distribution patterns and pangenomic diversity of the candidate phylum "Latescibacteria" (WS3). Appl Environ Microbiol. 2017;83:1–21.

17. Cytoscape: An Open Source Platform for Complex Network Analysis and Visualization [Internet]. [cited 2017 Nov 8]. Available from: http://www.cytoscape.org/

18. Meredith TC, Mamat U, Kaczynski Z, Lindner B, Holst O, Woodard RW. Modification of lipopolysaccharide with colanic acid (M-antigen) repeats in Escherichia coli. J Biol Chem. 2007;282:7790–8.

19. Guy L, Jernberg C, Arvén Norling J, Ivarsson S, Hedenström I, Melefors Ö, et al. Adaptive Mutations and Replacements of Virulence Traits in the Escherichia coli O104:H4 Outbreak Population. PLoS One. 2013;8.

20. Rasko DA, Webster DR, Sahl JW, Bashir A, Boisen N, Scheutz F, et al. Origins of the *E. coli* Strain Causing an Outbreak of Hemolytic–Uremic Syndrome in Germany. N Engl J Med [Internet]. 2011;365:709–17. Available from: http://www.nejm.org/doi/abs/10.1056/NEJMoa1106920

21. Qin J, Li R, Raes J, Arumugam M, Burgdorf KS, Manichanh C, et al. A human gut microbial gene catalogue established by metagenomic sequencing. Nature. Macmillan Publishers Limited. All rights reserved; 2010;464:59–65.

22. Le Chatelier E, Nielsen T, Qin J, Prifti E, Hildebrand F, Falony G, et al. Richness of human gut microbiome correlates with metabolic markers. Nature. 2013;500:541–6.

23. Nielsen HB. Identification and assembly of genomes and genetic elements in complex metagenomic samples without using reference genomes. nbt. 2014;2014:41–5.

24. Li J, Jia H, Cai X, Zhong H, Feng Q, Sunagawa S, et al. An integrated catalog of reference genes in the human gut microbiome. Nat Biotech [Internet]. 2014;advance on:834–41. Available from:

http://dx.doi.org/10.1038/nbt.2942%5Cn10.1038/nbt.2942%5Cnhttp://www.nature.com/nbt/journal/vaop/ncurrent/abs/nbt.2942.html#supplementary-information%5Cnhttp://www.nature.com/nbt/journal/v32/n8/full/nbt.2942.html?WT.ec_id=NBT-201408%5Cnhttp://www.ncbi.nlm.nih.gov

25. Darmon E, Leach DRF. Bacterial Genome Instability. Microbiol Mol Biol Rev [Internet]. 2014;78:1–39. Available from: http://mmbr.asm.org/cgi/doi/10.1128/MMBR.00035-13

26. Whitfield C, Valvano M a. Species-Wide Variation in the Escherichia coli Flagellin. Adv Microb Physiol. 2003;35:135–246.

27. Reid SD, Selander RK, Whittam TS. Sequence diversity of flagellin (fliC) alleles in pathogenic Escherichia coli. J Bacteriol. 1999;181:153–60.

28. Beutin L, Delannoy S, Fach P. Sequence variations in the flagellar antigen genes fliC<inf>H25</inf> and fliC<inf>H28</inf> of Escherichia coli and their use in identification and characterization of enterohemorrhagic E. Coli (EHEC) O145:H25 and O145:H28. PLoS One. 2015;10.

29. Heinrichs DE, Yethon JA, Whitfield C. Molecular basis for structural diversity in the core regions of the lipopolysaccharides of Escherichia coli and Salmonella enterica. Mol. Microbiol. 1998. p. 221–32.

30. Iguchi A, Iyoda S, Kikuchi T, Ogura Y, Katsura K, Ohnishi M, et al. A complete view of the genetic diversity of the Escherichia coli O-antigen biosynthesis gene cluster. DNA Res. 2015;22:101–7.

31. Huynen M, Snel B, Lathe W, Bork P. Predicting protein function by genomic context: Quantitative evaluation and qualitative inferences. Genome Res. 2000;10:1204–10.

32. Delihas N. Impact of small repeat sequences on bacterial genome evolution. Genome Biol Evol. 2011;3:959–73.

33. Wang D, Li S, Guo F, Ning K, Wang L. Core-genome scaffold comparison reveals the prevalence that inversion events are associated with pairs of inverted repeats. BMC Genomics [Internet]. BMC Genomics; 2017;18:268. Available from: http://bmcgenomics.biomedcentral.com/articles/10.1186/s12864-017-3655-0

34. Doron S, Melamed S, Ofir G, Leavitt A, Lopatina A, Keren M, et al. Systematic discovery of antiphage defense systems in the microbial pangenome. Science (80-). 2018;1–17.

35. Serruto D, Serino L, Masignani V, Pizza M. Genome-based approaches to develop vaccines against bacterial pathogens. Vaccine. 2009. p. 3245–50.

36. Maione D, Margarit I, Rinaudo CD, Masignani V, Scarselli M, Tettelin H, et al. Identification of a Universal Group B Streptococcus Vaccine by Multiple Genome Screen. 2006;309:148–50.

37. Kovatcheva-Datchary P, Nilsson A, Akrami R, Lee YS, De Vadder F, Arora T, et al. Dietary Fiber-Induced Improvement in Glucose Metabolism Is Associated with Increased Abundance of Prevotella. Cell Metab. 2015;22:971–82.

38. Pedersen HK, Gudmundsdottir V, Nielsen HB, Hyotylainen T, Nielsen T, Jensen BAH, et al. Human gut microbes impact host serum metabolome and insulin sensitivity. Nature. 2016;535:376–81.

39. Laursen BS, Sørensen HP, Mortensen KK, Sperling-Petersen HU. Initiation of protein synthesis in bacteria. Microbiol Mol Biol Rev [Internet]. 2005;69:101–23. Available from: http://www.scopus.com/inward/record.url?eid=2-s2.0-14844340954&partnerID=tZOtx3y1

40. De Boer HA, Hui AS. Sequences within ribosome binding site affecting messenger RNA translatability and method to direct ribosomes to single messenger RNA species. Methods Enzymol.

1990;185:103–14.

41. Berwal SK, Sreejith RK, Pal JK. Distance between RBS and AUG plays an important role in overexpression of recombinant proteins. Anal Biochem. 2010;405:275–7.

42. Zhu W, Lomsadze A, Borodovsky M. Ab initio gene identification in metagenomic sequences. Nucleic Acids Res. 2010;38.

43. Li W, Godzik A. Cd-hit: A fast program for clustering and comparing large sets of protein or nucleotide sequences. Bioinformatics. 2006;22:1658–9.

44. Kent WJ. BLAT - The BLAST-like alignment tool. Genome Res. 2002;12:656–64.

45. Mende DR, Waller AS, Sunagawa S, Järvelin AI, Chan MM, Arumugam M, et al. Assessment of metagenomic assembly using simulated next generation sequencing data. PLoS One. 2012;7.

46. R: The R Project for Statistical Computing [Internet]. [cited 2018 Mar 6]. Available from: https://www.r-project.org/

 Click here to download Table Table 1.xlsx

Table 1. Comparison of serveral pangenome analysis methods.

**Method**	**Input**	
	Isolate genomes	Metagenomes
GET_HOMOLOGUES [2] and PGAP [3]	Yes	No
GenoSets [4], PGAT [5], PEGR [6], EDGAR [7], GenomeRing [8]	Yes	No
PanViz [9]	Yes	No
SplitMEM10 and a tool introduced by Baier *et al.* [11]	Yes	No
PanPhlAn [12], MIDAS [13] and a method introduced by Farag *et al.* [16]	No	Yes
MetaPGN	Yes	Yes

Output			Functionality	
Gene content	Gene-gene adjacency	Network	Biological annotation	Interactive visualization
Yes	No	No	Yes	No
Yes	Yes	No	Yes	No
Yes	Yes	No	Yes	Yes
Yes	Yes	Yes	No	Yes
Yes	No	No	Yes	No
Yes	Yes	Yes	Yes	Yes

Figure 1

Click here to download Figure Figure 1.pdf

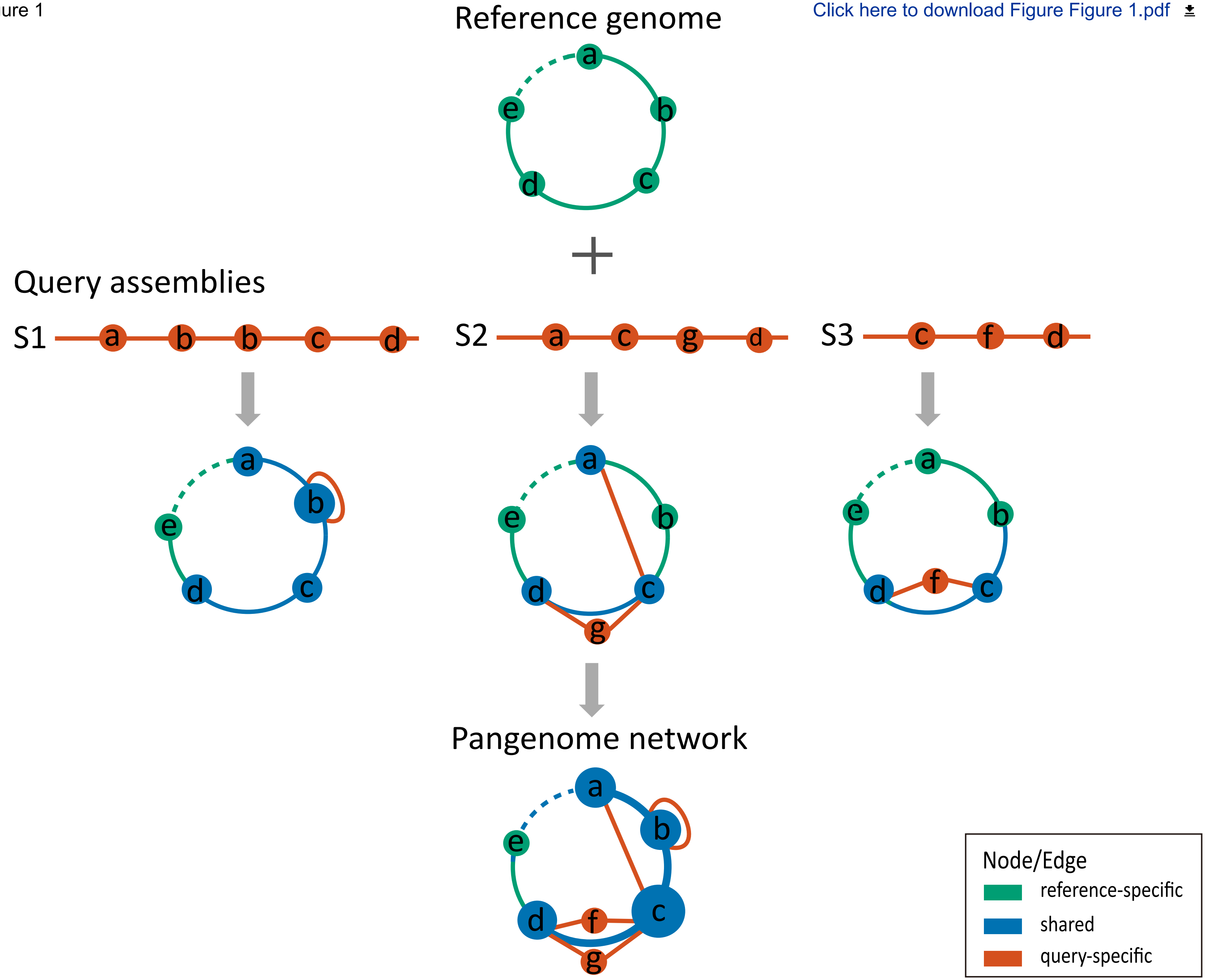

Node					
Name	Freq.	From	Gene	Annotation	...
a	3	S1, S2, Ref	*fliA*	Sigma factor 28	...
b	2	S1, Ref	*fliD*	Flagellar capping protein	...
c	4	S1, S2, S3, Ref	*fliC*	Flagellin	...
d	4	S1, S2, S3, Ref	*fliZ*	Flagellar assembly chaperone	...
e	1	Ref	Unknown	Unknown	...
...	...	...	...	...	...

Edge				
Source	Target	Freq.	From	...
a	b	2	S1, Ref	...
b	c	2	S1, Ref	...
b	b	1	S1	...
c	d	2	S1, Ref	...
a	c	1	S2	...
...	...			...

Figure 2

Click here to download Figure Figure 2.pdf

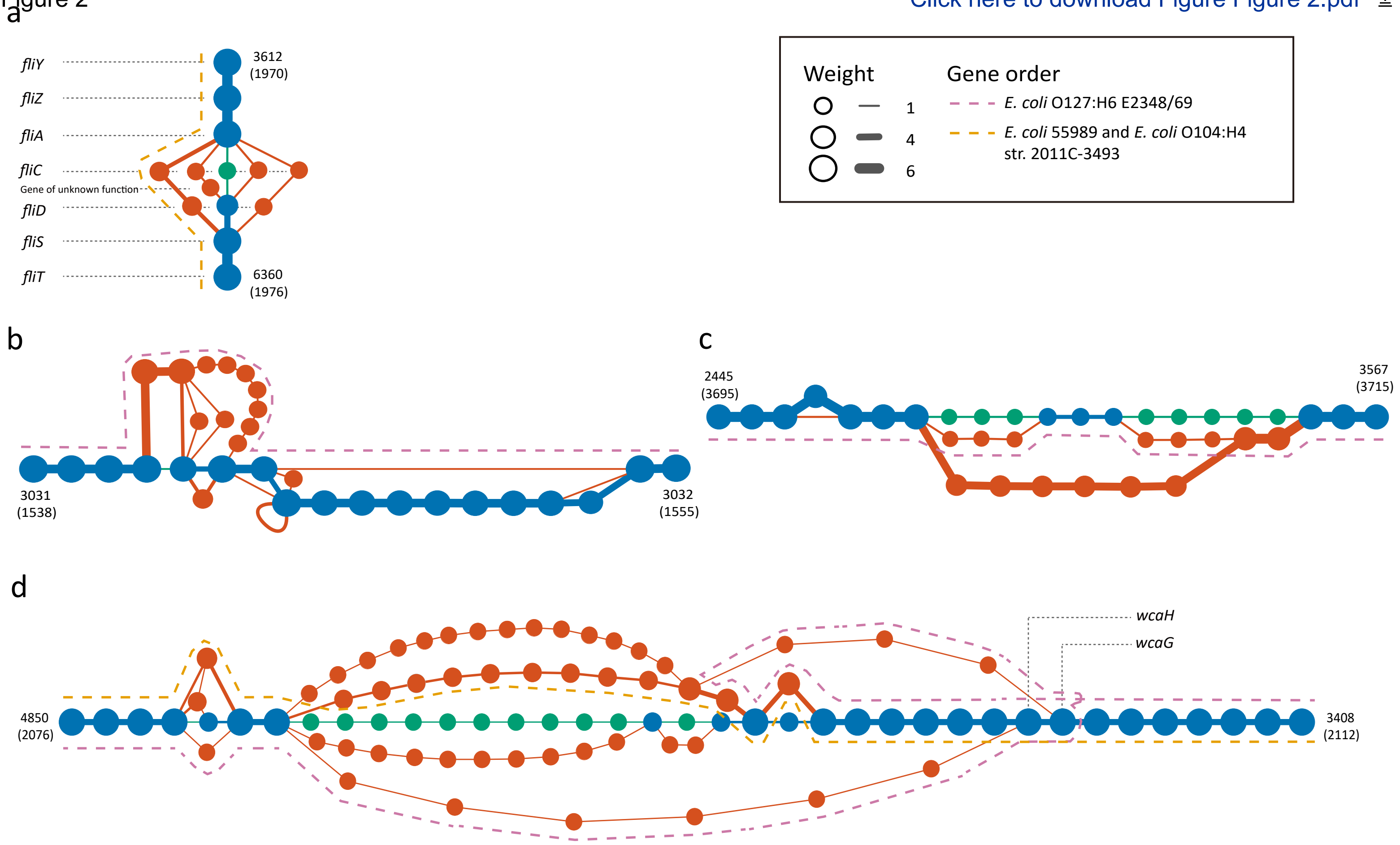

Figure 3

Click here to download Figure Figure 3.pdf

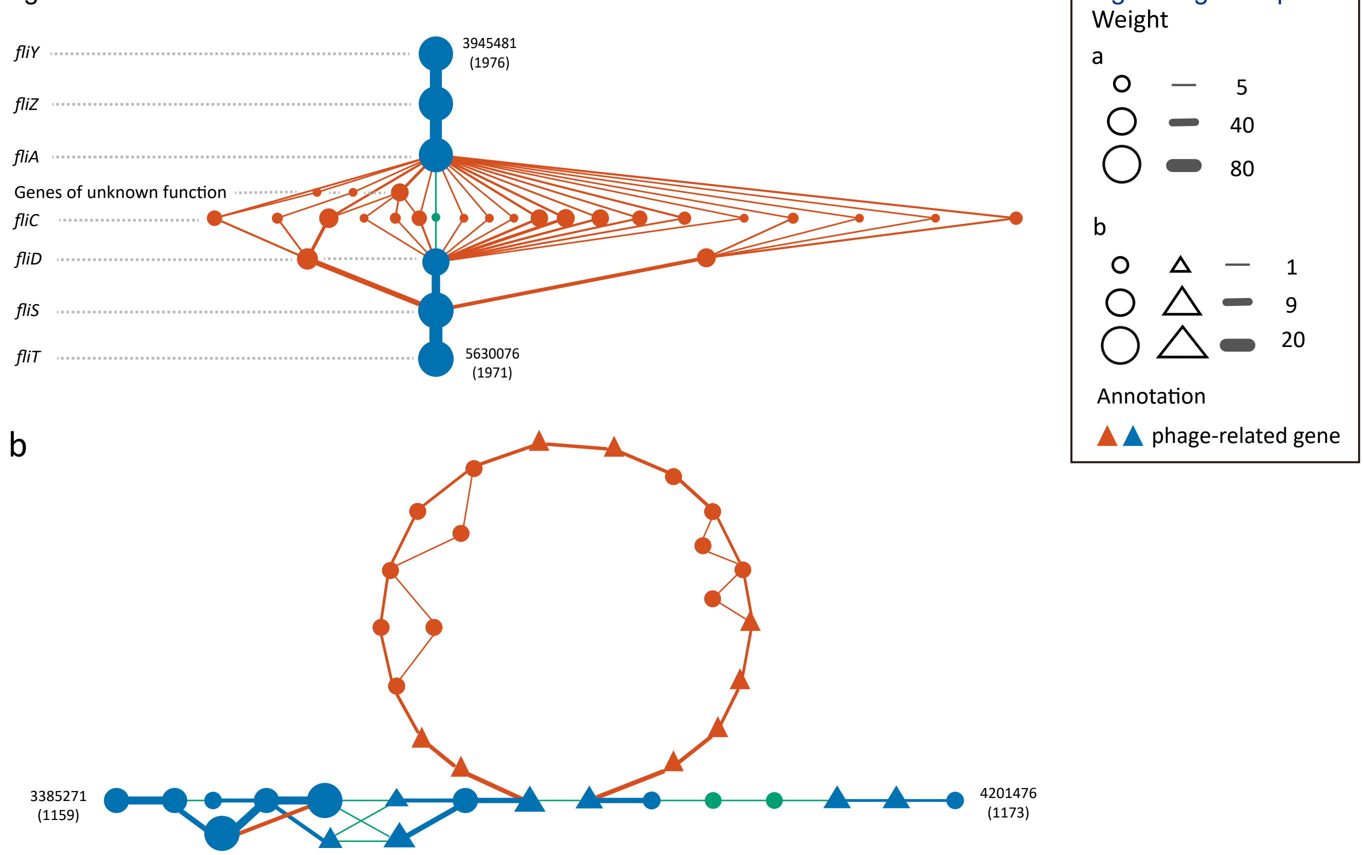

Figure 4

Click here to download Figure Figure 4.pdf

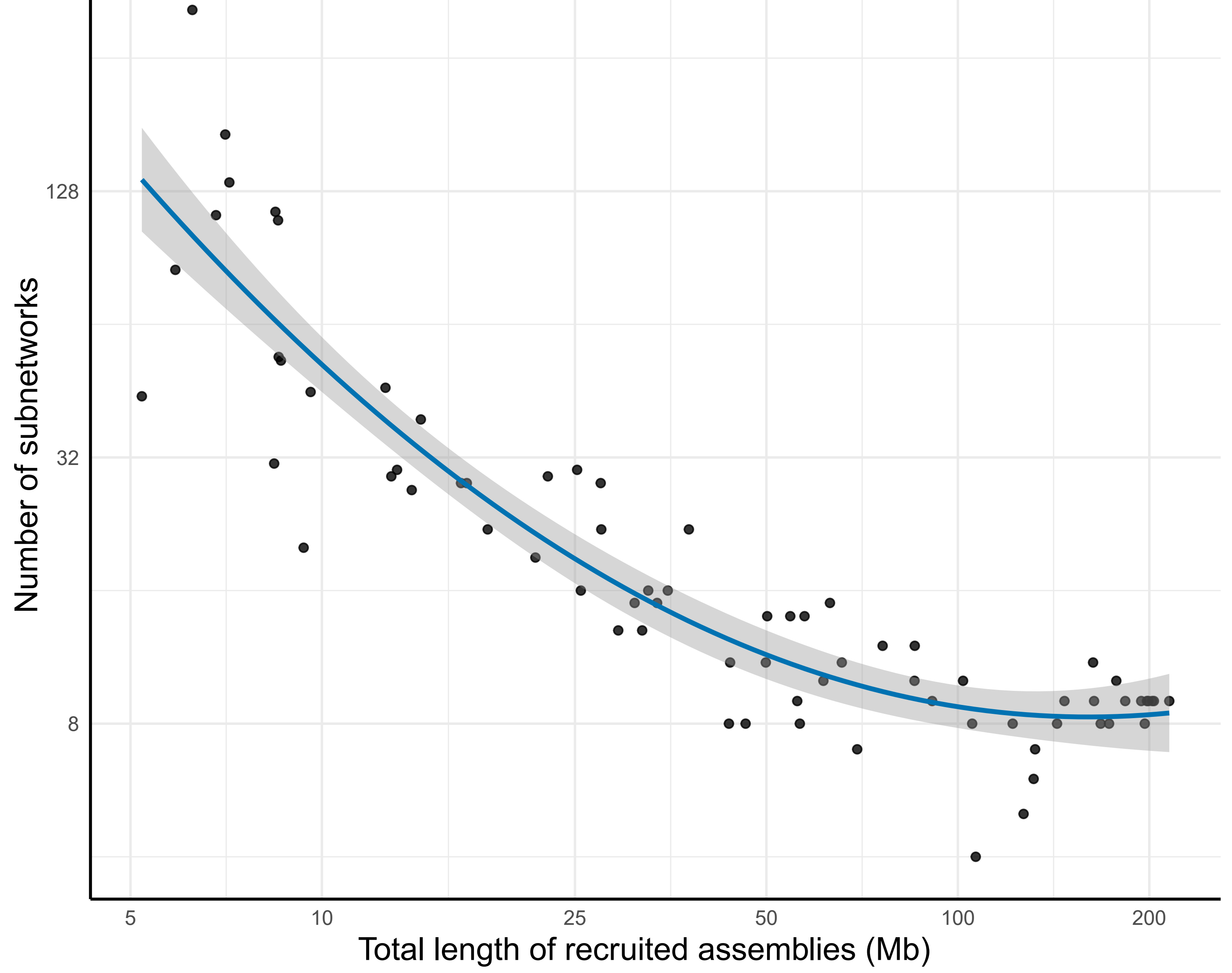

Supplementary Figure S1

Click here to access/download
Supplementary Material
Supplementary Figure S1.pdf

Supplementary Figure S2

Click here to access/download
Supplementary Material
Supplementary Figure S2.pdf

Supplementary Table S1

Click here to access/download

Supplementary Material

Supplementary Table S1.xlsx

Supplementary Table S2

Click here to access/download
Supplementary Material
Supplementary Table S2.xlsx

Supplementary Table S3

Click here to access/download
Supplementary Material
Supplementary Table S3.xlsx

Click here to access/download
Supplementary Material
Supplementary File S1.docx

Click here to access/download
Supplementary Material
Supplementary File S2.pdf

Click here to access/download
Supplementary Material
Supplementary File S3.pdf
