# Supplementary material for: MetaPGN: a pipeline for construction and graphical visualization of annotated pangenome networks
Source: Gigascience. 2018 Oct 2;7(11):giy121. doi: 10.1093/gigascience/giy121 (PMC6251982; doi:10.1093/gigascience/giy121)

**Supplementary Text 1. Steps for constructing pangenome networks.**

1. Download MetaPGN and get prerequisites
   1. Download MetaPGN from <https://github.com/peng-ye/MetaPGN/>.
   2. Download and install Perl (version 5.0 or above) from <https://www.perl.org/get.html>.
   3. Download MetaGeneMark program as well as a license key from <http://exon.gatech.edu/license_download.cgi>.
   4. From decompressed files of MetaGeneMark, copy the key “*gm_key[_64/_32]”* to your home directory, and copy the program “*gmhmmp*” and “*MetaGeneMark_v1.mod*” to the directory “Scripts/basic_src” in MetaPGN.

cp **gm_key**[_64/_32] ~/.gm_key

cp **gmhmmp** **MetaGeneMark_v1.mod** path/to/MetaPGN/Scripts/basic_src

1. Execute the script named “*MetaPGN_flow.pl”* to generate a shell script (e.g., “*MetaPGN.sh”*) containing necessary steps in the pipeline (the help text for “*MetaPGN_flow.pl”* is shown below).
   1. Usage

*perl MetaPGN_flow.pl -q <input query assemblies sequence> -qt <input query assemblies type> -ref <input reference sequence> -p <output prefix> <Other options> >work.sh*

- 1. Explanation of options

*-type <str> sequence type of input query assemblies, nt (nucleotide sequence) or aa (amino acid sequence), (default nt)*

*-q <str> input file (in FASTA format)* *or file list (with the suffix .list) for query sequences*

*-qt <str> source of the input sequence, genome or metagenome (default genome)*

*-gene <str> type of the input sequence, gene or assembly (default assembly)*

*-ref <str> input file (in FASTA format) for the reference sequence*

*-meta <str> input file (in metadata format) for metadata of the reference sequence*

*-p <str> output prefix*

*-M <int> minimum gene length in gene prediction (default 100)*

*-qs <str> qsub or local, when run the Step2. Gene redundancy elimination (default local)*

*-qsp <str> project name for qsub -P (default none)*

*-qsq <str> queue name for qsub -q (default none)*

*-div <int> when you choose -qs qsub, it will divide your gene prediction results into segment (default 10)*

*-aS <float> alignment coverage for the shorter sequence (default, nt: 0.9, aa: 0.7)*

*if set to 0.9, the alignment must cover 90% of the sequence*

*-i <float> sequence identity threshold (default, nt: 0.95, aa: 0.7. See Cd-hit manual for more details)*

*-n <int> word_length (default nt: 8, aa: 5. See Cd-hit manual for more details)*

*-d <int> length of description in the *.clstr file (default 0)*

*if set to 0, it takes the fasta defline and stops at first space (See Cd-hit manual for more details)*

*-g1 <int> 1 or 0 (default 1)*

*Based on cd-hit's default algorithm, a sequence is clustered to the first cluster that meet the threshold (fast cluster). If set to 1, the program will cluster it into the most similar cluster that meet the threshold (accurate but slow mode) but either 1 or 0 won't change the representatives of final clusters (See Cd-hit manual for more details)*

*-T <int> number of threads in cd-hit-est, with 0, all CPUs will be used (default 6. See Cd-hit manual for more details)*

*-G <int> use global sequence identity (default 0. See Cd-hit manual for more details)*

*-c <int> cut off for the gene count on an assembly (default 3)*

*-r <float> cut off for the ratio of the number of shared genes on an assembly to the total number of genes on that assembly (default 0.5)*

*-h <str> display this help text and exit*

- 1. Examples

Example 1, the input sequence is nucleotide sequence of assemblies from metagenomes:

*perl MetaPGN_flow.pl -q Inputs/query.assemblies.list -qt metagenome -ref References/Gene_features/Escherichia_coli_K_12_substr__MG1655.genes.fasta -meta References/Annotations/Escherichia_coli_K_12_substr__MG1655.idx.metadata -p metagenome_assemblies_nt > MetaPGN.sh*

Example 2, the input sequence is nucleotide sequence of genes from isolated genomes:

*perl MetaPGN_flow.pl -q Inputs/genes/query.genes.nt.fasta -qt genome -ref References/Gene_features/Escherichia_coli_K_12_substr__MG1655.genes.fasta -p genome_genes_nt -gene gene > MetaPGN.sh*

Example 3, the input sequence is amino acid sequence of genes from metagenomes, and the script will use qsub to submit tasks in redundancy elimination of genes onto available computing clusters:

*perl MetaPGN_flow.pl -q Inputs/query.genes.aa.list -qt metagenome -type aa -ref References/Gene_features/Escherichia_coli_K_12_substr__MG1655.genes.prot.fasta -meta References/Annotations/Escherichia_coli_K_12_substr__MG1655.prot.idx.metadata -gene gene -qs qsub -div 3 -p genome_genes_aa > MetaPGN.sh*

1. Execute the shell script to construct pangenome networks.

Usage: *sh MetaPGN.sh [>MetaPGN.log 2> MetaPGN.err &]*

**Supplementary Text 2.** **Steps for installing the plug-in and visualizing pangenome networks in Cytoscape.**

1. Install Java 8 (http://www.oracle.com/technetwork/java/javase/downloads/jre8-downloads-2133155.html) and Cytoscape (http://www.cytoscape.org/download.php).
2. In the interface in Cytoscape,
   1. Install the plugin “*pgnVisApp.jar”* through "Apps > App Manager > Install Apps > Install from file…". These steps are demonstrated as follows:

*Open “App Manager”*


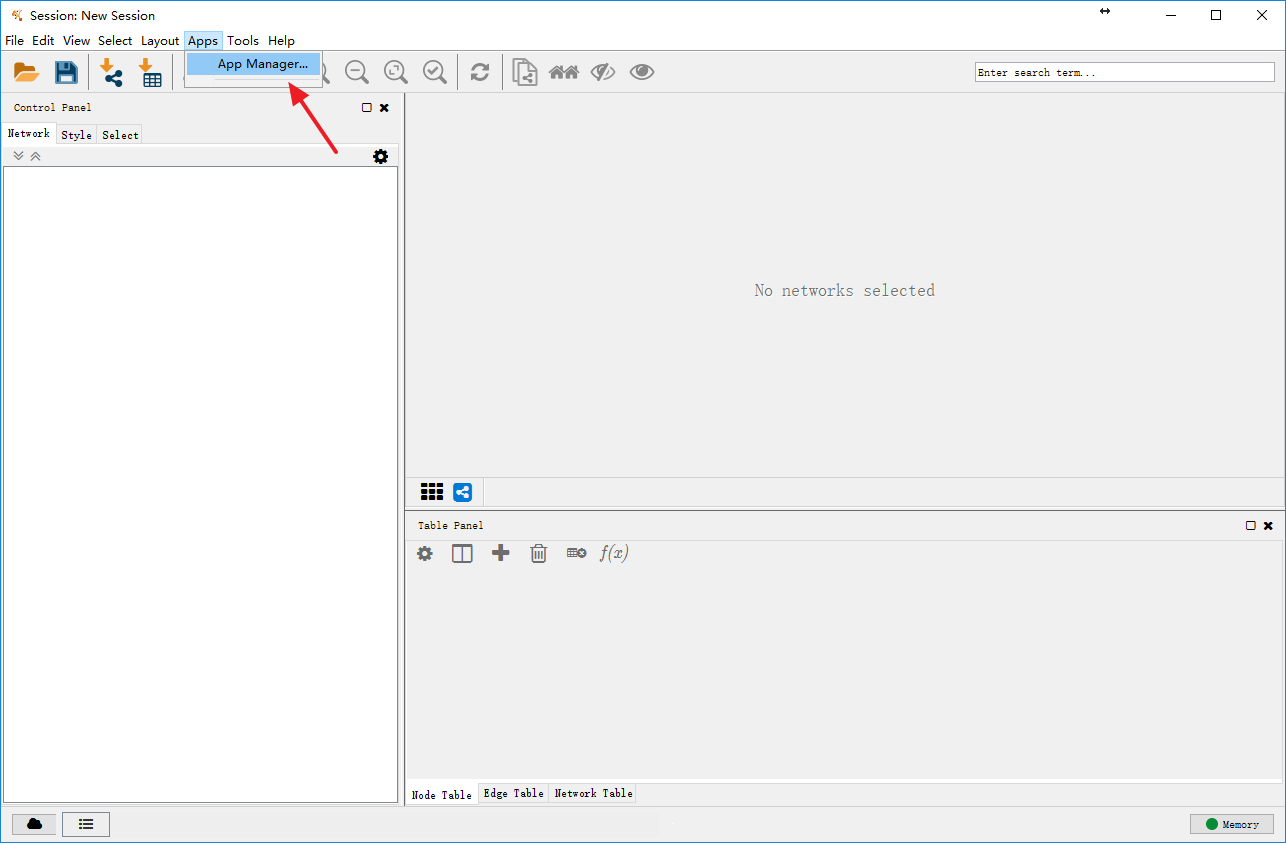


*Install “pgnVisApp.jar”*


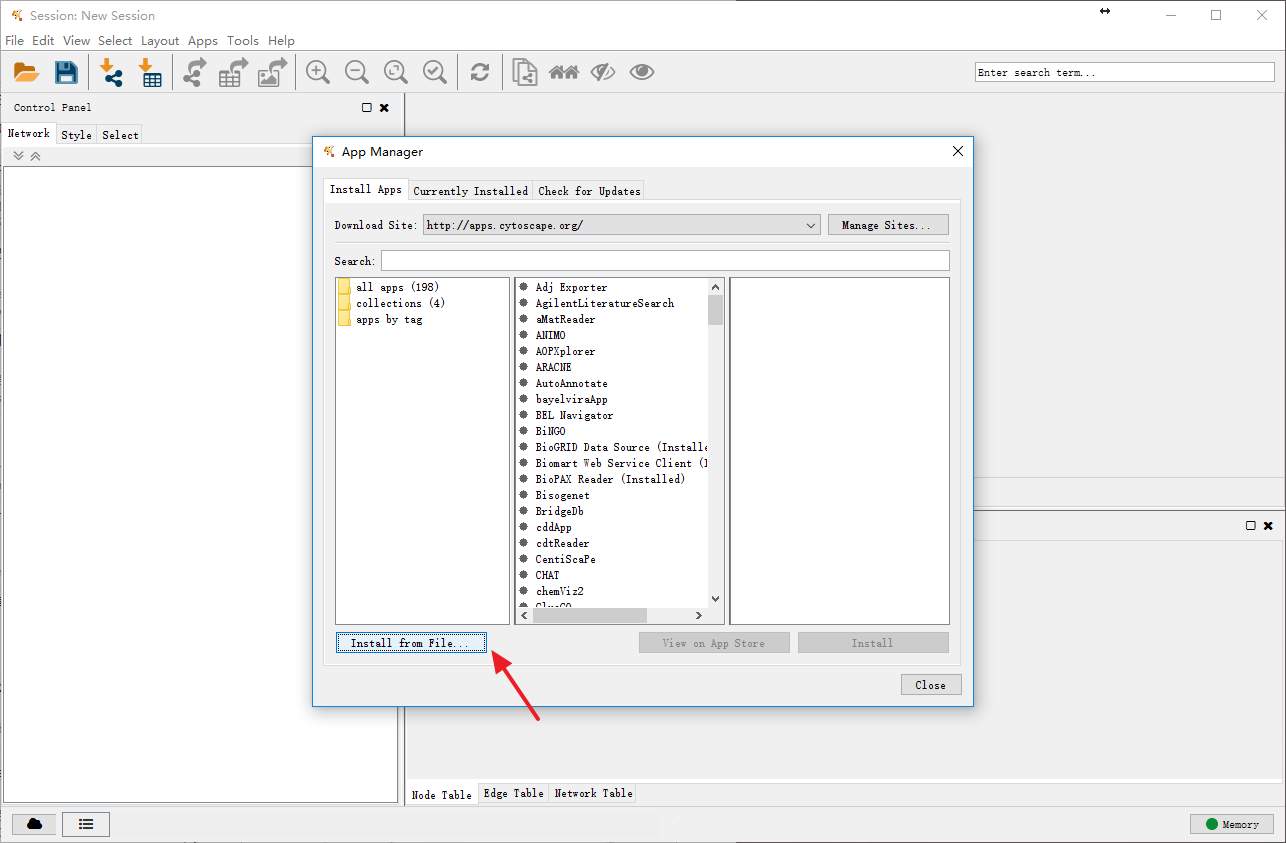


*The “pgnVisApp” appears at the “Currently Installed” panel*


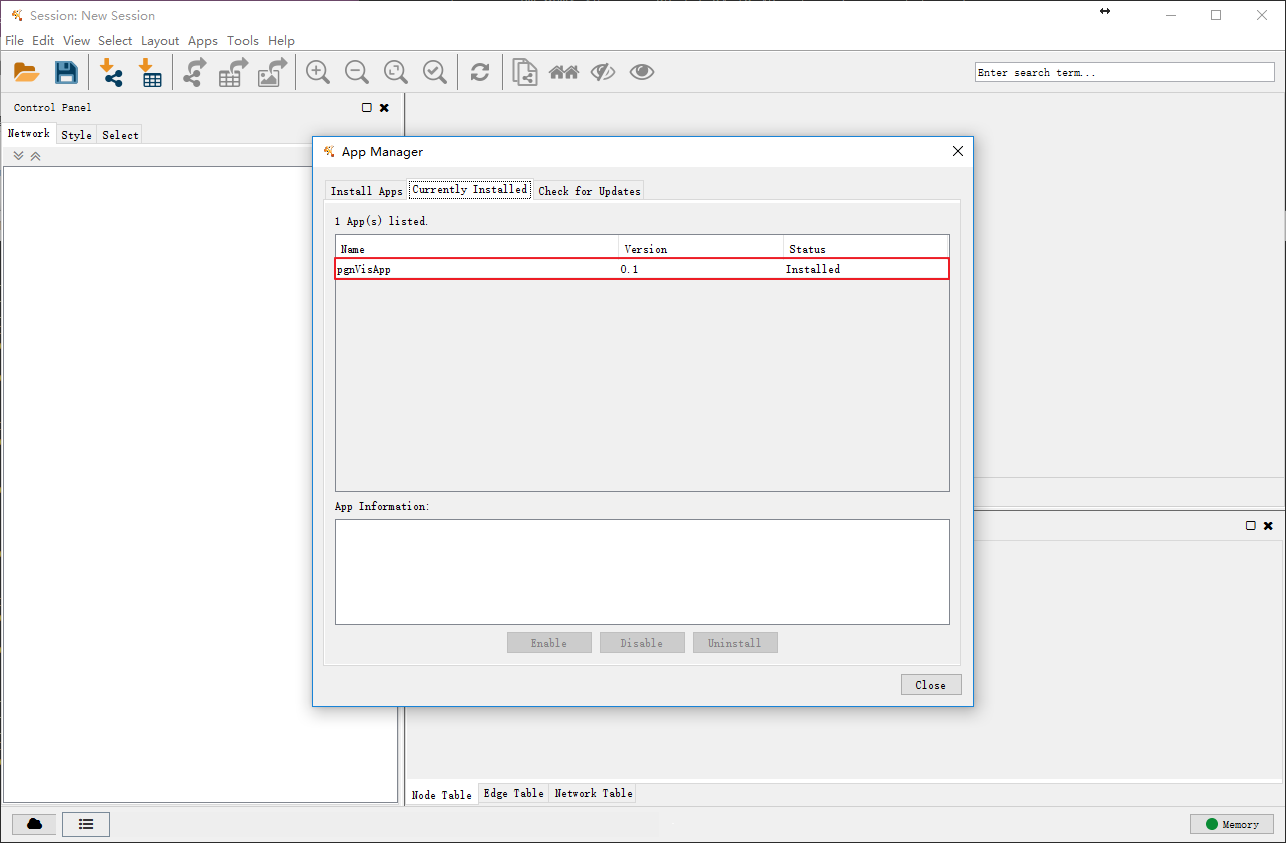


- 1. After installing, there should be an option named "Arrange node" in the "Select" menu.

*An option named “PGN Arrange nodes” in the “Select” menu after installing*


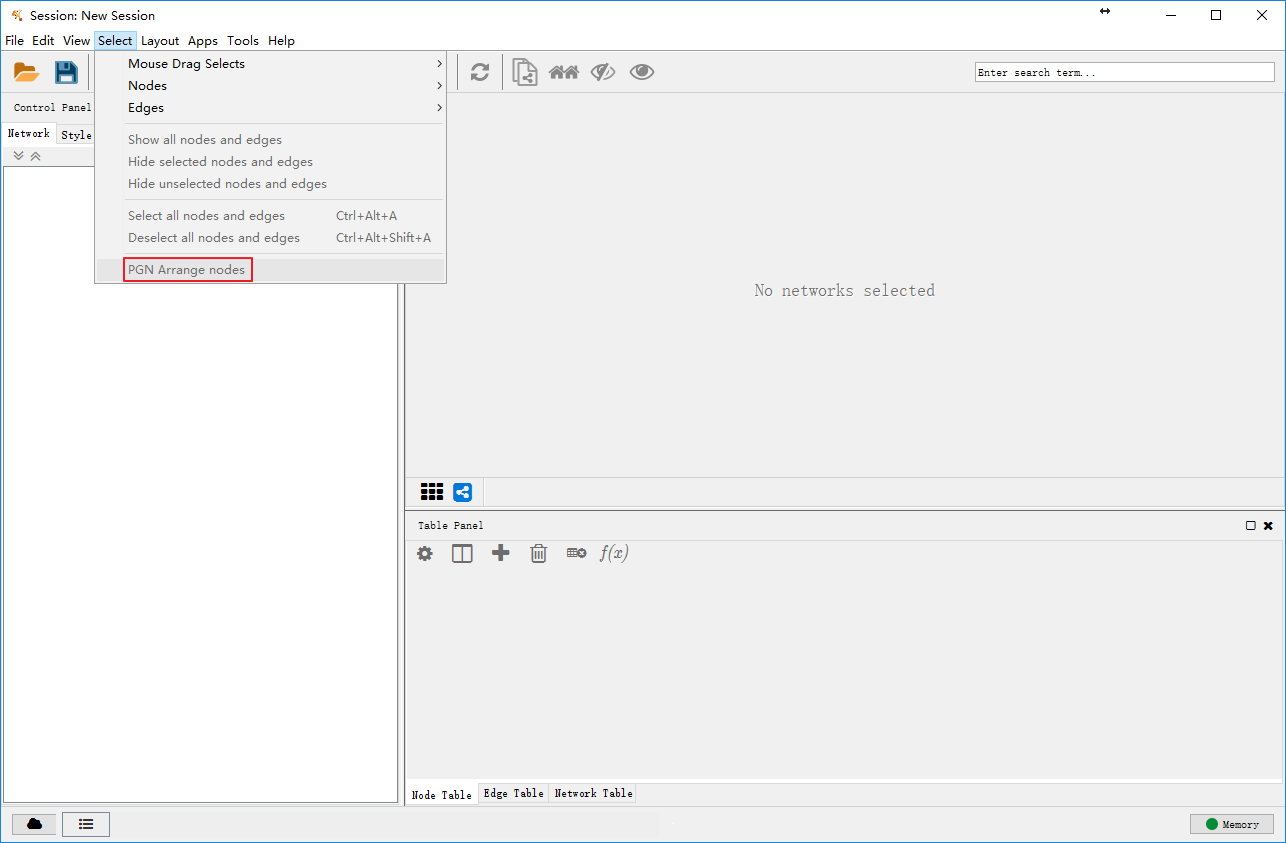


1. Import the network files into Cytoscape
   1. Get files of a pangenome network (used below are files for the pangenome network of the 5 *E. coli* strains. You can get it from Supplementary Table S3 or https://github.com/peng-ye/MetaPGN/).
   2. Import the edge file of the pangenome network through "File > Import > Network > file".

*Import the edge file*


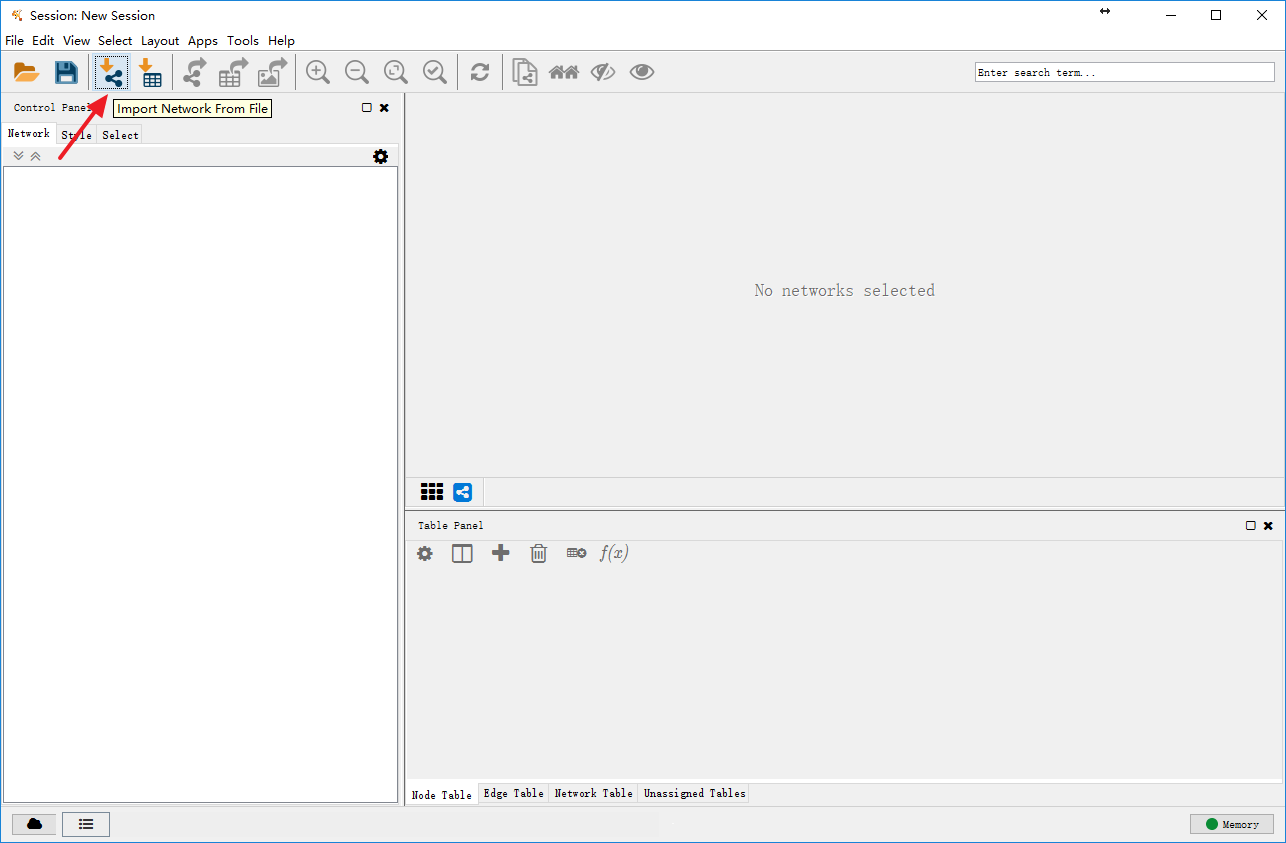


*Click “OK”*


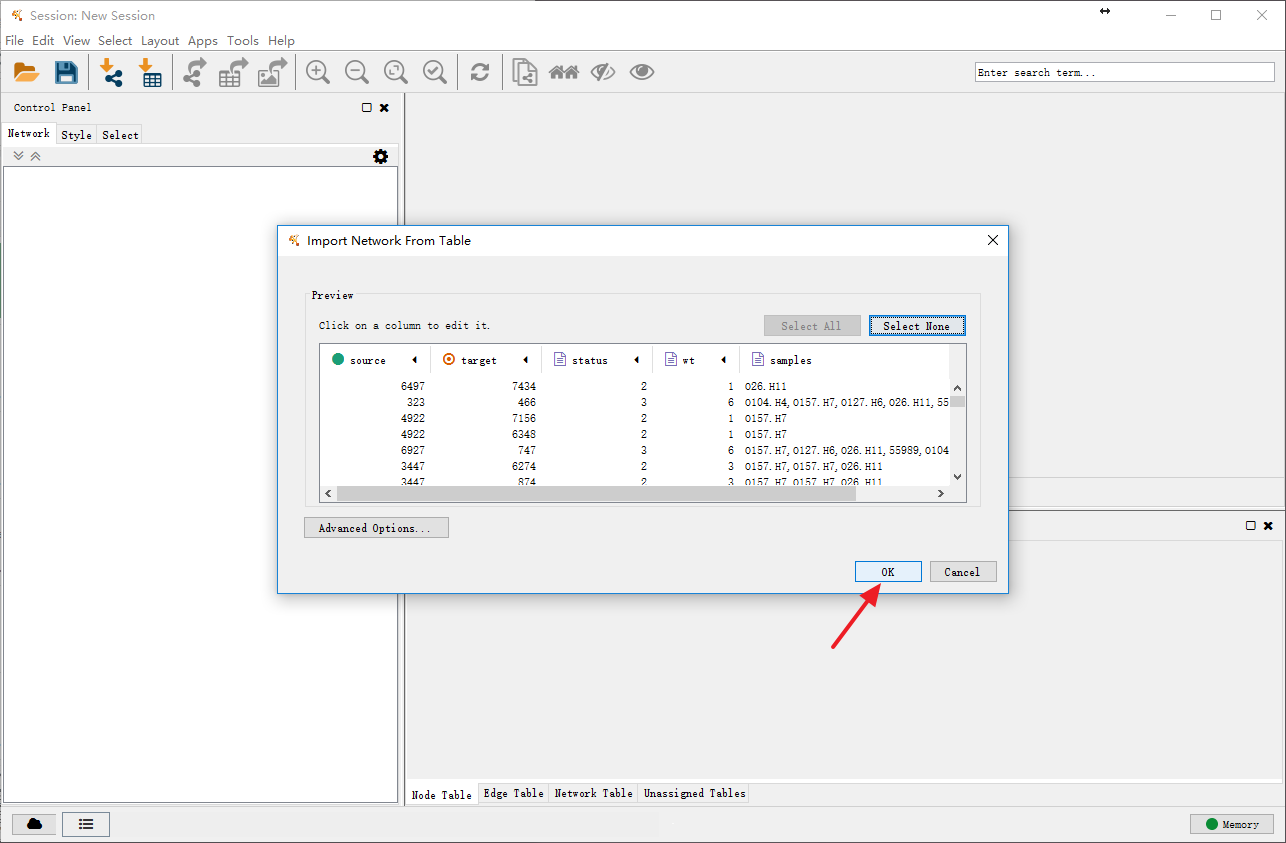


- 1. Import the node file of the pangenome network through "File > Import > Table > file".

*Import the node file (the interface shows the pangenome network before arrangement)*


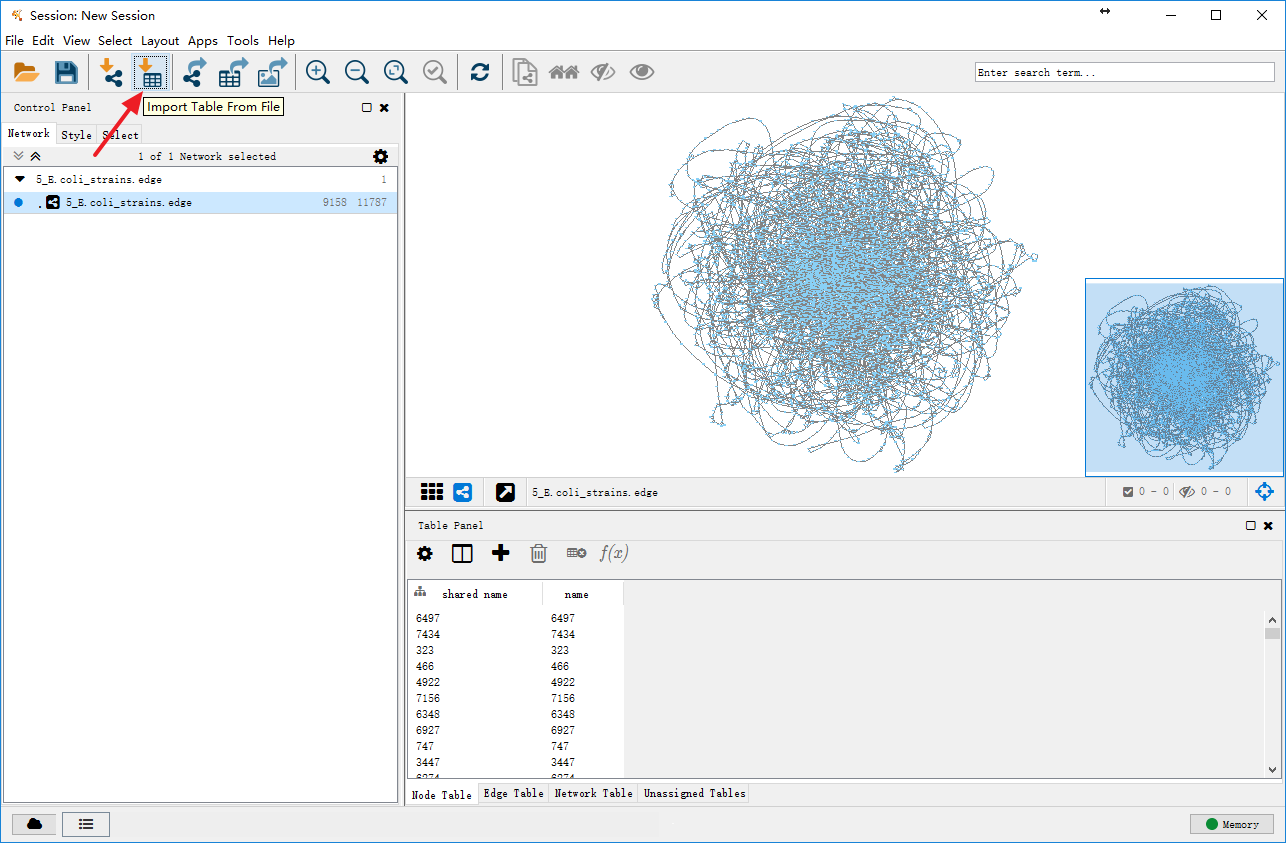


*Click “OK”*


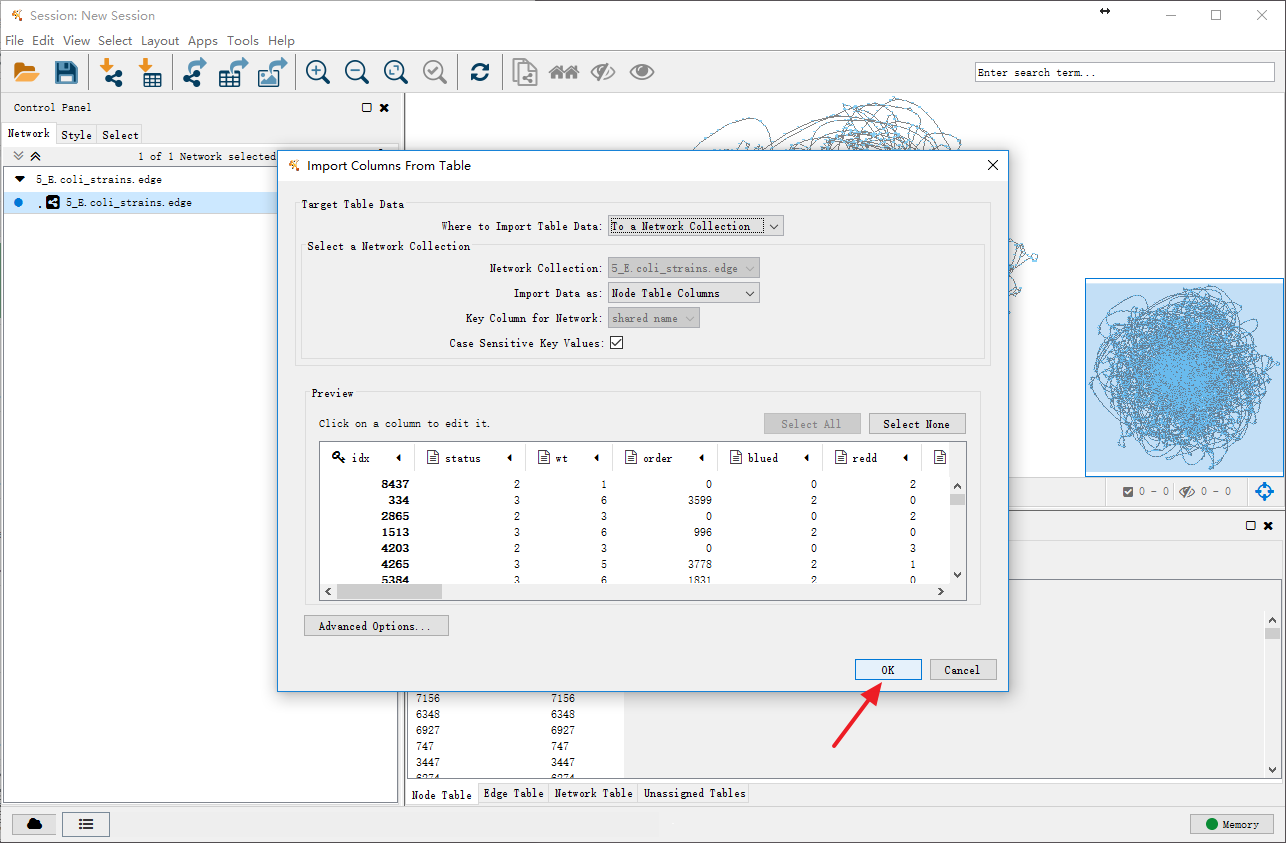


1. Arrange nodes
   1. In the "Node Table" in "Table Panel", add two columns named "search" and "coordinate" through "Create New Column > New Single Column > String", which will be used to store the arrangement and coordinate information.

*Add two string-type columns named* *“search” and “coordinate”*


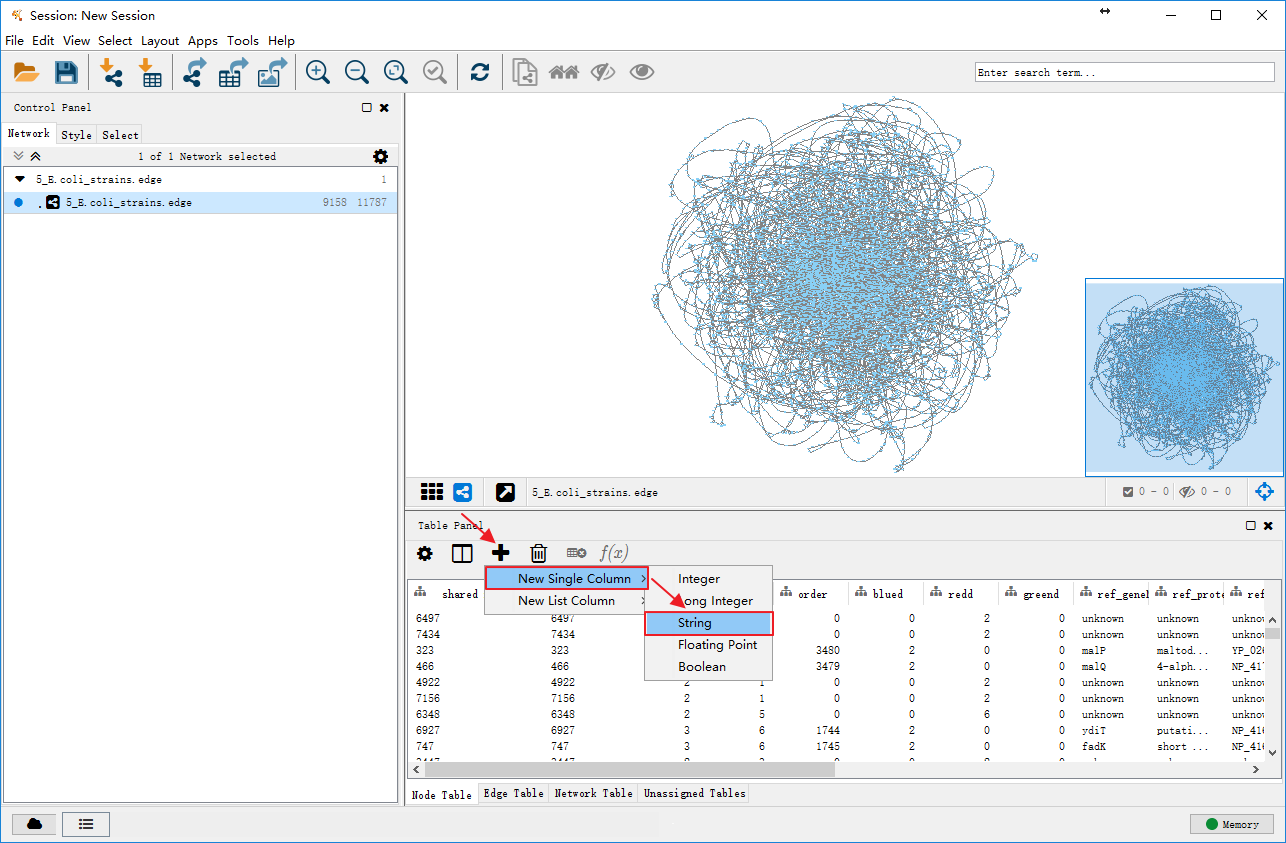


*Column “search” and “coordinate” appear at the “Node Table” panel*


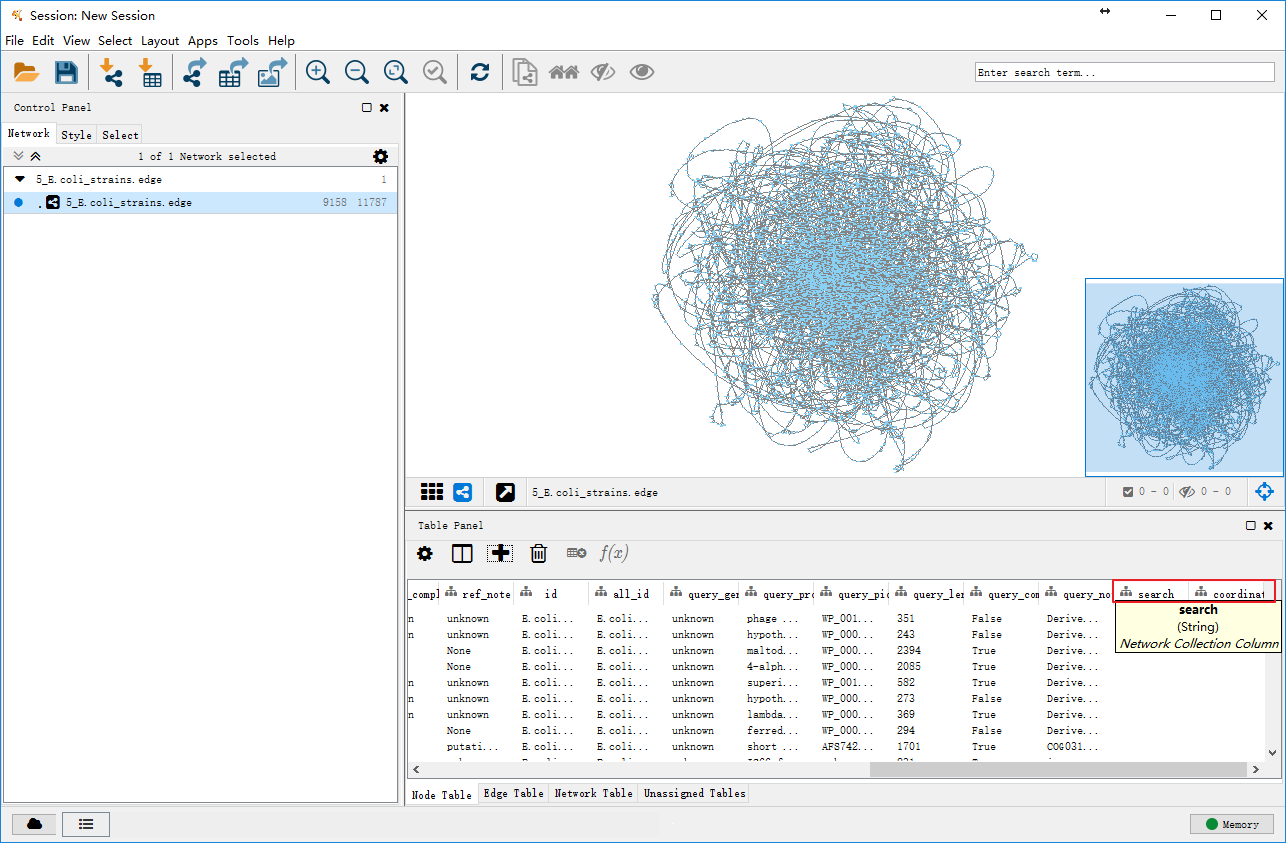


- 1. Click the option "PGN Arrange nodes" in the "Select" menu and the pangenome network will be arranged according to rules introduced in the paper.

*Click “PGN Arrange nodes”*


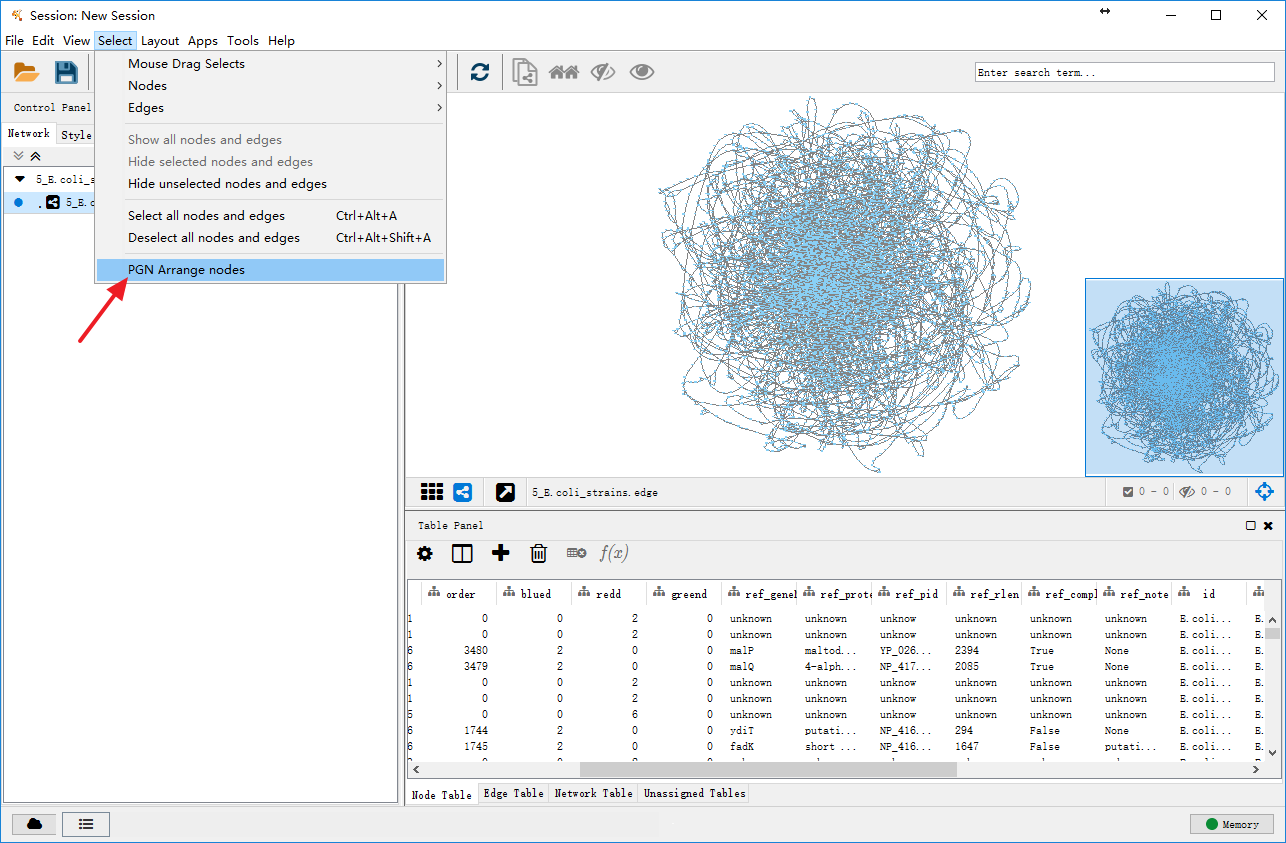


*The pangenome network after arrangement (Most of the nodes have been arranged, while others remain in the central area)*


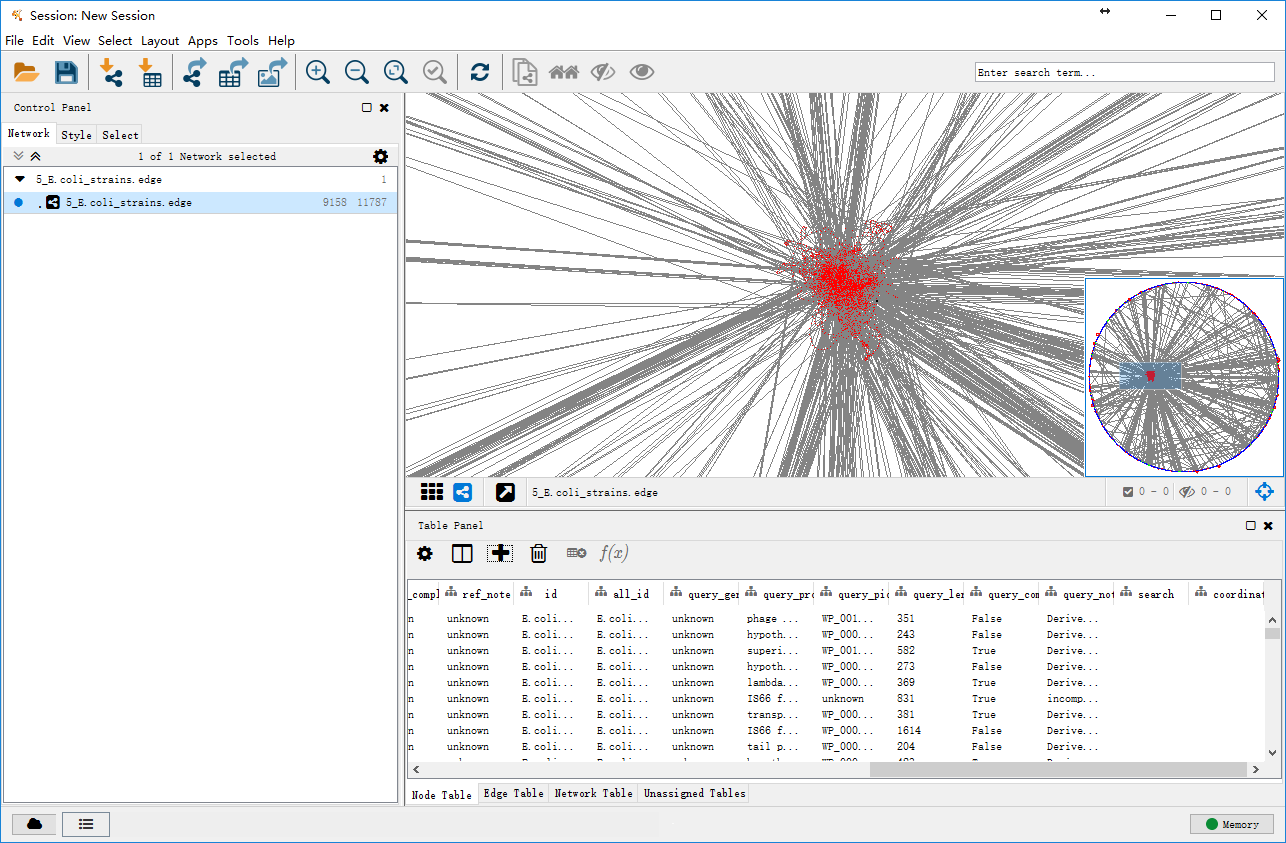


1. [Optional] Customize the shape of nodes and edges, search targeted nodes, and retrieve annotations of selected elements.
   1. In the “Style” panel of the “Control Panel” (red rectangle), customize the shape of nodes and edges based on their attributes of interest. The example below selects the “Ripple” style for the network (purple rectangle), customizes the fill color for nodes according to their status (green for “reference-specific”, orange for “query-specific” and blue for “shared” nodes) (dark blue rectangle), and specifies the size of nodes to reflect their weights in the network (green rectangle). More attributes can be customized and are not shown here (orange rectangle).


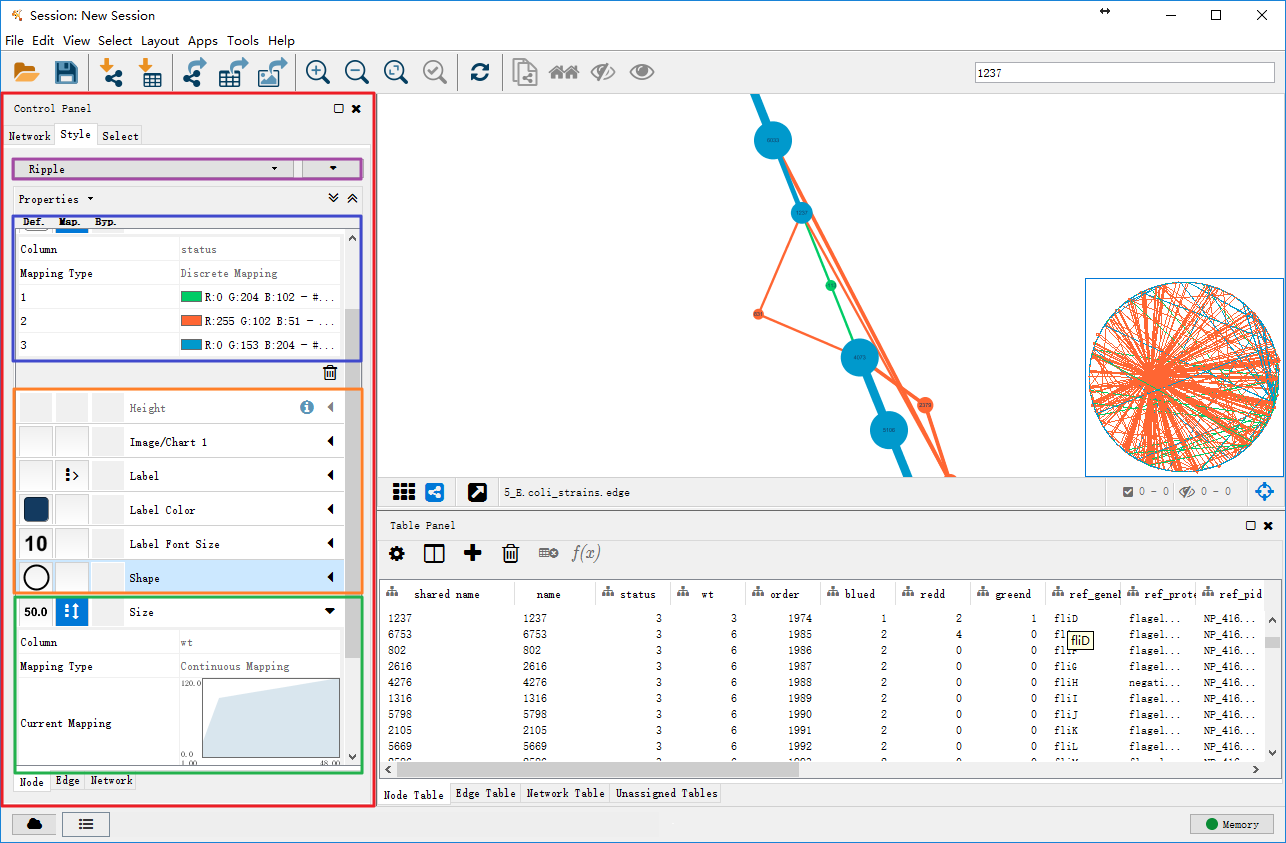


- 1. Select a node of interest from the “Table Panel” (dark blue dashed rectangle) or the original node file according to its annotation, type the ID (shared name) in the search box (red dashed rectangle) and press enter to locate the node (Shown below is Node 1237, which represents the sequence of gene *fliD*).

*Type the ID of the targeted node and press enter to locate the node*


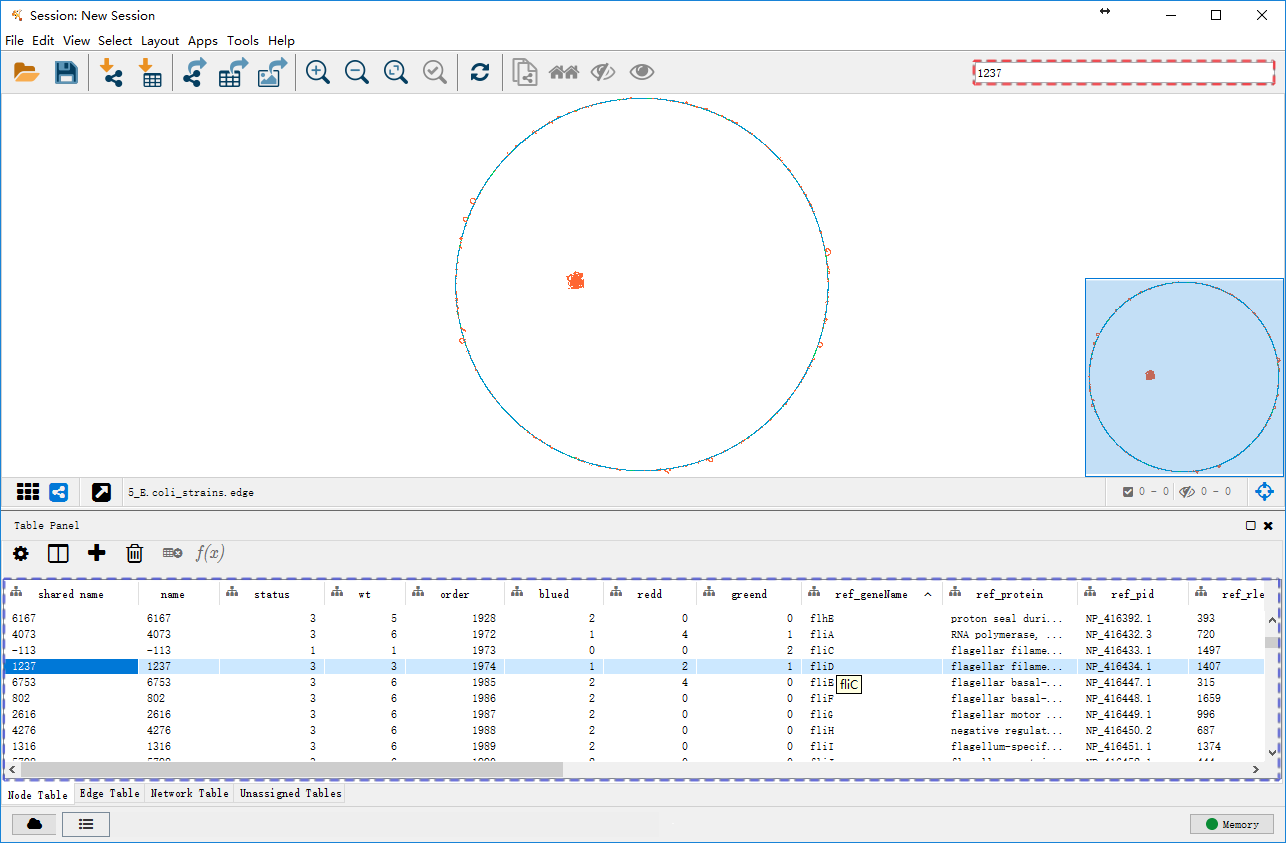


*Click “Zoom selected region” to show the node*


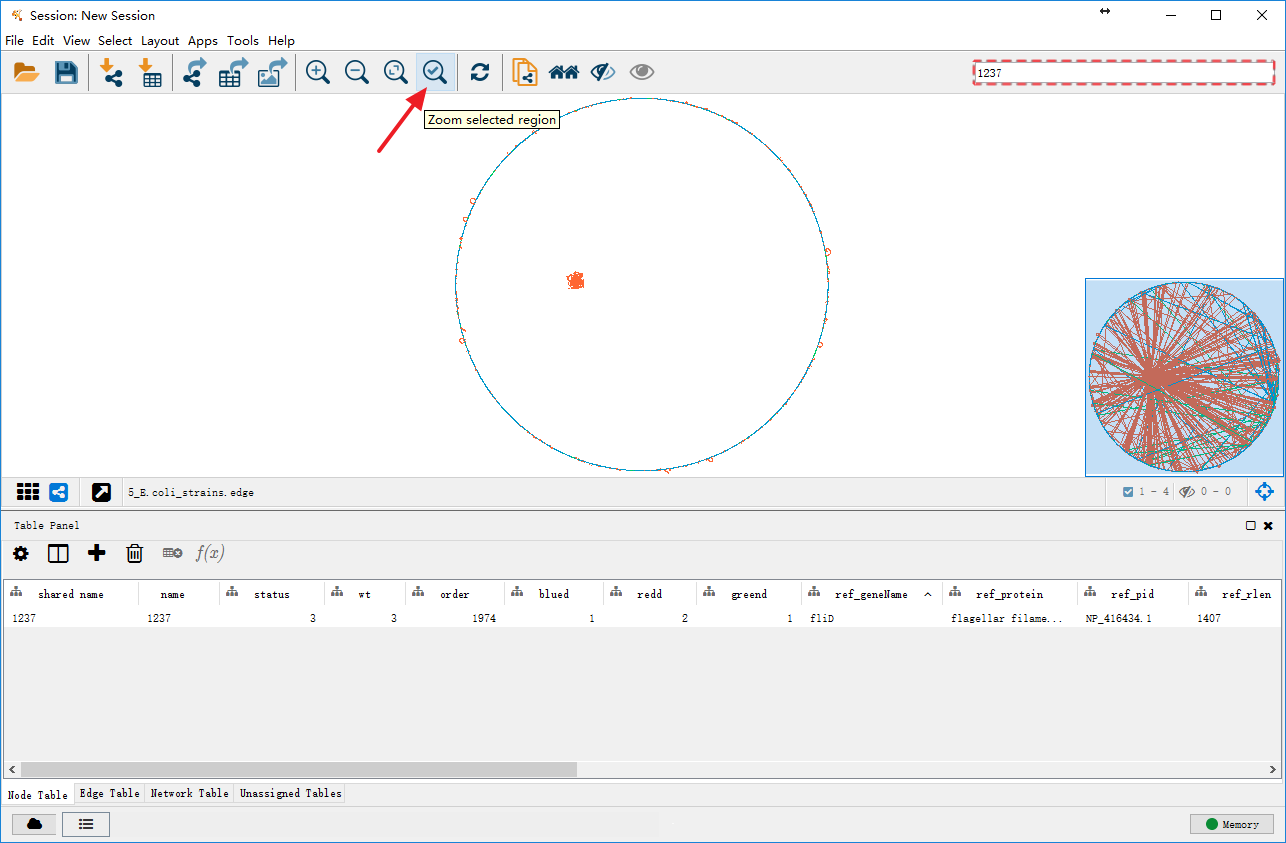


*Click “Zoom Out” or scroll down the mouse wheel to show its context*


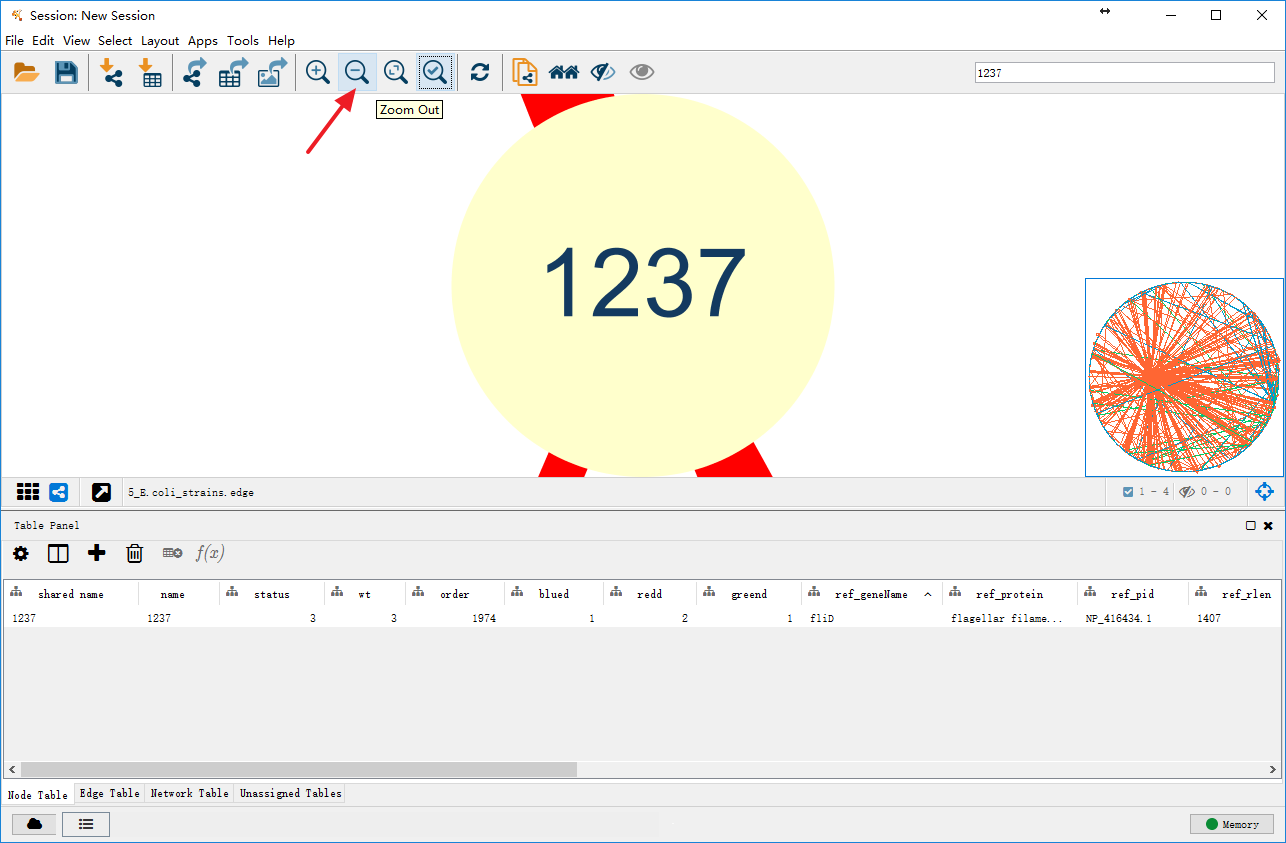


*The context of the selected node is shown after properly zooming*


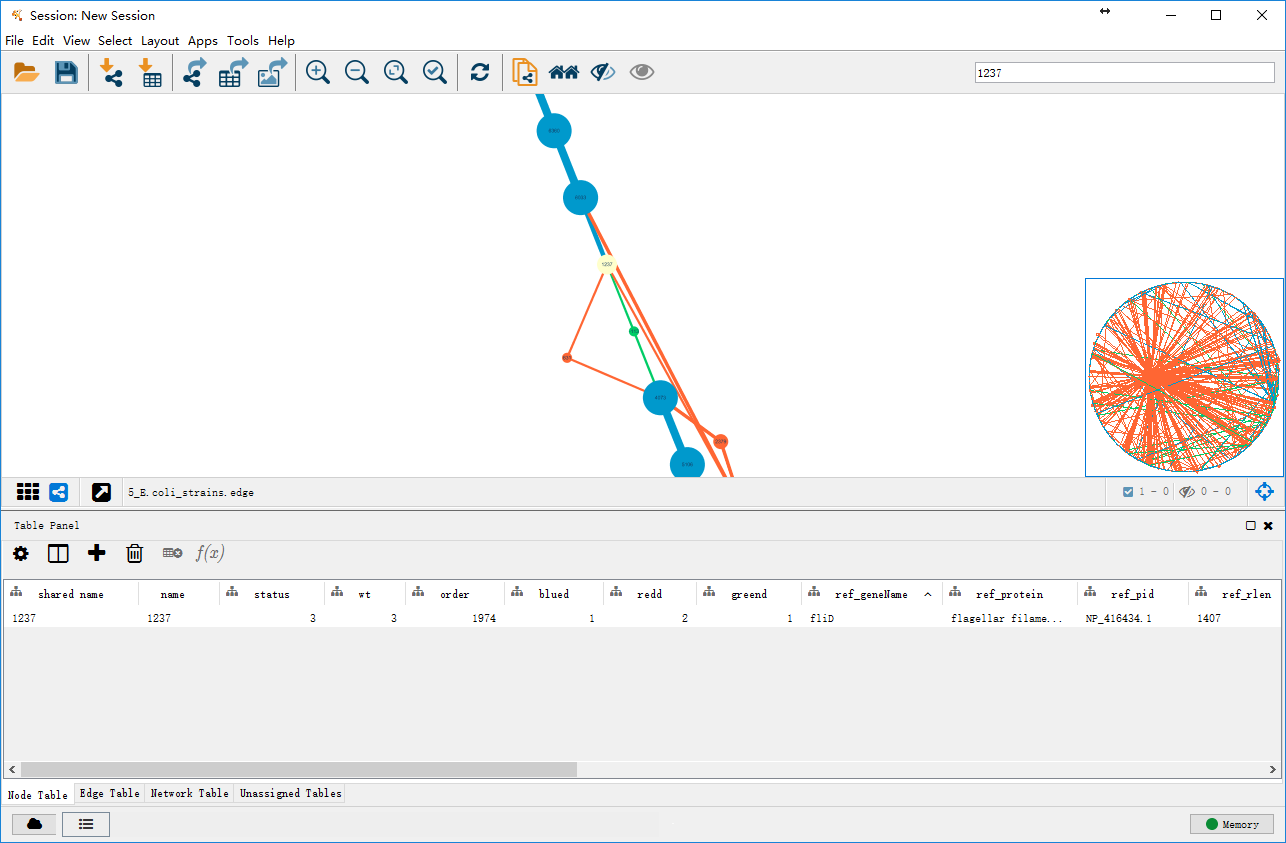


- 1. Select nodes (or edges) of interest to retrieve their annotation

*Select a node within an interesting loop, and click “First Neighbors of Selected Nodes” (for 4 times in this example), or press and hold “Ctrl” until done selecting nodes using the mouse*


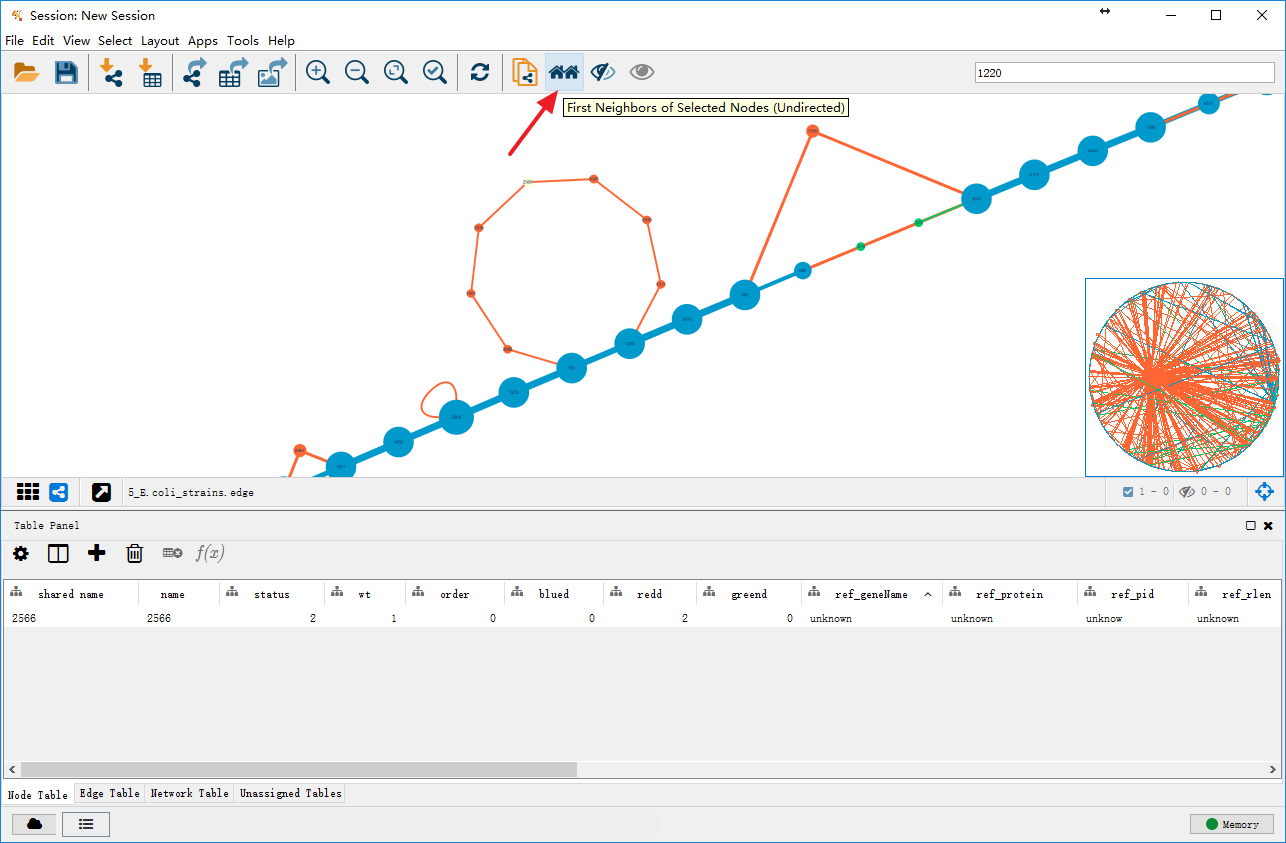


*Annotation of selected nodes in the “Table Panel” (dark blue dashed rectangle)*


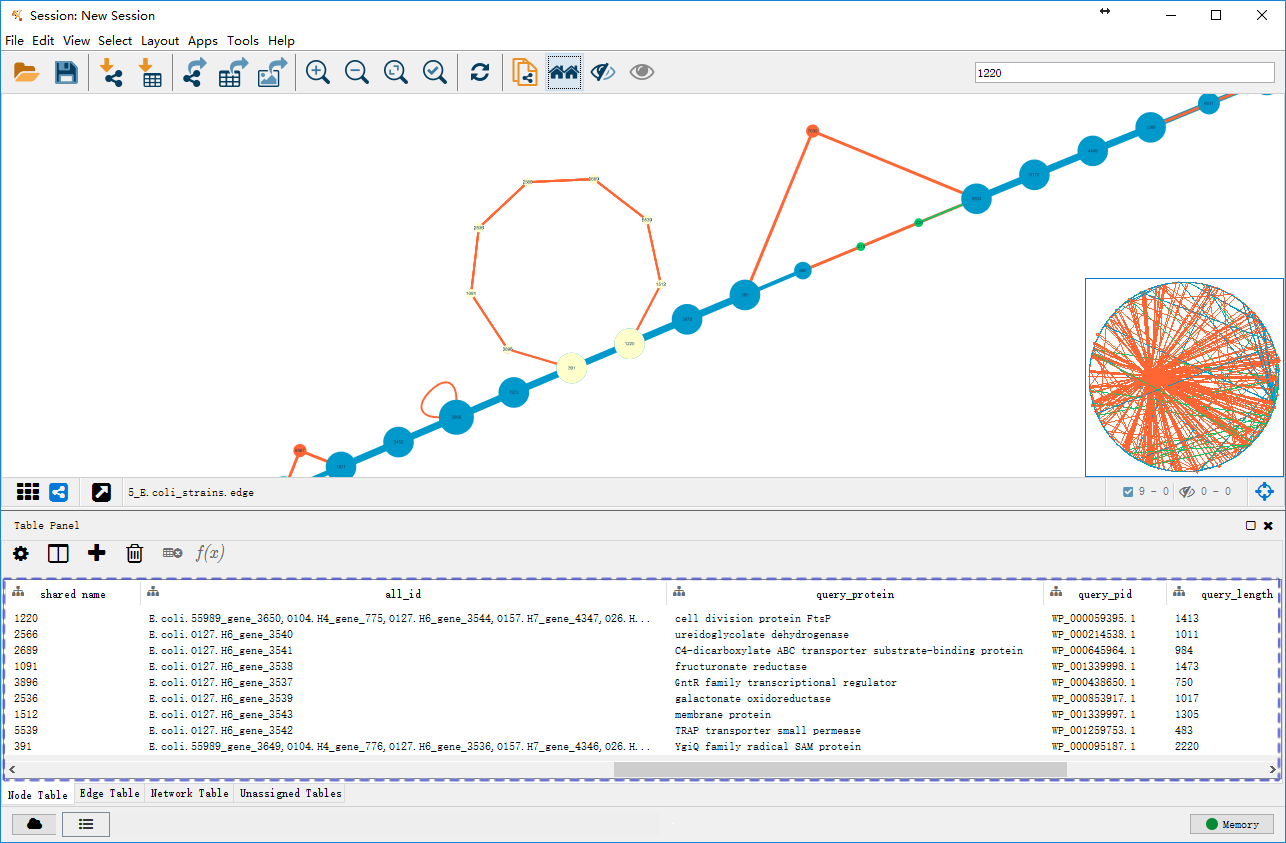

Supplement: Supplemental Files [file giy121_supplemental_files.zip › Supplementary File S1.docx]
