# Supplementary material for: MetaPGN: a pipeline for construction and graphical visualization of annotated pangenome networks
Source: Gigascience. 2018 Oct 2;7(11):giy121. doi: 10.1093/gigascience/giy121 (PMC6251982; doi:10.1093/gigascience/giy121)
Supplement: Supplemental Files [file giy121_supplemental_files.zip › Supplementary File S2.docx]

**Supplementary Text 3. Steps for selecting query-specific nodes in a region between two shared nodes for arrangement.**

1. Starting from the first node and following the ascending order in the reference, find a shared node that links with query-specific node(s), as a starting point.
2. From the query-specific node(s) linking with the shared node(s), find all paths to other shared nodes by depth-first search. Define the farthest shared node as a candidate ending point.
3. If there are other query-specific node(s) linking with the candidate ending point, or there are other query-specific node(s) linking with shared node(s) between the starting point and the candidate ending point, repeat (iii) until all shared nodes in the paths are not linking to any query-specific nodes. Define the farthest shared node as a final ending point.
4. If there are less than a certain number (in this instance, 30) of shared or reference-specific nodes between the starting and final ending point, arrange all query-specific nodes between these two points **as follows**, else skip the starting point and find another starting point forward.

**Supplementary Text 4.** **Comparison of the reference pangenome network (RPGN) and the query pangenome network (QPGN).**

We introduced two indices, conformity and divergence, to assess the accuracy of the gene alignment-based strategy for assembly recruitment. Conformity, the ratio of nodes or edges in the RPGN that recovered by the QPGN of total nodes or edges in the RPGN, is determined by formula 1. Divergence, the ratio of nodes or edges in the QPGN that shared with the RPGN of total nodes or edges in the QPGN, is calculated by formula 2.

$Conformity=\frac{N_{ref\sim query}}{N_{ref}}$, (1)

$Divergence=1-\frac{N_{query\sim ref}}{N_{query}}$, (2)

where $N_{ref\sim query}$ is the number of nodes or edges of the RPGN that can be found in the QPGN, $N_{query\sim ref}$ is the number of nodes or edges of the QPGN that can be found in the RPGN, and $N_{ref}$ and $N_{query}$ represents the total number of all nodes or edges in the RPGN and the QPGN, respectively (see Supplementary Text 4 for detailed definitions).

**Supplementary Text 5.** **Detailed definitions of conformity and divergence.**

Conformity indicates how well a query pangenome network recovers nodes or edges contained in the reference pangenome network (formula S1 and S3 for nodes and edges, respectively), whereas divergence indicates how much a query pangenome network diverges from the reference one (formula S2 and S4 for nodes and edges, respectively).

$Conformity(node)=\frac{rnc}{rnt}$ (S1)

$Divergence(node)=1-\frac{qnc}{qnt}$ (S2)

$Conformity(edge)=\frac{rec}{ret}$ (S3)

$Divergence(edge)=1-\frac{qec}{qet}$ (S4)

where,

**qnt** (query, node, total): the number of query-specific and shared nodes in the query pangenome network.

**qnc** (query, node, common): the number of query-specific and shared nodes in the query pangenome network, which can match nodes (with ≥95% identity and ≥90% overlap for the shorter ones) in the reference pangenome network.

**rnt** (reference, node, total): the number of query-specific and shared nodes in the reference pangenome network.

**rnc** (reference, node, common): the number of query-specific and shared nodes in the reference pangenome network, which can match nodes in the query pangenome network.

**qet** (query, edge, total): the number of query-specific and shared edges in the query pangenome networks.

**qec (**query, edge, common): the number of query-specific and shared edges in the query pangenome networks, whose source and target nodes can match adjacent nodes in the reference pangenome network.

**ret** (reference, edge, total): the number of query-specific and shared edges in the reference pangenome network.

**rec** (reference, edge, common): the number of query-specific and shared edges in the reference pangenome network, whose source and target nodes can match adjacent nodes in the query pangenome network.
