# Supplementary figures and images for: MetaPGN: a pipeline for construction and graphical visualization of annotated pangenome networks
Source: Gigascience. 2018 Oct 2;7(11):giy121. doi: 10.1093/gigascience/giy121 (PMC6251982; doi:10.1093/gigascience/giy121)

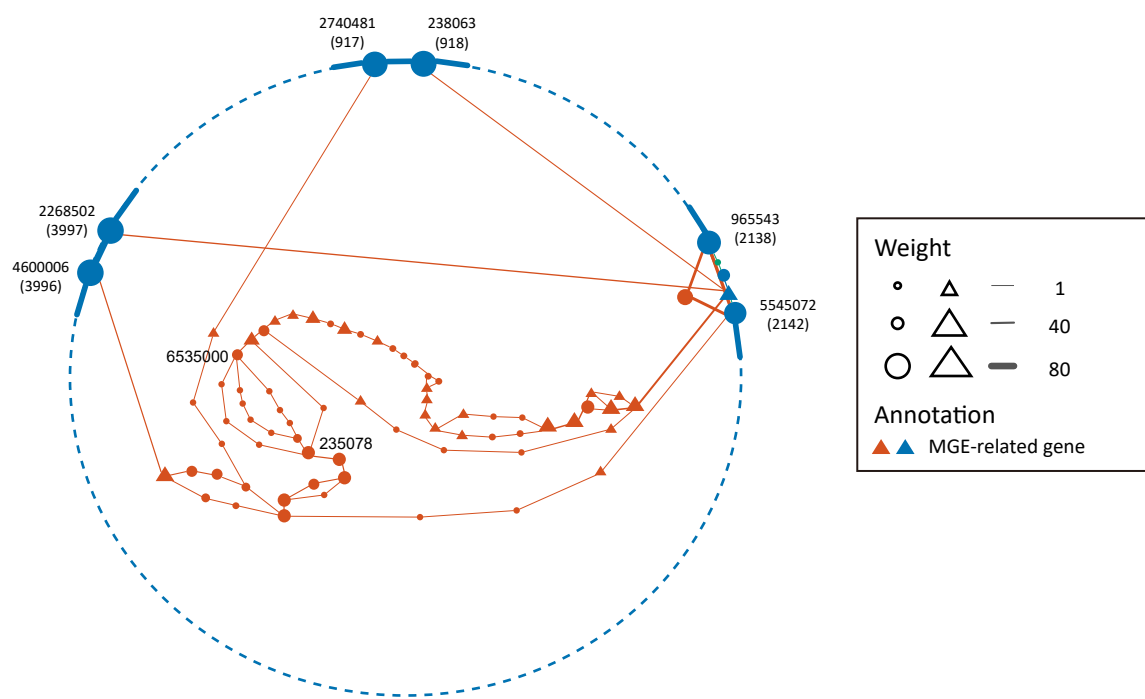

Supplement: Supplemental Files [file giy121_supplemental_files.zip › Supplementary Figure S1.pdf]

a

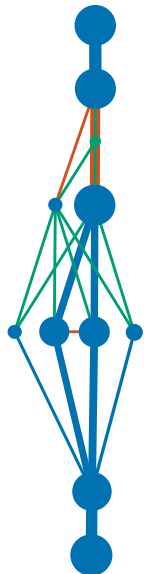

b

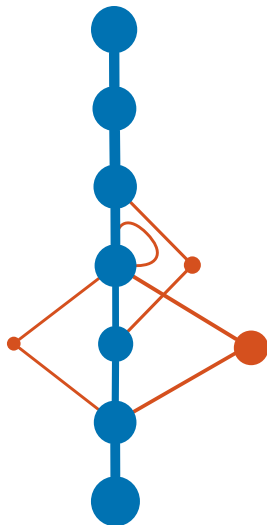

c

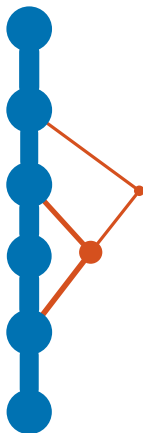

d

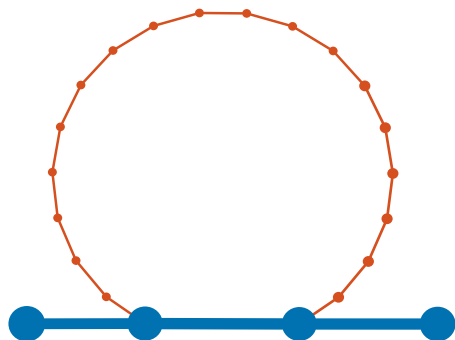

e

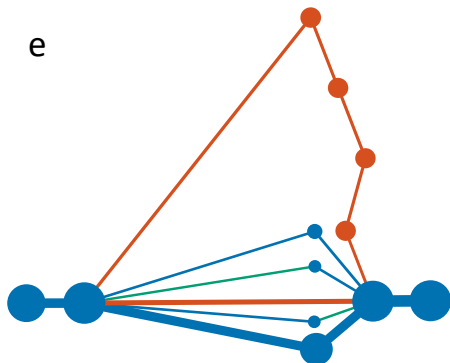

Supplement: Supplemental Files [file giy121_supplemental_files.zip › Supplementary Figure S2.pdf]

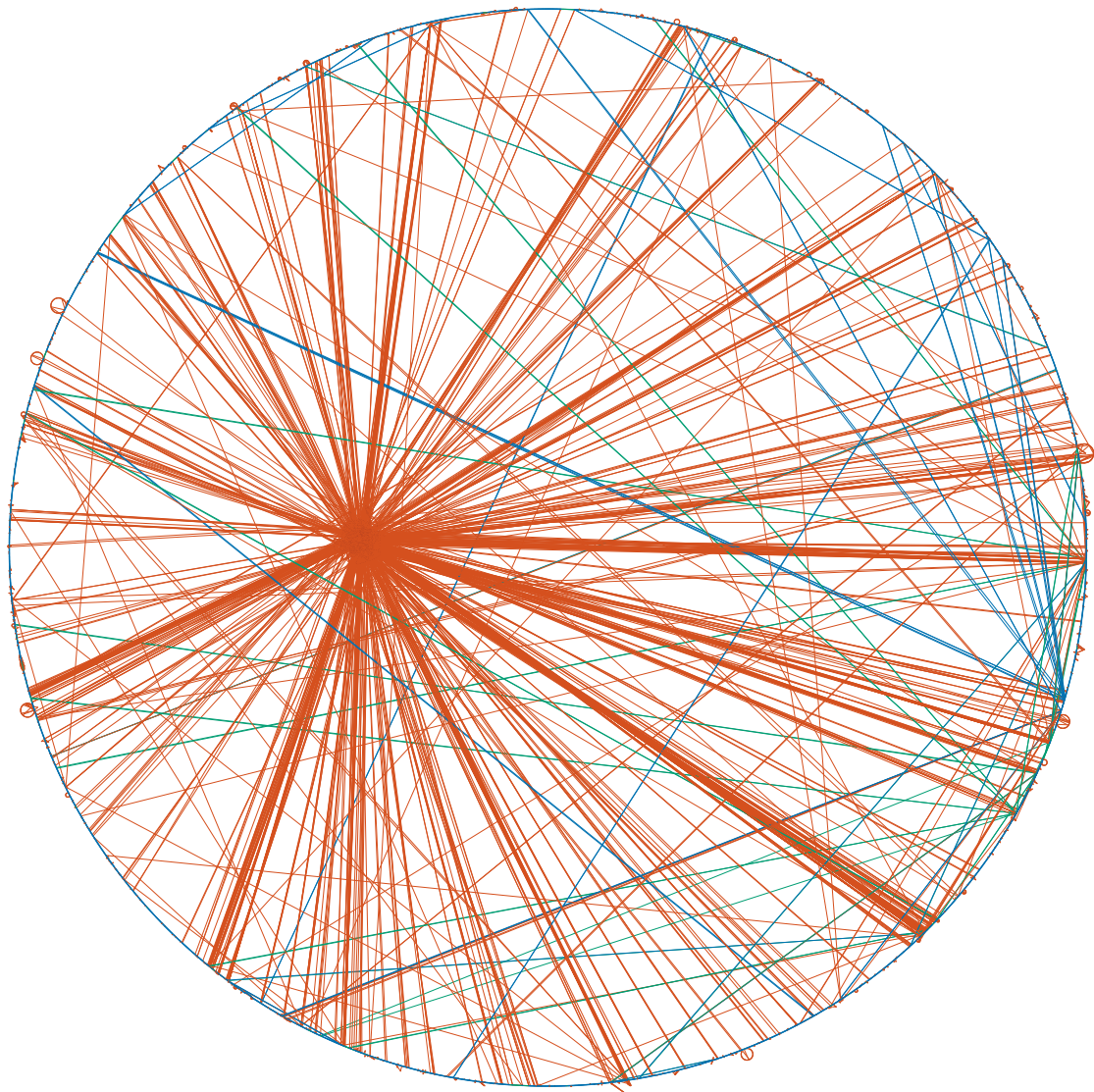

Supplement: Supplemental Files [file giy121_supplemental_files.zip › Supplementary File S3.pdf]

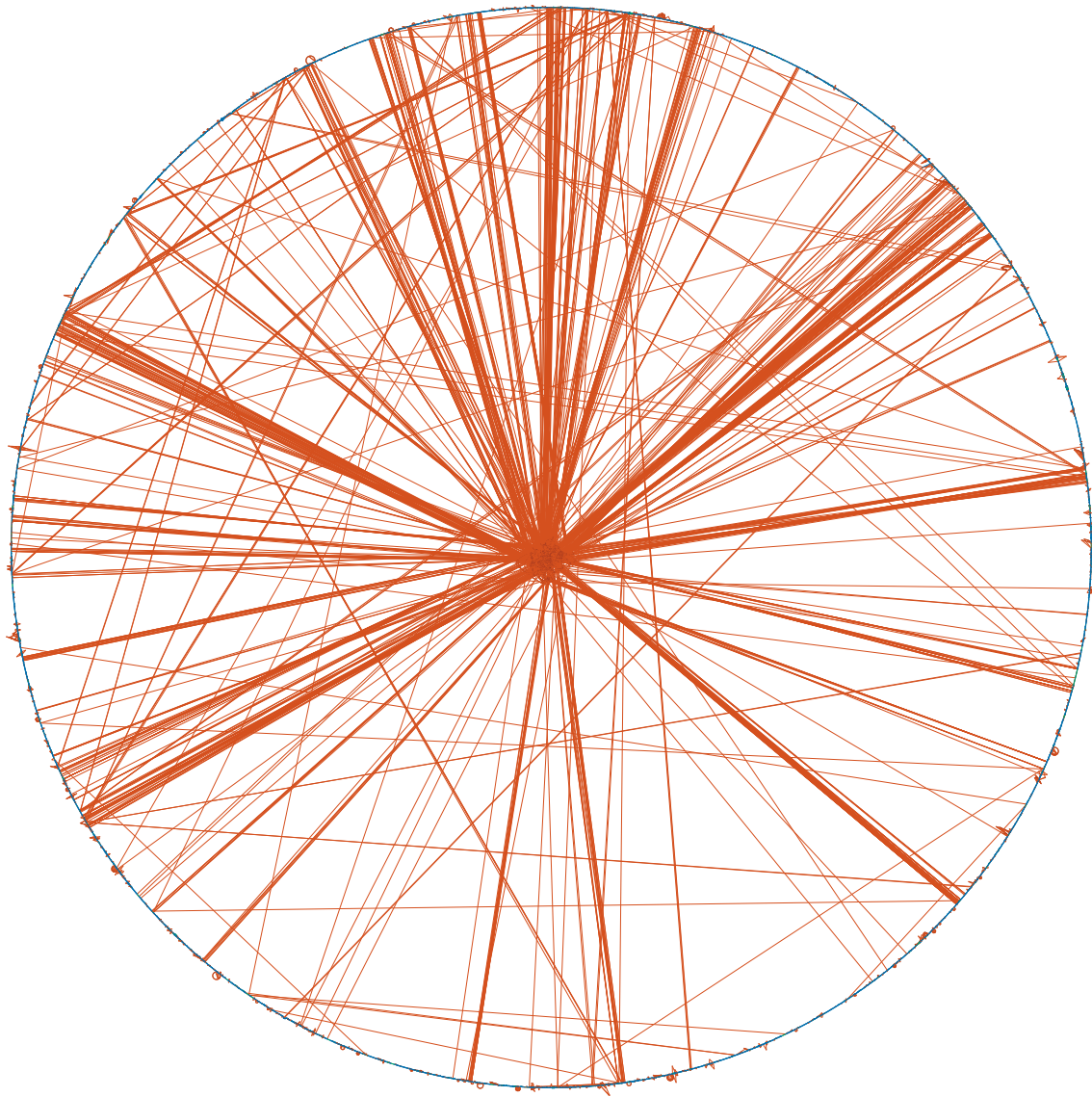

Supplement: Supplemental Files [file giy121_supplemental_files.zip › Supplementary File S4.pdf]
