# Supplementary material for: MetaPGN: a pipeline for construction and graphical visualization of annotated pangenome networks
Source: Gigascience. 2018 Oct 2;7(11):giy121. doi: 10.1093/gigascience/giy121 (PMC6251982; doi:10.1093/gigascience/giy121)
Supplement: Response_to_Reviewer_Comments_Original_Submission.pdf [file giy121_response_to_reviewer_comments_original_submission.pdf]

Author's Response To Reviewer Comments

Close

We appreciate the time and efforts by the editors and referees in reviewing this manuscript. We thank the referees for their helpful comments and suggestions. The manuscript has now been carefully revised to address all their concerns (major revisions are denoted in blue). In order to try the double-blind peer reviewing process, author information, authors' contribution and acknowledgement parts have been removed from the text. We believed that the revised version should meet the journal publication requirements. See the supplementary file named PBP_responses_to_editor_and_reviewers.docx for detailed point-by-point responses.

Close
