# Supplementary material for: MetaPGN: a pipeline for construction and graphical visualization of annotated pangenome networks
Source: Gigascience. 2018 Oct 2;7(11):giy121. doi: 10.1093/gigascience/giy121 (PMC6251982; doi:10.1093/gigascience/giy121)
Supplement: Response_to_Reviewer_Comments_Revision_1.pdf [file giy121_response_to_reviewer_comments_revision_1.pdf]

Author's Response To Reviewer Comments

Close

We appreciate the time and efforts by the editors and reviewers in reviewing this manuscript. We also thank the editors and reviewers for their helpful comments and suggestions. The manuscript has now been carefully revised to address all their concerns. We believed that the revised version should meet the journal publication requirements. See the supplementary file named PBP_responses_to_editor_and_reviewers.R2.docx for detailed point-by-point responses. Thanks again.

Close
