# Supplementary material for: MetaPGN: a pipeline for construction and graphical visualization of annotated pangenome networks
Source: Gigascience. 2018 Oct 2;7(11):giy121. doi: 10.1093/gigascience/giy121 (PMC6251982; doi:10.1093/gigascience/giy121)
Supplement: Reviewer_1_Report_(Original_Submission) -- Alexander Herbig — 05/27/2018 Reviewed [file giy121_reviewer_1_report_(original_submission).pdf]

Reviewer Report

Title**MetaPGN: a pipeline for construction and graphical visualization of annotated pangenome networks**

Version: Original Submission **Date:** 27/05/2018

Reviewer name: **Alexander Herbig**

Reviewer Comments to Author:

The manuscript "Construction and visualization of annotated pangenome networks enable graphical analyses for prokaryotic genetic diversity" describes a computational pipeline (MetaPGN) for the construction and visualization of pangenome networks based on prokaryotic genomic or metagenomic data.
MetaPGN includes a Cytoscape plugin in order to provide an interactive visualization of the resulting network.
The authors demonstrate their pipeline by application to the pangenome analysis of E. coli based on full genomes as well as metagenomes.

The manuscript is well written and the authors convincingly show that MetaPGN is able to support researchers in exploring bacterial pangenomes.

I have a number of comments and suggestions.

In the introduction the authors cite a selection of other tools related to pangenome analysis and elaborate on their shortcomings.
I cannot fully follow the line of argumentation here.
In particular, what do the authors exactly mean when stating "However, these tools visualize the core/accessory sequences or genes on independent lines and thus cannot intuitively present the complex variations among the input genomes."?

The authors describe one important feature of MetaPGN to be able to consider "gene physical adjacency" or "genomic context".
I am wondering why the authors do not properly introduce and make use of the term "synteny".

The authors state that they found fliC and fliD to be highly variable among the analysed strains.
However, I cannot easily see how this can be directly seen from the visualization (Figures 2, 3).
Due to the chosen threshold and the high variability multiple nodes were constructed for these genes, but actually one could argue that only one node should have been constructed.
My point is that if these genes would not be well annotated, one would not be able to see that multiple nodes refer actually to the same gene.
I suggest to discuss this a bit more, e.g. in the same context where the usage of a fixed sequence identity threshold is discussed.
Do the authors consider to include a functionality for manually merging nodes?
In this context I would like to point out another tool, Pan-Tetris (Hennig et al. 2015), which aims at dealing with this challenge.

I noticed that 'pangenome' is spelled inconsistently in the manuscript (also used: 'pan-genome').

I have one further suggestion.
Of course this is completely up to the authors, but in my humble opinion the title could be improved.
In software publications the name of the tool often is part of the title (e.g. "MetaPGN: ...").
In addition, I am wondering if the second part of the title ("enable...") is really needed.

Methods

Are the methods appropriate to the aims of the study, are they well described, and are necessary controls included?

Conclusions

Are the conclusions adequately supported by the data shown?

Reporting Standards

Does the manuscript adhere to the journal's guidelines on minimum standards of reporting? Choose an item.

Statistics

Are you able to assess all statistics in the manuscript, including the appropriateness of statistical tests used?

Quality of Written English

Please indicate the quality of language in the manuscript:

Declaration of Competing Interests

Please complete a declaration of competing interests, considering the following questions:

- Have you in the past five years received reimbursements, fees, funding, or salary from an organisation that may in any way gain or lose financially from the publication of this manuscript, either now or in the future?
- Do you hold any stocks or shares in an organisation that may in any way gain or lose financially from the publication of this manuscript, either now or in the future?
- Do you hold or are you currently applying for any patents relating to the content of the manuscript?
- Have you received reimbursements, fees, funding, or salary from an organization that holds or has applied for patents relating to the content of the manuscript?
- Do you have any other financial competing interests?
- Do you have any non-financial competing interests in relation to this paper?

If you can answer no to all of the above, write 'I declare that I have no competing interests' below. If your reply is yes to any, please give details below.

I declare that I have no competing interests

I agree to the open peer review policy of the journal. I understand that my name will be included on my report to the authors and, if the manuscript is accepted for publication, my named report including any attachments I upload will be posted on the website along with the authors' responses. I agree for my report to be made available under an Open Access Creative Commons CC-BY license (http://creativecommons.org/licenses/by/4.0/). I understand that any comments which I do not wish to be included in my named report can be included as confidential comments to the editors, which will not be published.

I agree to the open peer review policy of the journal

To further support our reviewers, we have joined with Publons, where you can gain additional credit to further highlight your hard work (see: https://publons.com/journal/530/gigascience). On publication of this paper, your review will be automatically added to Publons, you can then choose whether or not to claim your Publons credit. I understand this statement.

Yes
