# Supplementary material for: MetaPGN: a pipeline for construction and graphical visualization of annotated pangenome networks
Source: Gigascience. 2018 Oct 2;7(11):giy121. doi: 10.1093/gigascience/giy121 (PMC6251982; doi:10.1093/gigascience/giy121)
Supplement: Reviewer_2_Report_(Original_Submission) -- Andre Mu — 07/05/2018 Reviewed [file giy121_reviewer_2_report_(original_submission).pdf]

Reviewer Report

Title: **MetaPGN: a pipeline for construction and graphical visualization of annotated pangenome networks**

Version: **Original Submission** **Date:** 7/5/2018

Reviewer name: **Andre Mu**

Reviewer Comments to Author:

The authors set out to describe a bioinformatic tool (MetaPGN) developed for the analysis of metagenomic data; specifically, MetaPGN aims to reconstruct the pangenome (core and accessory genes) of bacterial species from metagenomes given reference genomes as input. The output of MetaPGN is a network of associated genes visualised using a custom cytoscape plugin. The authors used Escherichia coli as an example input reference in which to build a the pangenome.

1) Are the methods appropriate to the aims of the study, are they well described, and are necessary controls included?
The bioinformatic methods appear to be appropriate for the aim of the study, which is to reconstruct the pangenome of a given bacterial species from metagenome datasets. While there are mock metagenomes as controls, a major concern I have is the lack of reference genomes that are representative of different bacterial species used to demonstrate the utility of MetaPGN. For example, how can users be sure that the reconstructed pangenome of Escherichia coli does not include genes derived from other closely related Enterobacteriaceae species (e.g., Enterobacter, Klebsiella, Salmonella)?

2) Are the conclusions adequately supported by the data shown?
Unfortunately, I was unable to connect to the private FTP server (using multiple methods) to retrieve supporting data (Figures and Tables), and the sequence data was not made available through EBI at the time of review. However - independent of data availability - I don't think the conclusions can be fully supported by the results described in the current manuscript for the reason that different bacterial species were not tested as reference genomes.

3) Please indicate the quality of language in the manuscript. Does it require a heavy editing for language and clarity?
The overall quality of language in the manuscript is good. However, minor edits are required to improve the clarity of the manuscript. For example,

Page 8 line 48-51
"Genomic sequences assembled from metagenomic data are usually fragmentary resulted from limitations in sequencing platforms and bioinformatic algorithms"
The meaning/definition of "fragmentary resulted" is not immediately clear.

Page 10 line 12-20
"If extended, genomic adjacency may help in finding possible functional sequences which potentially drive variations such as genomic repeats discussed by Delihas [32] and Wang et al[33], and lead to the discovery of new functional modules as illustrated by Doron et al [34]".
The readability and comprehension of the manuscript would be greatly improved if the authors were to outline the details of each study referenced, instead of referring readers to the citations.

The authors state on page 8 lines 40 - 42 (last sentence of fourth paragraph of discussion) that "MetaPGN can therefore be used to build a pangenome network of these disease-related microbes, and study the genetic variations which might explain these inconsistent findings".

The quality of the manuscript can be improved if the authors were to provide an example of a specific genomic variant causing differential host metabolic response, and a statement of the implications of such findings.

4) Are you able to assess all statistics in the manuscript, including the appropriateness of statistical tests used?
I was not able to assess the program MetaPGN as the sequence data (e.g., mock communities) were not provided at the time of review. Furthermore the accessibility of MetaPGN for end-users would be greatly improved if it were, for example, compiled as a conda package.

Overall, I believe the authors have a good scientific idea - that is, attempting to identify/annotate genes with unknown function/activity by association (networks) with previously annotated genes.

Level of Interest

Please indicate how interesting you found the manuscript: Choose an item.

Quality of Written English

Please indicate the quality of language in the manuscript: Choose an item.

Declaration of Competing Interests

Please complete a declaration of competing interests, considering the following questions:

- Have you in the past five years received reimbursements, fees, funding, or salary from an organisation that may in any way gain or lose financially from the publication of this manuscript, either now or in the future?
- Do you hold any stocks or shares in an organisation that may in any way gain or lose financially from the publication of this manuscript, either now or in the future?
- Do you hold or are you currently applying for any patents relating to the content of the manuscript?
- Have you received reimbursements, fees, funding, or salary from an organization that holds or has applied for patents relating to the content of the manuscript?
- Do you have any other financial competing interests?
- Do you have any non-financial competing interests in relation to this paper?

If you can answer no to all of the above, write 'I declare that I have no competing interests' below. If your reply is yes to any, please give details below.

I declare that I have no competing interests

I agree to the open peer review policy of the journal. I understand that my name will be included on my report to the authors and, if the manuscript is accepted for publication, my named report including any attachments I upload will be posted on the website along with the authors' responses. I agree for my report to be made available under an Open Access Creative Commons CC-BY license (http://creativecommons.org/licenses/by/4.0/). I understand that any comments which I do not wish to

be included in my named report can be included as confidential comments to the editors, which will not be published.

I agree to the open peer review policy of the journal

To further support our reviewers, we have joined with Publons, where you can gain additional credit to further highlight your hard work (see: https://publons.com/journal/530/gigascience). On publication of this paper, your review will be automatically added to Publons, you can then choose whether or not to claim your Publons credit. I understand this statement.

Yes
