# Supplementary material for: MetaPGN: a pipeline for construction and graphical visualization of annotated pangenome networks
Source: Gigascience. 2018 Oct 2;7(11):giy121. doi: 10.1093/gigascience/giy121 (PMC6251982; doi:10.1093/gigascience/giy121)
Supplement: Reviewer_2_Report_Revision_1 -- Andre Mu — 08/22/2018 Reviewed [file giy121_reviewer_2_report_revision_1.pdf]

Reviewer Report

Title: **MetaPGN: a pipeline for construction and graphical visualization of annotated pangenome networks**

Version: **Revision 1** **Date:** 8/22/2018

Reviewer name: **Andre Mu**

Reviewer Comments to Author:

Please consider the following suggestions to assist in readability of manuscript:

Page 6 line 28: "…essential for efficient storage and …"

Page 8 lines 27 - 30: "The two wcaH genes may result in different functions for CA formation and novel survival mechanisms, despite sharing a high degree of similarity (99.1% identity)".

Page 8 line 60 - page 9 line 4: Please state what the alignment-based strategy is?

Page 10 lines 41 - 60 :
"For instance, we found that nucleotide sequences of the fliC gene, which carries H-antigen specificity, were highly divergent among the five pathogenic E. coli assemblies (Fig. 2a)."

"In addition, genes required for the synthesis of O-antigen and outer membrane proteins showed greater diversity in the pangenome network of the five E. coli strains."

"These results are in agreement with previous findings on H-, and O-antigen specificity related genes [26-30]. We also showed that the locations of genes of unknown function are identified when gene adjacency is incorporated into the construction and visualization of pangenomes; this may be helpful for the inference of their biological functions."

Page 10 lines 58 - 60 "For example, in both the two pangenome networks, we found genes of unknown function located between the fliC gene and other flagellin-related genes…"
There is redundancy when reading "..in both the two.."; please revise to "...in both pangenome networks.."

Page 11 line 15: please replace "if extended" with "furthermore"

Page 11 line 40 to page 12 line 28
"Genomic variants of intestinal bacteria were previously found to be correlated with different diseases. For example, the inclusion of a pathogenicity island (BfPAI) in Bacteroides fragilis (B. fragilis) distinguished enterotoxigenic strains (ETBF) from nontoxigenic strains (NTBF) by the ability of ETFBF to secrete a zinc-dependent metalloprotease toxin that can induce inflammatory diarrhea and even colon carcinogenesis. Furthermore, Scher et al. performed shotgun sequencing on fecal samples from newly-onset untreated rheumatoid arthritis (NORA) patients and healthy individuals, and identified several NORA-specific Prevotella copri genes [39]. Hence, pangenome networks built from metagenomes of patients and healthy subjects may aid in detecting associated genomic variants of a certain species.
It should be noted that, in this pipeline we compare genes depending on nucleotide-level sequence identity and overlap: genes with ≥ 95% identity and ≥ 90% overlap are regarded as the same gene. However, genes sharing the same function may not satisfy this criterion (≥95% identity and ≥90% overlap), and protein encoded by these genes may exhibit more similarity due to different codon usage. Therefore, we

intend to cluster genes by comparing their nucleotide sequences as well as the amino acid sequences in future developments of MetaPGN. Furthermore, the current MetaPGN pipeline does not consider other genomic features or physical distances between genes in constructing the pangenome network. Thus, differences in other genomic features such as ribosomal binding site (RBS) sequences [40,41] and distances between the RBS and start codons [42] may result in distinct phenotypes. Accordingly, users may include such information when analyzing pangenome networks. To conclude, MetaPGN enables direct illustration of genetic diversity of a species in pangenome networks, and improving our understanding of genotype-phenotype relationships and the evolutionary history of microorganisms."

Methods section needs consistency in use of parentheses to indicate numbered steps in workflow.

Page 12 line 33: Please remove "First, .."

Page 14 line 6: please replace ".. or else" with "alternatively"

Page 14 line 30 - 34: please rephrase as "the following parameters were chosen for recruitment of metagenome assemblies in this study: c = 3 paired with r = 0.5 …."

Page 14 line 45: Please replace "E. coli-closely related species" with "closely related Enterobacteriaceae species"

Page 15 line 12 - 14: Please state what the traditional genome alignment-based strategy is

Level of Interest

Please indicate how interesting you found the manuscript: Choose an item.

Quality of Written English

Please indicate the quality of language in the manuscript: Choose an item.

Declaration of Competing Interests

Please complete a declaration of competing interests, considering the following questions:

- Have you in the past five years received reimbursements, fees, funding, or salary from an organisation that may in any way gain or lose financially from the publication of this manuscript, either now or in the future?
- Do you hold any stocks or shares in an organisation that may in any way gain or lose financially from the publication of this manuscript, either now or in the future?
- Do you hold or are you currently applying for any patents relating to the content of the manuscript?
- Have you received reimbursements, fees, funding, or salary from an organization that holds or has applied for patents relating to the content of the manuscript?
- Do you have any other financial competing interests?
- Do you have any non-financial competing interests in relation to this paper?

If you can answer no to all of the above, write 'I declare that I have no competing interests' below. If your reply is yes to any, please give details below.

I declare that I have no competing interests

I agree to the open peer review policy of the journal. I understand that my name will be included on my report to the authors and, if the manuscript is accepted for publication, my named report including any attachments I upload will be posted on the website along with the authors' responses. I agree for my report to be made available under an Open Access Creative Commons CC-BY license (http://creativecommons.org/licenses/by/4.0/). I understand that any comments which I do not wish to be included in my named report can be included as confidential comments to the editors, which will not be published.

I agree to the open peer review policy of the journal

To further support our reviewers, we have joined with Publons, where you can gain additional credit to further highlight your hard work (see: https://publons.com/journal/530/gigascience). On publication of this paper, your review will be automatically added to Publons, you can then choose whether or not to claim your Publons credit. I understand this statement.

Yes
