# Supplementary material for: MetaPGN: a pipeline for construction and graphical visualization of annotated pangenome networks
Source: Gigascience. 2018 Oct 2;7(11):giy121. doi: 10.1093/gigascience/giy121 (PMC6251982; doi:10.1093/gigascience/giy121)
Supplement: Supplemental Files [file giy121_supplemental_files.zip › PBP_responses_to_editor_and_reviewers.R2.docx]

We appreciate the time and efforts by the editors and referees in reviewing this manuscript. We thank the referees for their helpful comments and suggestions. The manuscript has now been carefully revised to address all their concerns and we believed that the revised version would meet the journal publication requirements.

For editor’s comment:

“The reviewers were unable to resolve the host for http://exon.gatech.edu/license_download.cgi (as per github page). However, MetaGeneMark can also be downloaded from here:
https://www.qiagenbioinformatics.com/plugins/metagenemark/. So you must update this in the Readme file in your GitHub account. Also, ideally for reproducibility, we suggest you also make this program available as a conda or brew install.”

We thank the editor again for carefully handling the process. We did research and found that <http://exon.gatech.edu/license_download.cgi> is the only way to download the source code (https://www.qiagenbioinformatics.com/plugins/metagenemark/ for downloading complied Workbench or Server). We are sorry that this server was down (as it happened before <https://github.com/marbl/metAMOS/issues/94>) at the time of reviewing. We reported the issue to its owner and were informed that there were issues with the hardware then. Now the server is back online again.

We thank the editor for suggesting making the program available as a conda or brew install. However, for the updated pipeline, apart from getting the pipeline from Github, users only need to download MetaGeneMark and Cytoscape from their official websites. Given that the MetaGeneMark (along with a license file) should be and must be downloaded by users, and the pipeline does not require compilation of the source codes, and additionally, the Github repository automatically records changes in different versions, we think it might not be necessary to compile it as another type of package.

“In addition, please register any new software application in the SciCrunch.org database to receive a RRID (Research Resource Identification Initiative ID) number, and include this in your manuscript. This will facilitate tracking, reproducibility and re-use of your tool.”
This pipeline has been packed and submitted to SciCrunch as a resource (SCR_016472, a resubmitted one), and the current status of the submission is “Curated”. We contacted with colleagues of SciCrunch and were informed that it will soon be public.

For reviewers’ comments:
Reviewer #1:

“The authors have submitted a revised version of their manuscript. The manuscript has been improved significantly.All comments were sufficiently addressed.

As a minor point that needs to be addressed I would like to indicate that MetaGeneMark cannot be downloaded from the link provided in the MetaPGN GitHub Readme file.”

We thank the reviewer again for carefully reviewing the manuscript.

We reported the issue to its owner and were informed that there were issues with the hardware then. Now the server is back online again.

Reviewer #2: 
“Please consider the following suggestions to assist in readability of manuscript:”
We thank the reviewer for pointing these out. We have modified each mentioned sentence/paragraph as follows, and modified other parts of the manuscript as well.

“Page 6 line 28: "…essential for efficient storage and …””
We have modified the manuscript as suggested.

“Page 8 lines 27 - 30: "The two wcaH genes may result in different functions for CA formation and novel survival mechanisms, despite sharing a high degree of similarity (99.1% identity)”.
We have modified the manuscript as suggested.

“Page 8 line 60 - page 9 line 4: Please state what the alignment-based strategy is?”
The traditional genome alignment-based strategy is that, considering an assembled sequence is derived from a genome if the sequence aligns with the genome (defined by a combination of cutoffs, e.g., identity>=95% and overlap>=90%).

Given that basic elements in a pangenome network are genes (nodes), to exploit information generated in gene redundancy elimination and to reduce computation time, we introduce here the gene alignment-based strategy, in which an assembled sequence is considered to be similar to a reference genome, if the sequence containing enough numbers of genes (c=3) and enough fraction of these genes are similar to genes on the reference genome (r=0.5).

We have clarified these definitions in the manuscript.

“Page 10 lines 41 - 60: ‘For instance, we found that nucleotide sequences of the fliC gene, which carries H-antigen specificity, were highly divergent among the five pathogenic E. coli assemblies (Fig. 2a)." 
"In addition, genes required for the synthesis of O-antigen and outer membrane proteins showed greater diversity in the pangenome network of the five E. coli strains." 
"These results are in agreement with previous findings on H-, and O-antigen specificity related genes [26- 30]. We also showed that the locations of genes of unknown function are identified when gene adjacency is incorporated into the construction and visualization of pangenomes; this may be helpful for the inference of their biological functions." ”
We have modified the manuscript as suggested.

“Page 10 lines 58 - 60 "For example, in both the two pangenome networks, we found genes of unknown function located between the fliC gene and other flagellin-related genes…" 
There is redundancy when reading "..in both the two.."; please revise to "...in both pangenome networks.."”
We have modified the manuscript as suggested.

“Page 11 line 15: please replace "if extended" with "furthermore"”
We have modified the manuscript as suggested.

“Page 11 line 40 to page 12 line 28
"Genomic variants of intestinal bacteria were previously found to be correlated with different diseases. For example, the inclusion of a pathogenicity island (BfPAI) in Bacteroides fragilis (B. fragilis) distinguished enterotoxigenic strains (ETBF) from nontoxigenic strains (NTBF) by the ability of ETFBF to secrete a zinc-dependent metalloprotease toxin that can induce inflammatory diarrhea and even colon carcinogenesis. 
Furthermore, Scher et al. performed shotgun sequencing on fecal samples from newly-onset untreated rheumatoid arthritis (NORA) patients and healthy individuals, and identified several NORA-specific Prevotella copri genes [39]. Hence, pangenome networks built from metagenomes of patients and healthy subjects may aid in detecting associated genomic variants of a certain species. 
It should be noted that, in this pipeline we compare genes depending on nucleotide-level sequence identity and overlap: genes with ≥ 95% identity and ≥ 90% overlap are regarded as the same gene. However, genes sharing the same function may not satisfy this criterion (≥95% identity and ≥90% overlap), and protein encoded by these genes may exhibit more similarity due to different codon usage. Therefore, we intend to cluster genes by comparing their nucleotide sequences as well as the amino acid sequences in future developments of MetaPGN. Furthermore, the current MetaPGN pipeline does not consider other genomic features or physical distances between genes in constructing the pangenome network. Thus, differences in other genomic features such as ribosomal binding site (RBS) sequences [40,41] and distances between the RBS and start codons [42] may result in distinct phenotypes. Accordingly, users may include such information when analyzing pangenome networks. To conclude, MetaPGN enables direct illustration of genetic diversity of a species in pangenome networks, and improving our understanding of genotype-phenotype relationships and the evolutionary history of microorganisms."”
We have modified the manuscript as suggested.

“Methods section needs consistency in use of parentheses to indicate numbered steps in workflow.”

We have modified the manuscript as suggested.

“Page 12 line 33: Please remove "First, .."”
We have modified the manuscript as suggested.

”Page 14 line 6: please replace ".. or else" with "alternatively"“
We have modified the manuscript as suggested.

”Page 14 line 30 - 34: please rephrase as "the following parameters were chosen for recruitment of metagenome assemblies in this study: c = 3 paired with r = 0.5 …." “
We have rephrased this sentence to “The following parameters were chosen for recruitment of metagenome assemblies in this study: c = 3 paired with r = 0.5. These parameters recruit assemblies containing at least 3 genes, including 2 shared genes.”

”Page 14 line 45: Please replace "E. coli-closely related species" with "closely related Enterobacteriaceae species"“
We have replaced this phrase with the suggested one.

”Page 15 line 12 - 14: Please state what the traditional genome alignment-based strategy is“
The traditional genome alignment-based strategy is that, considering an assembled sequence is derived from a genome if the sequence aligns with the genome (defined by a combination of cutoffs, e.g., identity>=95% and overlap>=90%).

Thank you again for handling the paper.

Regards,

All authors.
